# An anionic, endosome-escaping polymer to potentiate intracellular delivery of cationic peptides, biomacromolecules, and nanoparticles

**DOI:** 10.1038/s41467-019-12906-y

**Published:** 2019-11-01

**Authors:** Brian C. Evans, R. Brock Fletcher, Kameron V. Kilchrist, Eric A. Dailing, Alvin J. Mukalel, Juan M. Colazo, Matthew Oliver, Joyce Cheung-Flynn, Colleen M. Brophy, John W. Tierney, Jeffrey S. Isenberg, Kurt D. Hankenson, Kedar Ghimire, Cynthia Lander, Charles A. Gersbach, Craig L. Duvall

**Affiliations:** 1 Department of Biomedical Engineering, Vanderbilt University, 2301 Vanderbilt Place, PMB351826, Nashville, TN 37235 USA; 2 Department of Bioengineering, University of Pennsylvania, 2301 240 Skirkanich Hall, 210S. 33rd Street, Philadelphia, PA 19104-6321 USA; 3 Vanderbilt University School of Medicine, Vanderbilt University, 1161 21st Avenue South # D3300, Nashville, TN 37232 USA; 4 Medical Scientist Training Program, Vanderbilt University School of Medicine, Nashville, TN 37232 USA; 5 Program in Cell and Molecular Biology, Duke University School of Medicine, Durham, NC 27710 USA; 6 Division of Vascular Surgery, Department of Surgery, Vanderbilt University Medical Center, D-5237 Medical Center North, 1161 22nd Avenue South, Nashville, TN 37232 USA; 7 Veterans Affairs Medical Center, VA Tennessee Valley Healthcare System, 1310 24th Avenue South, Nashville, TN 37212 USA; 8 Heart, Lung, Blood and Vascular Medicine Institute, Department of Medicine, University of Pittsburgh, 4200 Fifth Avenue, Pittsburgh, PA 15260 USA; 9 Department of Orthopaedic Surgery, University of Michigan Medical School, 109 Zina Pitcher Place, Ann Arbor, MI 48109 USA; 10 University of Pittsburgh School of Medicine, 3550 Terrace Street, Pittsburgh, PA 15261 USA; 11 Moerae Matrix Inc., 55 Madison Avenue, Suite 400, Morristown, NJ 07960 USA; 12 Center for Genomic and Computational Biology, Duke University, Durham, NC 27710 USA; 13 Department of Biomedical Engineering, Duke University, Durham, NC 27710 USA; 14 Department of Orthopaedic Surgery, Duke University, Durham, NC 27710 USA

**Keywords:** Transfection, Biomaterials, Cell delivery, RNAi

## Abstract

Peptides and biologics provide unique opportunities to modulate intracellular targets not druggable by conventional small molecules. Most peptides and biologics are fused with cationic uptake moieties or formulated into nanoparticles to facilitate delivery, but these systems typically lack potency due to low uptake and/or entrapment and degradation in endolysosomal compartments. Because most delivery reagents comprise cationic lipids or polymers, there is a lack of reagents specifically optimized to deliver cationic cargo. Herein, we demonstrate the utility of the cytocompatible polymer poly(propylacrylic acid) (PPAA) to potentiate intracellular delivery of cationic biomacromolecules and nano-formulations. This approach demonstrates superior efficacy over all marketed peptide delivery reagents and enhances delivery of nucleic acids and gene editing ribonucleoproteins (RNPs) formulated with both commercially-available and our own custom-synthesized cationic polymer delivery reagents. These results demonstrate the broad potential of PPAA to serve as a platform reagent for the intracellular delivery of cationic cargo.

## Abstract (web summary)

Most reagents designed to deliver cargo into cells are cationic and so cannot deliver cationic cargo. Here the authors show that pretreating cells with the anionic polymer poly(propylacrylic acid) facilitates the uptake and endosomal escape of a wide variety of cationic cargo in numerous cell types.

## Introduction

Extracellular targets such as cell surface receptors and secreted factors (cytokines and growth factors) can be efficiently modulated with monoclonal antibodies, which are a significant and rapidly growing sector of the research and pharmaceutical markets. Other types of biologics such as therapeutic peptides and RNA-based formulations for gene therapy, silencing, and editing are being developed for modulation of intracellular targets, especially for research use, but have so far achieved less clinical impact^1^. The tremendous interest in biologic drugs is driven by their advantages over conventional small molecule drugs, namely high selectivity and potency coupled with more predictable behavior and limited side effects. Furthermore, biologics have the ability to modulate previously undruggable targets, including site specific editing of the genome to yield new, more effective therapies for a variety of conditions^2^. However, intracellular-acting peptides, proteins, and nucleic acids are hindered relative to small molecule drugs by their reduced ability to penetrate cell and endolysosomal membranes.

Peptides are attractive as research tools and pharmaceutical agents due to their high-specificity, biocompatibility, and low-cost synthesis. There are currently over 60 FDA approved peptide drugs on the market and over 140 peptide drugs in clinical trials^3^, but essentially all of these target extracellular surface receptors (e.g., G-protein coupled receptors). Despite their lack of clinical development, many peptides have also been discovered with intracellular pharmacological activity, mostly derived from segments of a protein that can recapitulate that protein’s biological activity^4^ or block protein–protein interactions^5^; such peptides can potentially be developed clinically, and they are also an important tool for both target identification and understanding fundamental mechanisms and function of intracellular protein–protein interactions.

Most marketed delivery reagents that seek to unlock the potential of cytosolically active peptides are essentially all polycationic lipids or polymers that can facilitate interactions with inherently negatively charged cellular membranes. These cationic reagents are generally cytotoxic^6^ and have limited capacity to package non-nucleic acid cargo. To overcome this, peptides are fused with cationic sequences called cell penetrating peptides (CPPs)^7–9^ in order to promote electrostatic interactions with the anionic cell membrane and induce cellular uptake. However, most CPPs lack potency (often require >100 µM concentrations), produce transient effects, and suffer from internalization into and entrapment within vesicles of the endolysosomal trafficking pathway^10^ that limit cytosolic bioavailability^11^.

Nucleic acids, including RNA interference and gene editing protein-RNA ribonucleoprotein (RNP) complexes, are another powerful class of biologic modulators with both clinical and research applications. Several RNA-based therapeutics, including the first siRNA, were recently approved by the FDA for clinical use^12^, while gene editing technologies have seen rapidly expanded use in research and drug development over the past 10 years. Viral vectors provide superior efficiency, but concerns remain regarding preexisting immunogenicity, inability to perform repeated dosing due to adaptive immune responses, and the complexity and cost of manufacturing^13^. These limitations motivate the continued pursuit of more efficient, non-viral delivery systems. Physical methods, such as electroporation, are limited by cytotoxicity. Chemical methods generally comprise cationic nano-polyplexes (NPs) formed from polycationic lipids and polymers that can electrostatically condense and facilitate delivery of anionic nucleic acids^14^. Although these formulations show promise, they still lack potency relative to viral vectors due to inferior intracellular bioavailability.

Herein, we investigate cell pretreatment with the anionic polymer poly(propylacrylic acid) (PPAA) for potentiating uptake, endosome escape, and intracellular bioavailability of cationic peptides, proteins, and non-viral siRNA and RNP therapies. The structure of PPAA contains pendant hydrophobic propyl (-C_3_ alkyl) groups that we hypothesized can create an outer cell membrane coating through non-destructive cell membrane intercalation (Supplementary Fig. 1). PPAA also contains a carboxylate anion at each repeat, which we hypothesized would, upon cell membrane coating, enhance the net negative charge on cell surfaces as a mechanism for promoting subsequent attraction of cationic cargo. The carboxylate moieties of PPAA have an acid dissociation constant (pK_a_) of 6.7, which, combined with the pendant, hydrophobic propyl moiety, triggers a solubility switch toward a more hydrophobic and membrane disruptive state upon acidification from extracellular to endolysosomal pH^15^. PPAA has been explored as a component of different types of drug delivery formulations in the past due to its endosome escape capability, but it has not been explored to our knowledge for its cell membrane coating/modification capabilities or as a generally applicable pretreatment strategy to potentiate delivery of subsequently applied cationic molecules or formulations.

This study is inspired by our recent work showing that formulation of cationic vasoactive CPPs with PPAA into premade nano-polyplexes (NPs) increased peptide uptake 35–70 fold, promoted peptide release from endosomes, increased peptide intracellular half-life by over an order of magnitude, and increased tissue level peptide bioactivity in ex vivo human vascular tissue and in vivo transplanted rabbit vascular grafts^16,17^. These previous studies focused solely on pre-formulation of PPAA and therapeutic peptides into electrostatically complexed NPs prior to cell treatment, use of a single PPAA:peptide ratio, and application only within vascular smooth muscle cells/tissues. The current studies were designed to broadly investigate each of these variables and benchmark PPAA against other marketed delivery reagents to establish PPAA as a platform reagent for the intracellular delivery of peptides; these studies also sought to test breadth of this approach for delivery of larger proteins, antisense morpholinos, and lipid- and polymer-based NP formulations of siRNA for gene silencing, and lipid- and polymer-based NP formulations of Cas9/guide RNA RNP complexes for gene editing.

## Results

### Peptide library

To investigate the structural dependence of the cationic CPP sequence on PPAA-mediated peptide uptake, we explored a small library of CPP-modified peptides (Table 1, Supplementary Fig. 2). The first five peptides comprise a MAPKAP Kinase 2 inhibitory peptide (MK2i)^4^ modified with different CPP sequences that are among the most commonly utilized CPPs: the cationic, non-amphipathic CPPs TAT, R6, and YARA, the primary amphipathic CPP penetratin, and the secondary amphipathic CPP transportan. These peptides enable studying the effects of CPP sequence on PPAA-mediated peptide uptake, as we hypothesize that cell delivery is significantly influenced by interactions between the cationic CPP sequence and anionic PPAA. Uptake was also investigated for two additional peptides based upon a phospho-mimetic of the vasodilator-stimulated phosphoprotein (VASP) both with and without the YARA CPP to assess the influence of the peptide sequence attached to a CPP (i.e., YARA-MK2i vs. YARA-VASP) and to validate that the inclusion of a cationic CPP sequence is critical for PPAA-mediated uptake. We synthesized the 11th beta strand of the GFP protein and coupled it to the YARA CPP through an intracellularly reducible disulfide linkage to utilize as a reporter for intracellular peptide bioavailability via a quantitative split-GFP fluorescence transduction assay^18^ and we utilized a peptide comprising the YARA CPP fused to the HiBiT peptide (proprietary sequence not shown) of the NanoLuc platform as an additional intracellular bioavailability readout^19,20^. We also investigated the use of the newer generation, secondary amphipathic CPPs PepFect and CADY as siRNA delivery vectors. All peptides utilized have an isoelectric point (pI = 9–13) that is above the acid dissociation constant of the carboxylate moiety of PPAA (i.e., pK_a_ ~ 6.7), promoting electrostatic peptide-polymer interactions at physiologic pH.

**Table 1:** Characteristics of cell penetrating peptides utilized

Peptide	Sequence (CPP-peptide)	pI^a^	MW (Da)	CPP type	Net charge (at pH 7.0)	Avg. hydrophilicity^b^ (% hydrophilic/total)
YARA-MK2i	YARAAARQARA-KALARQLGVAA	pH 12.4	2283.7	Cationic, non-amphipathic	5	0.1 (32%)
TAT-MK2i	GRKKRRQRRRPPQ-KALARQLGVAA	pH 12.9	2798.3	Cationic, non-amphipathic	10	1.0 (54%)
R6-MK2i	RRRRRR-KALARQLGVAA	pH 12.9	2034.5	Cationic, non-amphipathic	8	1.0 (53%)
Penetratin-MK2i	RQIKIWFQNRRMKWKK-KALARQLGVAA	pH 12.6	3326.1	Primary amphipathic	9	0.1 (46%)
Transportan-MK2i	GWTLNSAGYLLGKINLKALAALAKKIL-KALARQLGVAA	pH 11.3	3920.7	Secondary amphipathic	6	−0.3 (26%)
YARA-VASP	YARAAARQARA-KLRKVS_p_K	pH 12.2	2124.4	Cationic, non-amphipathic	7	0.7 (50%)
VASP (no CPP)	KLRKVS_p_K	pH 11.7	938.1	None	4	1.3 (71%)
YARA-SS-GFP11β	YARAAARQARAC-TIGAANVYEHLVMHDR	pH 9.4	3219.7	Cationic, non-amphipathic	2	0.0 (29%)
PepFect^c^	Stearyl-AGYLLGKLLOOLAAAALOOLL	pH 10.9	2407.2	Stearylated, secondary amphipathic	5	−0.2 (24%)
CADY^c^	Ac-GLWRALWRLLRSLWRLLWRA-cya	pH 12.7	2724.41	Secondary amphipathic	4	−0.6 (30%)

The library of peptides utilized in this work comprising a MAPKAP Kinase 2 inhibitory peptide (MK2i) fused to five different cell penetrating peptide sequences, a phosphopeptide mimetic of vasodilator-stimulated phosphoprotein (VASP) with and without the cell penetrating peptide YARA, a fusion peptide of the YARA CPP conjugated through a disulfide bond to the 11th β strand of the green fluorescent protein (GFP), and the nucleic acid delivery vectors PepFect and CADY

*Sp*  phosphorylated serine, *O* ornithine, *Ac* Acetyl, *cya*  cysteamide

^a^Isoelectric point

^b^Hopp & Woods hydrophilicity scale (Supplementary Fig. 1)

^c^The stearyl modification of PepFect and the cysteamide modification of CADY were not included in pI, net charge, or hydrophilicity calculations presented

### Dose dependency of PPAA-mediated peptide cellular uptake

The influence of the dose of the PPAA polymer and the ratio of PPAA to YARA-MK2i peptide was measured on the intracellular peptide delivery of pre-formed NPs in HCAVSMCs. Investigation of peptide:polymer mass ratios ranging from 3:1 to 1:20 (Supplementary Fig. 3a) demonstrated that a mass ratio of 1:5 (i.e., [PPAA] ~2.5 µM) provides optimal uptake and that peptide uptake decreases at higher polymer doses, potentially due to PPAA-mediated cytotoxicity or limitations in solubility. Notably, a mass ratio of 1:1.2 (our previously identified optimal formulation based on NP size/monodispersity^17^) did not produce the highest cellular uptake. Finally, we investigated whether absolute polymer dose or the peptide:polymer ratio is the key driver of optimal delivery performance. Uptake of 5, 10, and 25 µM YARA-MK2i peptide at mass ratios ranging from 3:1 to 1:20 peptide:polymer demonstrated that maximal peptide uptake consistently occurred at a polymer dose of 2.5–5 µM and was independent of the dose of peptide or mass ratio (Supplementary Fig. 3b).

### Effects of CPP type and PPAA application approach on uptake

**Fig. 1:**
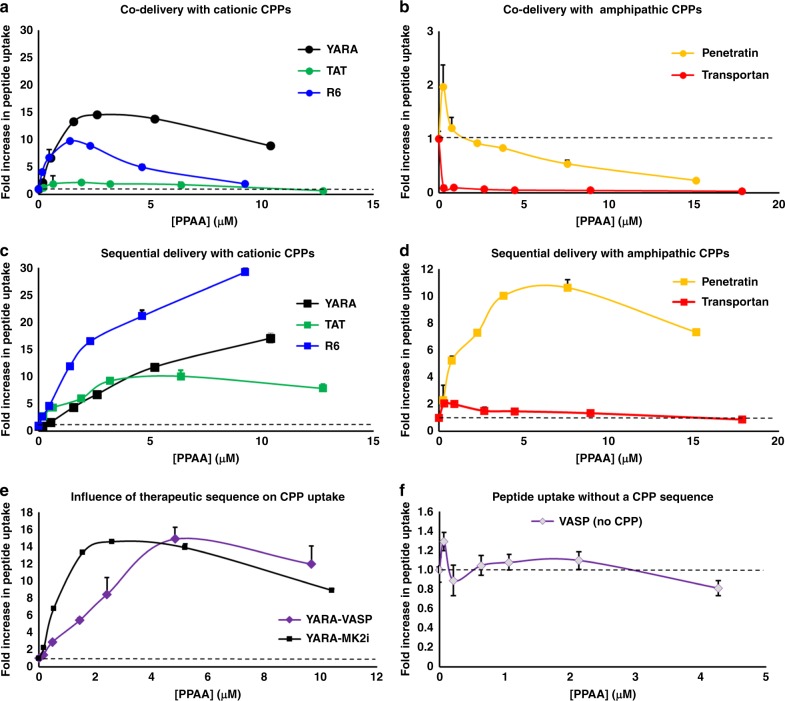
Sequential delivery is effective across all CPP types. Polymer dose-dependent uptake of the MK2i peptide (co-delivery of pre-complexed polymer/peptide) fused to **a** three separate cationic, non-amphipathic CPPs and **b** two different amphipathic CPPs. Sequential polymer then peptide delivery polymer dose-dependent uptake of the MK2i peptide fused to **c** three separate cationic, non-amphipathic CPPs and **d** two different amphipathic CPPs. **e** Polymer dose-dependent uptake of the YARA CPP fused to two separate therapeutic peptide sequences (MK2i and VASP) when co-delivered. **f** Polymer dose-dependent uptake of the VASP peptide without a CPP. The mass ratios used for all data shown are 3:1, 1:1, 1:3, 1:5, 1:10, and 1:20 peptide:polymer. Error bars represent SEM. Non-normalized uptake for all data presented in this figure is shown in Supplementary Fig. 4

Formulation of cationic, non-amphipathic CPP-based peptides (i.e., YARA, TAT, and R6) with PPAA into NPs for co-delivery consistently increased peptide uptake with optimal uptake in HCAVSMCs occurring in the polymer dose range of 2–5 µM (44–110 µg/mL) PPAA (Fig. 1a). However, the two amphipathic CPPs penetratin (primary amphipathic) and transportan (secondary amphipathic) did not display significant PPAA-mediated enhancement of uptake with co-delivery (Fig. 1b). Amphipathic CPPs are internalized through multiple mechanisms involving both electrostatic and hydrophobic interactions with cell membranes. Hydrophobic components of amphipathic CPPs insert into plasma membranes causing uptake and increased membrane permeability through a variety of mechanisms^21^ (e.g., direct translocation through inverted micelle formation, pore formation, the carpet-like model, or the membrane thinning model^9^). We hypothesized that the hydrophobic propyl moiety of PPAA may competitively interact with the hydrophobic domain of these amphipathic CPPs when pre-complexed, thereby hindering their interactions with the cell membrane. To test this hypothesis and determine whether an alternate treatment strategy may achieve PPAA-mediated enhancement of amphipathic CPP uptake, we compared cellular uptake of co-delivery (i.e., pre-complexed NP treatments) with sequential delivery of PPAA alone first, followed by subsequent treatment with the peptide alone. Sequential treatment with the cationic, non-amphipathic CPPs resulted in similar increases in uptake compared with delivery of pre-formed NPs (Fig. 1c). In striking contrast to co-delivery, sequential delivery of PPAA followed by the amphipathic CPPs increased peptide uptake (Fig. 1d). We then performed an uptake study utilizing a VASP peptide with and without the cationic, non-amphipathic CPP YARA. Very similar trends in PPAA dose-dependent uptake of both the YARA-MK2i and YARA-VASP peptides indicate that the functional peptide sequence has little influence on polymer-mediated peptide uptake (Fig. 1e). However, there was no polymer effect on uptake of the VASP peptide not fused with a CPP (Fig. 1f), indicating that the cationic CPP segment is necessary for PPAA enhancement of peptide uptake. We subsequently investigated, for PPAA-peptide co-delivery, whether there is a correlation between peptide uptake enhancement and size, monodispersity, or surface charge of pre-complexed NPs (Supplementary Fig. 5). Results of this study combined with our uptake data indicate that there is no clear relationship between optimal uptake and the physicochemical properties of PPAA-peptide complexes and that optimal uptake is dependent on the concentration of the polymer alone.

### Cell type dependency of peptide uptake with PPAA co-delivery

The dose-dependent uptake of the YARA-MK2i was investigated in five different cell types. Trends in polymer dose-dependent peptide uptake were consistent among all five cell lines, with highly phagocytic macrophages demonstrating increased basal uptake and a peak uptake at a slightly lower polymer dose than smooth muscle and endothelial cells (Fig. S6a). Consistent with the different types of CPPs, there is an apparent optimal dose range of 2–5 µM PPAA that resulted in a ~10–30-fold increase in uptake of the YARA-MK2i peptide in all cell lines. To investigate if the shift in optimal peptide uptake to lower doses of polymer in highly phagocytic macrophages was consistent across CPPs, we analyzed the uptake of the TAT-MK2i peptide in smooth muscle cells compared with macrophages. Similarly, macrophages required a lower polymer dose to achieve maximal TAT-MK2i uptake (Supplementary Figs 6b and 7).

### Comparison of PPAA to commercial agents for CPP delivery

**Fig. 2:**
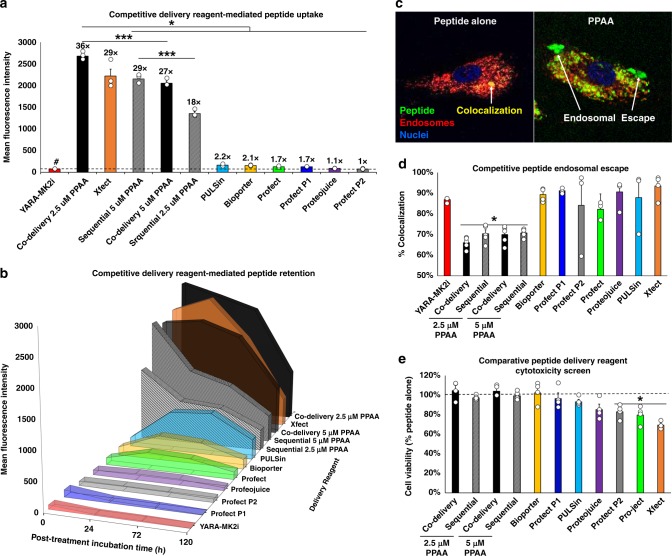
PPAA outperforms commercially available peptide delivery reagents. Comparison of peptide delivery reagent-mediated **a** uptake (numbers above the bars denote the fold increase in peptide uptake compared with the peptide alone) and **b** retention of the YARA-MK2i peptide over time following treatment removal. **c** Representative colocalization images and **d** quantification of peptide colocalization with the endosomal dye LysoTracker Red demonstrating PPAA-mediated endosomal escape and intracellular YARA-MK2i peptide delivery; *p < 0.05 vs. all other treatment groups. **e** Evaluation of delivery reagent-mediated cytotoxicity compared with delivery of the YARA-MK2i peptide alone (10 µM peptide); *p < 0.05 vs. treatment with the peptide alone; statistical analyses—one-way ANOVA followed by Tukey’s post hoc test. Data are presented as means ± SEM graphically

We next completed comparative studies on peptide delivery efficiency and retention time in HCAVSMCs for our system relative to seven commercially available peptide/protein delivery reagents (Supplementary Table 1). Co-delivery of the YARA-MK2i peptide with 2.5 µM PPAA resulted in a ~36-fold increase in peptide uptake, whereas the closest competing reagent, Xfect, achieved a ~29-fold increase in uptake (Fig. 2a). Sequential delivery was also effective (18- and 29-fold increase in uptake for 2.5 and 5 µM PPAA, respectively), albeit less so than co-delivery with PPAA (36- and 27-fold increase in uptake for 2.5 and 5 µM PPAA, respectively). With the exception of Xfect, no competing reagents achieved more than a 2.2-fold increase in peptide uptake. Intracellular peptide retention over a 5-day period following treatment removal was highest for co-delivery with 2.5 µM PPAA, with Xfect performing as the closest competing reagent (Fig. 2b).

We hypothesized that enhanced intracellular retention demonstrated with PPAA-mediated peptide delivery is due to the ability of PPAA to facilitate pH-triggered endolysosomal escape, preventing degradation and/or exocytosis back out of the cell. To test this hypothesis, peptide endosomal escape was evaluated via analysis of fluorescently labeled peptide colocalization with the endosomal dye lysotracker (Fig. 2c). Fluorescence microscopy both further verified superior PPAA-mediated peptide uptake (Supplementary Figs. 8 and 9) and demonstrated that PPAA significantly decreased peptide endosomal entrapment compared with all competing reagents (Fig. 2d).

A cytotoxicity screen was performed on the PPAA reagent and the commercially available reagents to complement the cell delivery study. Co-delivery and sequential delivery with both 2.5 and 5 µM PPAA were found to have no significant effects on cell viability (Fig. 2e). In contrast, Profect P2, Pro-ject, and Xfect (the closest competing reagent in terms of enhancing peptide uptake and retention) all resulted in significant decreases in cell viability 24 h post-treatment (i.e., the same 30 min treatment utilized in uptake studies). The cytotoxic effects of the Xfect reagent were further suggested by the decrease in cell size and enhanced granularity evident from flow cytometric analysis of treated cells (Supplementary Fig. 10). To better define the cytocompatible dose range of PPAA, we also performed a polymer dose-dependent cytotoxicity and membrane permeabilization assays both with and without complexation with the YARA-MK2i peptide across a broader PPAA range (Supplementary Fig. 11). In agreement with our initial hypothesis as to why peptide uptake appeared to consistently saturate and then decrease at doses ≥ 10 µM, PPAA displayed statistically significant cytotoxicity at doses ≥ 10 µM (Supplementary Fig. 11a). Complexation with the YARA-MK2i peptide slightly mitigated polymer-mediated cytotoxicity, shifting the appearance of significant toxicity to higher doses (i.e., ≥20 µM). No significant decreases in membrane integrity were demonstrated at any PPAA doses when analyzing LDH release (Supplementary Fig. 11b), indicating that PPAA-mediated cytotoxicity is not a result of loss of membrane integrity.

### Mechanisms underlying PPAA-mediated CPP uptake

**Fig. 3:**
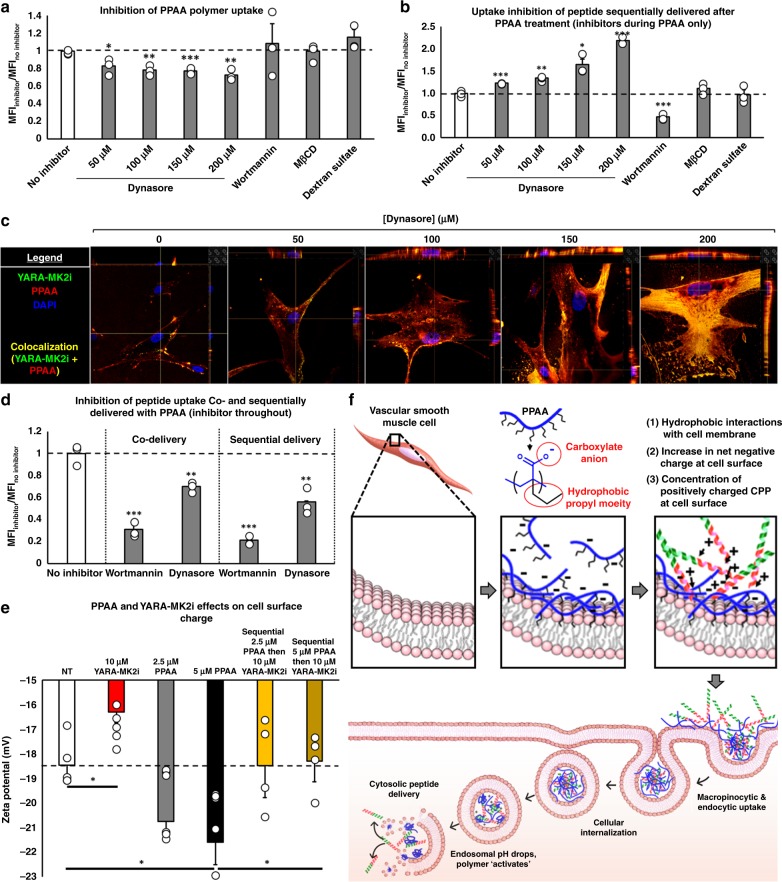
Mechanism of action for PPAA-mediated CPP uptake. **a** Inhibition of rhodamine labeled PPAA polymer uptake in HCAVSMCs. **b** The effect of adding inhibitors during PPAA pretreatment on subsequent uptake of Alexa-488 labeled YARA-MK2i peptide in HCAVSMCs (inhibitors applied during PPAA treatment only). **c** Representative Z-stack images (images have *x* and *y* projections of 3-dimensional z-stack images below and to the right of each image, respectively) of the dose-dependent effects of dynasore during PPAA pretreatment on fluorescently labeled polymer and peptide uptake. **d** The effects of adding inhibitors throughout the entire treatment for both co-delivery and sequential delivery of PPAA and Alexa-488 labeled YARA-MK2i peptide in HCAVSMCs. For **a**, **b**, **d**, *p < 0.05, **p < 0.01, ***p < 0.001 vs. no inhibitor. **e** Zeta potential measurement of PPAA and YARA-MK2i peptide mediated changes in extracellular surface charge of HCAVSMCs, *p < 0.05. **f** Schematic representation of the proposed mechanism of action for PPAA-mediated CPP-peptide uptake and intracellular delivery. Poly(propylacrylic acid) (PPAA) interacts with the cell membrane via hydrophobic interactions with the propyl moiety of PPAA. This interaction increases the net negative charge of the cell membrane due to the carboxylate anion present on the polymer. This increase in net negative charge enhances cell surface electrostatic interactions with cationic CPPs, leading to enhanced peptide uptake through both macropinocytic and endocytic pathways. Following uptake, the decrease in endosomal pH results in protonation of the PPAA polymer. This pH change triggers the polymer to dissociate from the CPP-peptide cargo and interact with and destabilize the endosomal membrane, resulting in cytosolic peptide delivery. Statistical analyses were performed with a one-way ANOVA followed by Tukey’s post hoc test. Data are presented as means ± SEM graphically

To clarify the mechanism of PPAA-mediated CPP delivery, we performed a series of polymer and peptide uptake experiments in the presence of a panel of inhibitors of various cellular internalization pathways (Table 2, Fig. 3a–c). In a study looking at uptake of fluorescent PPAA polymer (without peptide addition), it was observed that dynasore dose-dependently inhibited internalization (Fig. 3a). We then explored the effects of treatment with inhibitors during the polymer treatment phase only on subsequent YARA-MK2i peptide internalization (no inhibitors present during peptide treatment). Dynasore dose-dependent inhibition of PPAA uptake resulted in a corresponding dose-dependent increase in subsequent YARA-MK2i peptide internalization (Fig. 3b, c, Supplementary Fig. 12). In contrast, treatment with macropinocytosis inhibitor wortmannin during PPAA pretreatment significantly reduced subsequent peptide uptake. Importantly, the inhibitory effects of wortmannin are known to be longer lasting (exhibiting bioactivity over 48 h periods^22^) than dynasore (reversed within 20 min^23^) following removal from the media. Neither methyl-β-cyclodextrin nor dextran sulfate addition during PPAA pretreatment had any effects on subsequent peptide uptake. We then analyzed the effects of wortmannin and dynasore on peptide uptake (sequential and co-delivery) by pretreating the cells with inhibitors and leaving on the inhibitors throughout the duration of both PPAA and YARA-MK2i treatment. Both dynasore and wortmannin significantly inhibited YARA-MK2i peptide uptake, with wortmannin demonstrating a higher level of uptake inhibition (~69 and ~89% inhibition for co-delivery and sequential delivery, respectively) compared with dynasore (~30 and ~44% inhibition for co-delivery and sequential delivery, respectively) (Fig. 3d). We further verified that these effects are specific to inhibition of macropinocytosis and not a non-specific effect of wortmannin by utilizing EIPA and Cytochalasin D, which inhibit macropinocytosis through disparate mechanisms (Supplementary Fig. 13). We also extended the uptake inhibition experiments into macrophages to test a phagocytic cell type, which were similar for sequential delivery but more exclusively utilized phagocytosis for uptake with co-delivery (Supplementary Fig. 14).

**Table 2:** Uptake inhibitor overview

Inhibitor	Uptake pathway inhibited	Mechanism of action	Dose
Dynasore	Clathrin-mediated endocytosis	Dynamin inhibitor—prevents fission and internalization of clathrin coated vesicles	100 µM
Wortmannin	Macropinocytosis	Phosphatidylinositol-3-kinase (PI3K) inhibitor—PI3K is involved in a variety of actin-dependent processes associated with macropinocytosis	100 nM
5-(N-thyl-N-isopropyl) amiloride (EIPA)	Macropinocytosis	NA^+^/H^+^ exchange inhibitor—lowers submembranous pH and prevents Rac1 and Cdc42 signaling	50 µM
Cytochalasin D	Macropinocytosis	Binds to actin, altering and decreasing actin polymerization	50 µM
Methyl-β-cyclodextrin (MβCD)	Lipid raft-mediated endocytosis	Sequesters and depletes cholesterol from cell membranes	5 mM
Dextran sulfate	Scavenger receptor-mediated endocytosis	Competitively binds to scavenger receptors	100 µg/mL
Latrunculin A	Immunological/macrophage phagocytosis	Red sea sponge toxin that binds monomeric actin and prevents actin polymerization	50 µM

The uptake pathways affected by and underlying mechanism of action of the range of uptake inhibitors utilized

These data, particularly for sequential treatment, suggest a paradigm where PPAA effectively coats the cell membrane (potentially in an inhomogeneous manner), providing a polyanionic bait that helps to concentrate cationic peptide onto the cell surface where it is subsequently internalized by macropinocytosis and clathrin-mediated endocytosis. Furthermore, these data suggest that free PPAA polymer is internalized by clathrin-mediated endocytosis and that blocking this pathway during the polymer treatment helps to stabilize the polymer cell surface coating and consequently results in increased peptide uptake. In support of this model, we investigated the surface charge of cells treated with PPAA and YARA-MK2i. Cells were treated with polymer and/or peptide and repeatedly washed to remove any free, non-complexed PPAA or YARA-MK2i prior to analysis. Treatment with YARA-MK2i increased surface charge, whereas treatment with PPAA resulted in a dose-dependent decrease in cellular surface charge (Fig. 3e). Sequential treatment with PPAA followed by YARA-MK2i reversed polymer-mediated decreases in cell surface charge. These results further support a mechanism of action for PPAA-mediated enhancement of peptide uptake and intracellular delivery (Fig. 3f) where PPAA interacts with and coats the cell membrane, likely through hydrophobic interactions^24^. This coating effectively decreases the net negative charge of the cell membrane, which baits positively charged CPPs to the cell surface and induces cellular internalization. This mechanism is further supported by fluorescent microscopy demonstrating that the PPAA polymer rapidly associates with the cell membrane and that subsequent addition of the peptide results in immediate colocalization of the peptide with PPAA at the cell surface (Supplementary Figs. 15 and 16; Supplementary Movies 1–3). We verified that these findings were not influenced by the conjugation of fluorophores to the YARA-MK2i peptide or PPAA polymer (Supplementary Methods, Supplementary Fig. 17).

### PPAA endosomal disruption mechanism and intracellular fate

**Fig. 4:**
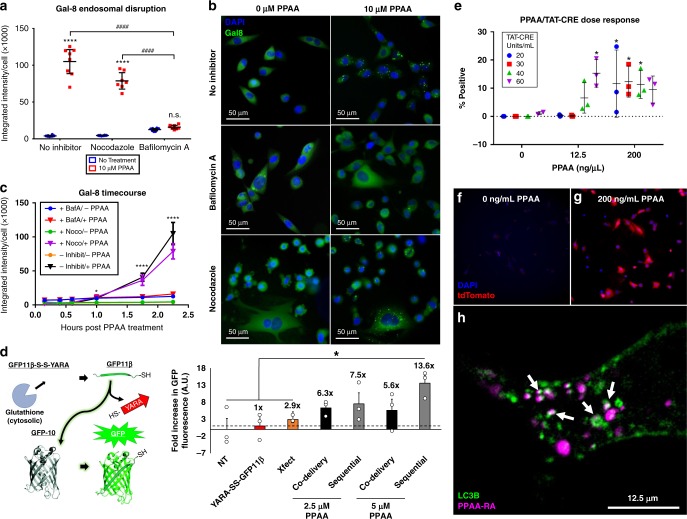
PPAA facilitates endosomal escape and is subsequently trafficked to autophagosomes. **a** Galectin-8 recruitment measured as the average Galectin-8 intensity per cell 2 h post-treatment with 0 or 10 µM PPAA in the presence or absence of the endosomal acidification inhibitor bafilomycin A and its control nocodazole. Data are presented as means ± 95% CI; two-way ANOVA followed by Tukey’s post hoc test. **b** Representative microscopy images of the treatment groups presented in A. Images are 712.8 × 712.8 µm. **c** Tracking in real-time indicates that PPAA causes significant (*p < 0.05) endosomal disruption compared with no treatment after 1 h which increases over the timeframe measured. Data are presented as means ± standard deviation. For **a**, **c**, *p < 0.05, ****p < 0.0001 vs. no inhibitor, ^####^*p* < 0.0001. **d** Comparison of delivery reagent-mediated intracellular peptide bioavailability through a quantitative split-GFP fluorescence transduction assay (numbers above bars denote the fold increase in GFP fluorescence compared with delivery of the peptide corresponding to the eleventh β-strand of the GFP protein alone); *p < 0.05; one-way ANOVA followed by Tukey’s post hoc test. Data are presented as means ± SEM. **e** PPAA dose-dependent intracellular delivery of TAT-CRE to Ai9 fibroblasts expressing a loxP flanked stop cassette upstream of a tdTomato transgene. Data are expressed as the percentage of treated cells positive for tdTomato expression as a measure of the intracellular bioavailability of the TAT-CRE cargo (12.5 ng/µL = 0.56 µM, 200 ng/µL = 9.1 µM PPAA). Two-way ANOVA testing of the data revealed a significant effect of PPAA dose on TAT-CRE mediated gene recombination (*p* < 0.0001). Tukey’s post hoc multiple comparisons testing for simple effects within each TAT-CRE dose revealed significant differences in gene recombination with increasing doses of PPAA; *p < 0.05. Data are presented as means ± standard deviation. Representative images of Ai9 fibroblasts pretreated with **f** 0 and **g** 200 ng/mL PPAA prior to treatment with 40 units/mL TAT-CRE. Images are 712.8 × 712.8 µm. **h** Representative still frame (from Supplementary Movie 4) demonstrating that LC3B (green) co-localizes (white arrows) with PPAA (purple) following cellular uptake

We then sought to further elucidate the mechanism of PPAA-mediated endosomal escape, its effects on the intracellular bioavailability of CPP-modified peptides and proteins, and the clearance and ultimate fate of PPAA following internalization. Figure 2c, d shows that PPAA helps peptide avoid endosome sequestration but this could conceivably be due to either PPAA-mediated endosome disruption or cell entry through a non-endosomal pathway (i.e., direct translocation). To address this question, we utilized a yellow fluorescent protein-Galectin-8 (YFP-Gal8) reporter cell line that we recently developed for direct in vitro visualization of endosome disruption^25^. Cells engineered with a YFP-Gal8 fusion protein display diffuse cytoplasmic fluorescence in the basal state and transition to a punctate appearance upon Gal8 binding and concentration onto disrupted endosomes^25^. In this experiment, we demonstrate that PPAA rapidly triggers endosome disruption. By utilizing the endosomal acidification inhibitor bafilomycin A and its control nocodazole, this study further verifies that the endosomal disruption activity of PPAA is driven by pH change in the endosome, which drives PPAA switch to a more hydrophobic, membrane-active confirmation (Fig. 4a–c).

Considering that the PPAA reagent resulted in significantly enhanced peptide uptake and endosomal escape, we hypothesized that it would significantly increase the intracellular bioavailability and concomitant bioactivity of peptides and proteins with cytosolic targets. To test this hypothesis, we performed a live cell split-GFP complementation transduction assay^18^. Green fluorescent protein (GFP) is a barrel structure composed of eleven β strands that allows for peptidyl backbone cyclization, which yields fluorescence^26^. This assay involves delivering the eleventh β strand of the GFP protein (GFP11β) to cells that have been transduced to stably express a non-fluorescent GFP fragment constituted by the first ten β strands of the GFP protein. This is a valuable assay for screening intracellular peptide delivery, as the GFP11β peptide requires endosomal escape to reach the GFP1-10 protein fragment inside the cell. We tested the ability of PPAA to deliver a GFP11β peptide conjugated to the YARA CPP (YARA-SS-GFP11β) through a reducible disulfide linkage that allows for intracellular reduction by glutathione to ensure that the YARA-CPP does not sterically hinder complexation with the GFP1-10 fragment (Fig. 4d). Co-delivery with 2.5 and 5 µM PPAA resulted in 6.3- and 5.6-fold increases in GFP fluorescence compared with delivery of the YARA-SS-GFP11β peptide alone, respectively. In contrast, sequential delivery with the YARA-SS-GFP11β peptide resulted in higher levels of GFP fluorescence than co-delivery (7.5- and 13.6-fold increases in fluorescence for 2.5 and 5 µM PPAA, respectively). Considering that the YARA-SS-GFP11β peptide is amphipathic (i.e., the GFP11β portion is primarily hydrophobic, see Supplementary Fig. 2), these results align with the previous observation that sequential delivery is optimal for amphipathic CPP delivery (Fig. 1). Notably, the closest competing reagent in terms of uptake and retention, Xfect, only produced a 2.9-fold increase in GFP fluorescence. To further assess PPAA-mediated enhancement of intracellular peptide bioavailability., we utilized the NanoLuc® Luciferase platform, which comprises a small HiBiT peptide modified with the YARA CPP that reconstitutes luciferase bioluminescence upon intracellular complexation with the Large BiT protein subunit. Sequential delivery of 5 µM PPAA followed by 0.5 or 1 µM YARA-HiBiT peptide significantly enhanced intracellular delivery (Supplementary Fig. 18), further demonstrating the PPAA potentiates intracellular delivery of CPP-modified peptide cargo.

We further explored the breadth of PPAA for delivery of the CRE recombinase protein modified with the CPP TAT (TAT-CRE) in an Ai9 reporter cell line. We generated an Ai9 fibroblast cell line by utilizing Cas9 RNPs to insert a linearized Ai9 plasmid containing a transcriptional termination signal (i.e., a STOP cassette) upstream of CAG promoter-driven red fluorescent protein variant (tdTomato) into the Rosa26 locus in NIH/3T3 mouse fibroblasts. This stop cassette can be removed by TAT-CRE-driven recombination, leading to activation of tdTomato expression^27^. At doses of 20, 30, and 40 units/mL TAT-CRE, pretreatment with PPAA resulted in a dose-dependent increase in TAT-CRE mediated tdTomato expression (Fig. 4e–g).

We next undertook a series of studies to elucidate the intracellular trafficking and ultimate fate of PPAA following cellular internalization. PPAA uptake kinetics reveal that the PPAA uptake begins after 5 min and increases linearly over time (Supplementary Fig. 19a). PPAA retention over time demonstrates that ~90% of the polymer is cleared from cells within 5 days, with an apparent elimination half-life of ~3 days (Supplementary Fig. 19b). Considering that PPAA facilitates endosomal escape and cytoplasmic delivery, we investigated if PPAA may potentially bind to mitochondria and concomitantly underlie the cytotoxicity observed at doses > 10 µM (Supplementary Fig. 20). Fluorescence microscopy of labeled PPAA uptake in conjunction with the mitochondrial stain MitoTracker Green demonstrated negligible colocalization with mitochondria (Supplementary Fig. 20a–e). Since Gal8 recruitment is associated with induction of macroautophagy, we hypothesized that following endosomal disruption PPAA may become sequestered in autophagosomes that fuse with lysosomes or are trafficked for exocytosis. In support of this hypothesis, fluorescence microscopy demonstrated robust colocalization of internalized PPAA with the autophagosomal marker LC3B (Fig. 4h). Time-lapse recordings further demonstrate that these areas of colocalization retain their association over time (Supplementary Movie 4).

### PPAA-mediated delivery of other cationic biologics

**Fig. 5:**
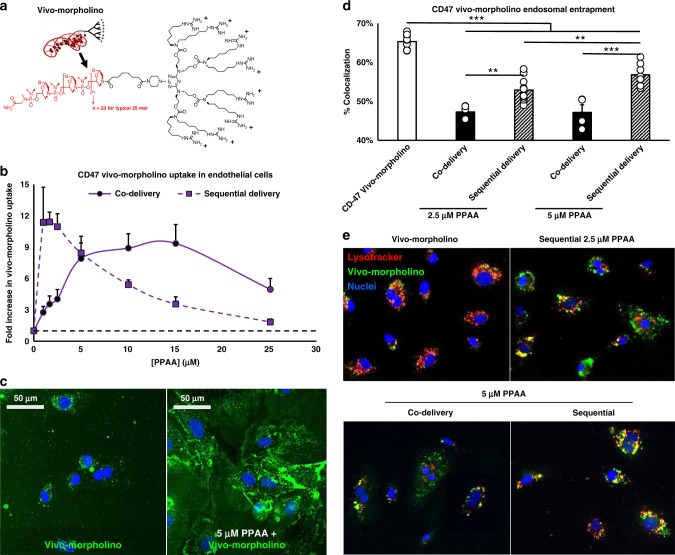
PPAA broadly enhances delivery of cationic biomacromolecules. **a** Structure of a vivo-morpholino constituted by phosphorodiamidite morpholino oligomer conjugated to a cationc octa-guanidine dendrimer. **b** Quantification and **c** representative confocal microscopy images of polymer dose-dependent CD47 vivo-morpholino uptake in human microvascular endothelial cells. **d** Quantification and **e** representative confocal microscopy images of vivo-morpholino (green) colocalization with the endolysosomal dye lysotracker (red). *p < 0.05, **p < 0.01, ***p < 0.001; one-way ANOVA followed by Tukey’s post hoc test. Data are presented as means ± SEM

To test if PPAA enhancement of cationic cargo delivery can be extrapolated to other, larger biomacromolecules beyond TAT-CRE (Fig. 4e–g), we investigated the PPAA dose-dependent uptake of a CD47 vivo-morpholino (phosphorodiamidite morpholino oligomer; MW ~25,000 g/mol). Morpholinos are synthetic nucleic acid analogs that exert antisense-like activity by binding to and sterically blocking complementary mRNA sequences. Vivo-morpholinos are morpholinos conjugated to a cationic, octa-guanidine dendrimer to facilitate cellular uptake (Fig. 5a). Co-delivery enhanced CD47 vivo-morpholino uptake ~10-fold in the dose range of 5–15 µM PPAA, whereas sequential delivery was found to enhance uptake ~12-fold in the lower dose range of 1–2.5 µM PPAA (Fig. 5b, c). We hypothesized that the pH-responsive membrane destabilizing activity of the PPAA polymer would also facilitate endosomal escape of the cytosolically active CD47 vivo-morpholino. Indeed, delivery with either 2.5 or 5 µM PPAA significantly reduced CD47 vivo-morpholino colocalization with the endolysosomal dye LysoTracker Red (Fig. 5d, e).

### PPAA-mediated delivery of cationic nanoparticles

**Fig. 6:**
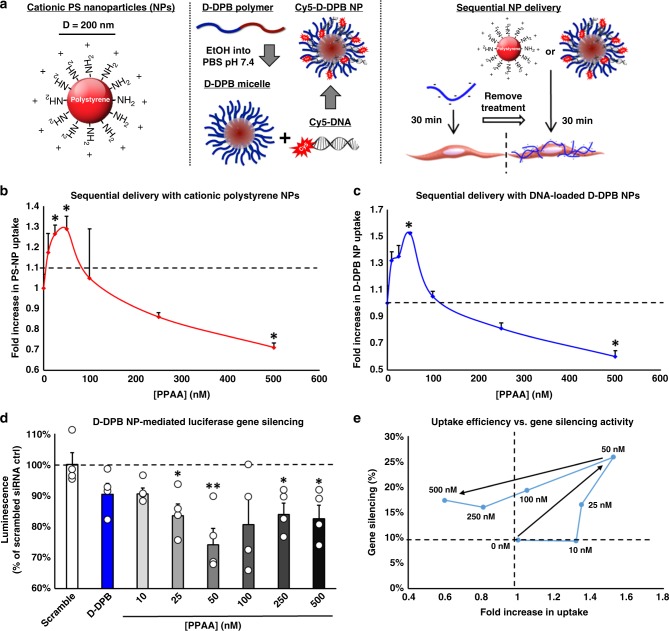
PPAA pretreatment enhances cationic nanoparticle uptake and bioactivity. **a** Schematic representation of cationic, amine-surface modified 200 nm polystyrene nanoparticles, synthesis of Cy5-labeled DNA-loaded D-DPB polymeric micellar nanoparticles, and sequential delivery of these nanoparticle formulations following PPAA pretreatment. The effects of dose-dependent PPAA pretreatment on the uptake of **b** cationic polysterene nanoparticles and **c** Cy5-labeled DNA-loaded D-DPB nanoparticles in A7r5 cells; *p < 0.05, vs. treatment with nanoparticles alone (i.e., 0 nM PPAA). **d** The effects of dose-dependent PPAA pretreatment on luciferase gene silencing in luciferase expressing A7r5s; *p < 0.05, **p < 0.01, vs. D-DPB nanoparticles loaded with scrambled siRNA (scramble). For **b**–**d**, statistical analyses were performed with a one-way ANOVA followed by a Tukey’s post hoc test. Data are presented as means ± SEM. **e** PPAA dose-dependent effects on uptake vs. bioactivity

To explore if PPAA can be extrapolated to enhance the delivery of cationic nanoparticles, we investigated the PPAA-mediated, dose-dependent uptake of cationic, fluorescent polystyrene nanoparticles (PS NPs, diameter = 200 nm, ζ-potential = + 29.1 mV) as well as polymeric (i.e., poly[DMAEMA_67_-b-(DMAEMA_29_-co-BMA_75_-co-PAA_40_)], or D-DPB)^28^, micellar nanoparticles (D-DPB NPs; diameter = 42 nm, ζ-potential = + 25.4 mV) loaded with fluorescently labeled DNA (Fig. 6a). We only investigated the effects of sequential delivery, since co-delivery with PPAA results in competing charge interactions that disrupt electrostatically complexed, DNA-loaded D-DPB nanoparticles. Due to the increased cationic charge density and size of these nanoparticle formulations compared with smaller cationic molecules, we investigated the effect of sequential delivery with PPAA across a lower dose range (10–500 nM), demonstrating that sequential delivery with 50 nM PPAA resulted in optimal uptake of both PS and D-DPB NPs (Fig. 6b, c). The ideal dose for potentiating nanoparticle delivery was in the dose range of 10–100 nM PPAA, significantly lower than smaller cationic molecules, possibly due to the increased charge density and size of these nanoparticle formulations relative to peptides proteins or morpholinos.

To investigate if PPAA-enhanced delivery of D-DPB NPs increases intracellular cargo bioavailability, we performed a luciferase gene-silencing assay. Sequential delivery of 50 nM PPAA for 30 min followed by treatment with D-DPB NPs loaded with luciferase siRNA for 30 min achieved optimal gene-silencing bioactivity (Fig. 6d). Comparing PPAA dose-dependent gene-silencing activity to PPAA-mediated enhancement of NP uptake shows an apparent association between uptake and gene silencing activity (Fig. 6e). Although sequential delivery with doses of PPAA > 100 nM did not result in significantly increased NP uptake, it did result in enhanced gene-silencing activity, likely due to bioavailability benefits driven by the endosome disruptive activity of PPAA. We further evaluated PPAA dose-dependent effects on luciferase gene-silencing activity of nano-polyplexes of siRNA formed with newer generation, secondary amphipathic CPPs PepFect and CADY, which show potential to effectively delivery nucleic acids without the cytotoxicity associated with other cationic vectors^29^ (Table 1, Supplementary Fig. 21). PPAA pretreatment enhanced the gene-silencing activity of sequentially delivered 25 nM siRNA delivered as both PepFect- and CADY-siRNA polyplexes; PPAA dose response trends for both of these polyplexes very closely mirrored that seen with D-DPB NPs with maximal gene silencing at 50 nM PPAA (Supplementary Fig. 21a, b).

### PPAA enhancement of CRISPR/Cas9-mediated gene editing

**Fig. 7:**
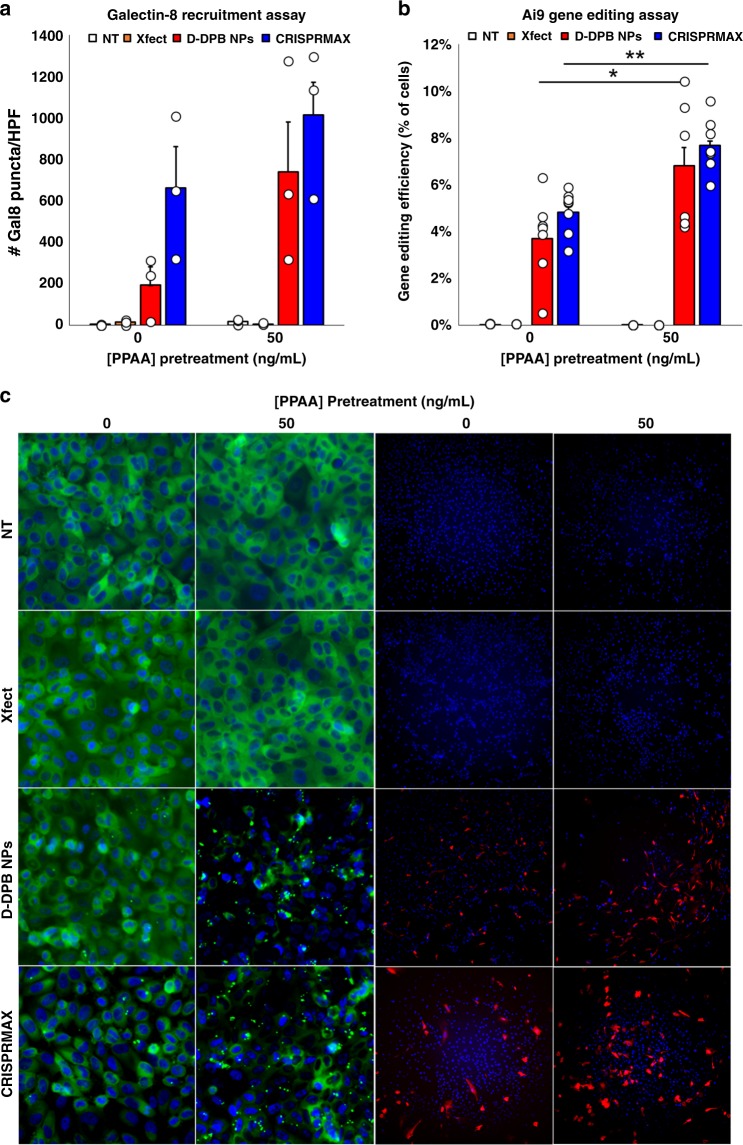
PPAA enhances endosomal escape and gene editing efficiency. Comparison of **a** Galectin-8 recruitment and **b** gene editing efficiency with various delivery systems with and without PPAA pretreatment (50 ng/mL); *p < 0.05. **p < 0.01; one-way ANOVA followed by a Tukey’s post hoc test. Data are presented as means ± SEM. Successful CRISPR/Cas9-mediated gene editing leads to removal of a termination cassette upstream of a tdTomato transgene in Ai9 fibroblasts, leading to activation of tdTomato expression. **c** Representative microscopy images of galectin-8-YFP recruitment to disrupted endosomes and tdTomato expression following CRISPR/Cas9-mediated gene editing in engineered Ai9 fibroblasts. All Gal8 micrographs shown are 458.6 × 458.6 µm; all Ai9 micrographs shown are 1404 × 1404 µm. 50 ng/mL = 2.27 nM PPAA

To explore if PPAA-mediated delivery can be extrapolated to enhance the delivery of the CRISPR/Cas9 RNPs for genome editing, we investigated the effects of sequential delivery of PPAA followed by Cas9 RNPs loaded into the protein delivery reagent Xfect, D-DPB NPs, and Lipofectamine^TM^ CRISPRMAX^TM^ lipid NPs in Gal8-MDA-MB-231 cells to assess endosomal disruption and an engineered Ai9 NIH/3T3 fibroblast cell line to assess gene editing efficiency (Fig. 7). Gal8 recruitment increased following PPAA pretreatment for CRISPR/Cas9 delivered with D-DPB NPs at doses of 25 and 50 ng/mL PPAA (Fig. 7a, c). Pretreatment with PPAA at doses ≥50 ng/mL was also found to increase Gal8 recruitment in cells treated with Lipofectamine CRISPRMAX^TM^, whereas untreated cells and cells treated with Xfect showed negligible Gal8 recruitment (Fig. 7a). In contrast to the data shown in Fig. 4a where a dose of 10 μM PPAA was utilized, treatment with 50 ng/mL (i.e., 2.3 nM PPAA, a 4400-fold lower dose compared with 10 μM PPAA) demonstrated no Gal8 recruitment. We then investigated the ability of PPAA pretreatment to enhance CRISPR/Cas9-mediated genome editing in the Ai9 reporter cell line. Gal8 recruitment was found to correlate with CRISPR/Cas9-mediated gene editing efficiency, where delivery of CRISPR/Cas9 with D-DPB NPs following pretreatment with 50 ng/mL PPAA and with CRISPRMAX^TM^ following pretreatment with ≥50 ng/mL PPAA significantly enhanced activation of tdTomato expression (Fig. 7b, c, Supplementary Fig. 22). In contrast, formulation of RNPs with Xfect demonstrated no detectable levels of gene editing.

## Discussion

We recently established the delivery efficiency of NPs comprising the anionic, pH-responsive polymer PPAA and a MAPKAP kinase 2 inhibitory peptide in clinical development for the prevention of fibrosis^30,31^. We optimized this initial formulation based on minimizing the size and polydispersity of the resulting electrostatically complexed nanoparticles. Interestingly, this work demonstrates that physicochemical properties of the polymer-peptide NPs did not correlate to enhanced peptide delivery (Supplementary Fig. 5). Rather, this work demonstrates that optimal peptide uptake is dependent on the concentration of polymer alone and was not a function of peptide dose or polymer to peptide mass/charge ratio (Supplementary Fig. 3). Consequently, we further characterized the PPAA dose-dependent delivery of a range of CPP-modified peptides, exploring both pre-formulated NPs and previously unexplored sequential polymer-peptide delivery.

Co-delivery with PPAA increased cell uptake with all cationic CPP-modified peptides (Fig. 1a); however, co-delivery with amphipathic CPP-modified peptides hindered uptake (Fig. 1b), likely because hydrophobic interactions between the peptide and PPAA competed with the hydrophobic interactions between the peptide and the cell membrane that drive uptake^32^. Cationic CPPs interact with the inherently anionic cell membrane primarily through electrostatic interactions with anionic lipids^9^ as well as charged proteoglycans such as heparan sulfate^33^. In contrast, amphipathic CPPs interact with the cell membrane, at least partially, through hydrophobic interactions^34^. Thus, we hypothesized that the optimal approach/conditions for use with the PPAA, which contains repeats of a pendant hydrophobic propyl moiety, may be different for this class of CPPs. Previous studies revealed that formulation of the YARA-MK2i peptide with PPAA induces membrane ruffling consistent with macropinocytosis^24^. Considering that the hydrophobic domain of amphipathic CPPs may also disrupt the interactions between PPAA and the cell membrane that drive macropinocytosis, we hypothesized that pre-coating cells with PPAA followed by subsequent delivery of the peptide may circumvent these competitive peptide-polymer interactions and optimally promote concentration of both components onto the cell surface. In line with this hypothesis, sequential delivery was found to be optimal for all amphipathic CPPs, producing increased uptake relative to the amphipathic CPPs alone (Fig. 1d).

We also found that sequential delivery works as a more generalizable approach for use of PPAA for all CPP classes, including purely cationic CPPs (Fig. 1c). We established that a cationic CPP segment is necessary for PPAA-mediated peptide uptake (Fig. 1f), and that PPAA promotes intracellular delivery across a wide range of cell types (Supplementary Figs. 6 and 7). Altogether, these results define the critical parameters needed to develop a generalized protocol that consistently works for a wide range of CPP-modified peptides across cell types. Specifically, sequential delivery of 2.5–5 µM PPAA followed by peptide (where the peptide dose is determined by the user dependent on the potency and independent of the dose of PPAA) represents an ideal approach.

In addition to defining critical treatment parameters, we established a mechanism of action for PPAA-mediated enhancement of peptide uptake whereby PPAA heterogeneously coats the cell membrane and serves to electrostatically concentrate and facilitate intracellular delivery of cationic cargo (Fig. 3). This process approximates layer-by-layer (LBL) assembly, which has been used to create functional cell surface coatings through sequential adsorption of oppositely charged components that form thin polyelectrolyte multilayer films. This approach has been explored primarily in the modification of pancreatic islets^35–37^ to dampen host immune reactivity and not for the intracellular delivery of therapeutics. Furthermore, previous LBL approaches have almost entirely utilized polycations to modify negatively charged cell surfaces, which is associated with significant cytotoxicity^38^. To our knowledge, this is the first cell surface modification approach that utilizes an anionic polymer to modify cell surfaces to mediate cationic therapeutic intracellular delivery.

This approach also benefits from the endosome escape function of PPAA, which transitions from a predominantly deprotonated, expanded state into an uncharged, globular conformation that disrupts lipid bilayers at acidic endosomal pH^15^. Furthermore, PPAA protonation in acidic environments reduces its ionization and facilitates a loss of electrostatic interactions with cationic cargo, enabling intracellular release. This concept is supported by the comparative endosomal escape (Fig. 2c, d), Galectin-8 (Fig. 4a–c), and intracellular bioavailability assays (Fig. 4d–g) where it was found that PPAA mediates pH-dependent endosomal escape and increased peptide and protein intracellular bioavailability. The key differentiating factor is that PPAA is an anionic, pH-responsive polymer that mediates interactions with cell membranes through hydrophobic interactions, whereas polycationic reagents mediate interaction with cell membranes through electrostatic interactions. Other non-commercially available approaches to peptide and protein delivery focus on the use of cell penetrating peptides/protein transduction domains that are typically cationic (also driving internalization through electrostatic interactions with cellular membranes) and often suffer from endolysosomal entrapment^18,21,39–42^.

In addition to peptides, we demonstrate that PPAA can be extrapolated as an intracellular delivery reagent for other, larger cationized biologics (Figs. 4e–g, 5), and cationic nanoparticles delivering siRNA (Fig. 6, S21) and CRISPR/Cas9 RNPs (Fig. 7, S22). We sought to test sequential delivery of PPAA followed by pre-formed nucleic acid complexes without requiring any additional re-formulation or synthesis steps, an approach that is unique to prior work where PPAA was incorporated as a formulation component of cationic lipid complexes to enhance uptake and gene-silencing activity of antisense oligonucleotides^43^. Despite the excitement around the use of nucleic acid-based RNA interference and gene editing^44^, delivery remains the major challenge. Delivery systems for gene editing therapies include cationic lipids such as lipofectamine, liposomes, cationic polymers such as branched PEI, cationic gold based hybrid nanoparticles, and the use of cell penetrating peptides^45,46^. For example, a recent work utilizing a gold nanoparticle conjugated to cationic polymers for RNP delivery achieved 11.3% efficiency in editing blue fluorescent protein expressing HEK293 cells to express GFP via homology directed repair^47^. Another recent report detailed the use of Lipofectamine CRISPRMAX to achieve 6.5% restoration of GFP fluorescence in HEK293 cells harboring a disrupted EmGFP gene with delivery of dsDNA^48^. Our work demonstrates that PPAA pretreatment has the potential to enhance the efficacy of these approaches: pretreatment with PPAA significantly enhances cationic D-DPB NP delivery as well as CRISPRMAX mediated delivery of RNPs for gene editing, increasing editing efficiency from 4.8 to 9% in engineered Ai9 fibroblasts. Approaches to enhance gene editing efficiency with delivery reagents have focused almost entirely on optimizing experimental design (i.e., cell cycle synchronization, optimizing sgRNA sequence and structure, maintaining cells in log phase growth, etc.^49^). In sum, the use of PPAA as a pretreatment to facilitate the uptake of biologics represents a novel, simple approach to facilitate the intracellular delivery of cationic peptides, biomacromolecules, and nanoparticles/delivery reagents for gene therapy and gene editing.

## Methods

### Materials

YARA-MK2i (YARAAARQARA-KALARQLGVAA)^4^, TAT-MK2i (GRKKRRQRRRPPQ-KALARQLGVAA), R6-MK2i (RRRRRR-KALARQLGVAA), Penetratin-MK2i (RQIKIWFQNRRMKWKK-KALARQLGVAA), Transportan-MK2i (GWTLNSAGYLLGKINLKALAALAKKIL-KALARQLGVAA), YARA-VASP (YARAAARQARA-KLRKVS_p_K; where S_P_ denotes a phosphorylated Serine residue), and VASP (KLRKVSpK) peptides were synthesized by and purchased from EZBioLab (Carmel, IN) at a scale of 500 mg with a purity ≥95% as determined by mass spectrometry. The 11 amino acid HiBiT sequence was obtained free of charge from Promega under a limited use license, and was synthesized as N-terminal fusions to the YARA CPP (YARAAARQARA-HiBiT) by EZBiolab at 20 mg scale to >95% purity. The DrkBiT inhibitor peptide (VSGWALFKKIS) was synthesized by GenScript’s Fast Peptide Synthesis service at 19 mg scale and >95% purity. CADY (Ac-GLWRALWRLLRSLWRLLWRA-cya) peptide was purchased from EZBioLab at a scale of 20 mg with a purity ≥80% as determined by mass spectrometry. PepFect (Stearyl-AGYLLGKLLOOLAAAALOOLL) was purchased from PepFect (Stockholm, Sweden). Alexa Fluor™ 488 N-hydroxysuccinimidyl ester (Alexa-488-NHS), LysoTracker Red DND-99, MitoTracker Green, CellTracker Green, and Pierce Protein Transfection Reagent (Pro-Ject) were purchased from ThermoFisher Scientific. PULSin was purchased from VWR. ProteoJuice was purchased from EMD Millipore. BioPorter was purchased from Genlantis. Profect-1 and Profect-2 were purchased from Targeting Systems. Xfect was purchased from Clontech. Fluorescein labeled CD47 vivo-morpholino (5′-CGTCACAGGCAGGACCCACTGCCCA) was purchased from Gene Tools, LLC. Amine-modified 200 nm diameter red fluorescent Fluospheres (Ex/Em = 580/605) were purchased from ThermoFisher Scientific. The pGreenFire1-mCMV (EF1α-puro) luciferase plasmid (pTRH1 mCMV dscGFP T2A Fluc) was purchased from System Biosciences. All other plasmids were purchased from Addgene. Lipofectamine^TM^ CRISPRMAX^TM^ Cas9 transfection reagent was purchased from ThermoFisher Scientific. Cas9 protein was prepared as described below for Ai9 cell line creation. For gene-editing assays TrueCut Cas9 Protein V2 was purchased from ThermoFisher. For cell line creation, sgRNAs were generated by in vitro transcription as described below in in vitro sgRNA preparation. For gene-editing assays, Ai9-L and Ai9-R sgRNAs with 2-O-methyl 3′ phosphorothioate modifications in the first and last 3 nucleotides were purchased from Synthego (Supplementary Table 2). TAT-CRE was purchased from EMD Millipore. All other materials were purchased from Sigma Aldrich unless otherwise noted.

### Monomer synthesis

The propylacrylic acid monomer (PAA) was synthesized using propylmalonic acid diethyl ester as a precursor^50^. Briefly, 0.53 mol diethyl n-propylmalonate (Alfa Aesar) was added to a round bottom flask and stirred overnight with 700 mL of 1 M KOH in 95% ethanol. Following rotary evaporation, the resulting residue was re-solubilized in distilled water and acidified with slow addition of concentrated hydrochloric acid until a pH of 2.0 was achieved. The mixture was added to a separatory funnel and 2-(propoxycarbonyl) butanoic acid was extracted three times. The organic extract was dried using magnesium sulfate, and the diethyl ether was removed by rotary evaporation to obtain crude 2-carbopropoxybutyric acid that was subsequently cooled to −5 °C. Diethylamine (55 mL, 0.53 mol) and subsequently 43.5 g of formalin solution (0.54 mol) were added while stirring. The mixture was warmed to room temperature, stirred for 24 h, and subsequently outfitted with a reflux condenser and refluxed for 8 h at 60 °C. The refluxed mixture was cooled to 0 °C and concentrated sulfuric acid was added until no more gas was produced. The resulting reaction mixture was added to a separatory funnel and 2-propylacrylate was extracted three time with diethyl ether, dried over magnesium sulfate. Following rotoary evaporation of the diethyl ether, 2-propylacrylate was then mixed with 175 mL of 1 M KOH and refluxed for 20 h at 120 °C. After cooling to room temperature, dilute (1 M) hydrochloric acid was added to the solution until a pH of 2 was achieved and an oil layer was formed. In a separatory funnel, the oil was extracted four times with diethyl ether and dried over magnesium sulfate. The diethyl ether was removed on a rotary evaporator to yield a yellow oil that was distilled via short path distillation to yield colorless, pure PAA (Yield ~35%).

### Polymer synthesis

The chain transfer agent (CTA) 4-Cyano-4-(ethylsulfanylthiocarbonyl) sulfanylpentanoic acid (ECT) was utilized in all RAFT polymerizations^51^. Briefly, ethanethiol (4.72 g, 76 mmol) was added slowly to sodium hydride (60% in oil) (3.15 g, 79 mmol) in diethyl ether (150 ml) at 0 °C. After ten minutes of stirring, carbon disulfide (6.0 g, 79 mmol) was added. The mixture was filtered to obtain sodium S-ethyl trithiocarbonate (7.85 g, 0.049 mol), which was subsequently resuspended in diethyl ether and reacted with 6.3 g (0.025 mol) iodine for 1 h. Crude bis(ethylsulfanylthiocarbonyl) disulfide (1.37 g, 0.005 mol) was isolated from the mixture by filtration, washing with sodium thiosulfate, and drying with sodium sulfate. The remaining ether was removed by rotary evaporation and the crude bis(ethylsulfanylthiocarbonyl) disulfide was resuspended in 50 mL of ethyl acetate to which 2.1 g (0.0075 mol) 4,4′-azobis(4-cyanopentanoic acid) was added. The mixture was refluxed for 18 h at 80 °C. Ethyl acetate was subsequently removed on a rotary evaporator and ECT was purified via silica gel column chromatography using 50:50 ethyl acetate:hexane. ^1^H NMR (CDCl_3_) δ 1.36 t (SCH_2_CH_3_); δ 1.88 s (CCNCH_3_); δ 2.3–2.65 m (CH_2_CH_2_); δ 3.35 q (SCH_2_CH_3_).

For the RAFT polymerization of PPAA, 2,2′-azo-bis-isobutyrylnitrile (AIBN) and ECT were added in a 1:1 molar ratio to the propylacrylic acid monomer in a pear shaped flask. The monomer:CTA ratio was set so that a target molecular weight of 22,500 g/mol would be achieved at full conversion. The reaction mix was freeze-vacuum-thawed three times and subsequently purged with ultra-pure nitrogen for 30 min prior to submersion in an oil bath at 70 °C to initiate polymerization. The polymerization was carried out for 48 h and the resulting polymer was dissolved in DMF and precipitated into cold diethyl ether five times prior to drying overnight under vacuum. Fluorescent PPAA was synthesized by the same method by including 0.5 mol% rhodamine B acrylate (Polysciences, Inc.) in the polymerization to yield poly(propylacrylic acid-co-rhodamine acrylate) (PPAA-RA). PPAA and PPAA-RA were purified by dialysis against methanol at 4 °C utilizing a 6000–8000 MWCO dialysis Membrane (Spectra/Por). The diblock terpolymer Poly[DMAEMA_67_-b-(DMAEMA_29_-co-BMA_75_-co-PAA_40_)] (i.e., D-DPB) was synthesized by RAFT polymerization of a 2-(dimethylamino) ethyl methacrylate (DMAEMA) macro CTA from ECT from which the second terpolymer block containing DMAEMA, 2-propylacrylic acid (PAA), and butyl methacrylate (BMA). Gel permeation chromatography was used to determine molecular weight and polydispersity of the PPAA homopolymer and PPAA-RA copolymer using HPLC-grade DMF containing 0.1% LiBr at 60 °C as the mobile phase. Molecular weight calculations were performed with ASTRA V software and were based on experimentally-determined d*η*/d*C* values determined through offline injections of serial dilutions of each polymer through a refractive index detector [calculated PPAA d*η*/d*C* = 0.087 mL/g, *M*_n_ = 22,292 g/mol, PDI = 1.47; calculated PPAA-RA d*η*/d*C* = 0.089 mL/g, *M*_n_ = 20,860 g/mol, PDI = 1.241; calculated D-DPB d*η*/d*C* = 0.049 mL/g, *M*_n_ = 30,500 g/mol, PDI = 1.108]. Polymer purity, compostion, and molecular weights were then verified through ^1^H NMR spectroscopy utilizing DMSO-d_6_ as a solvent for PPAA and PPAA-RA homo- and co-polymers and CDCl_3_ as a solvent for the D-DPB terpolymer.

### YARA-SS-GFP11β peptide synthesis and purification

A peptide corresponding to the 11th β-strand of green fluorescent protein (GFP11β peptide—TIGAANVYEHLVMHDR) was received from EZBioLab. YARA-SH (YARAAAARQARAC) was synthesized on a rink amide resin utilizing standard Fmoc chemistry with a PS3 peptide synthesizer (Gyros Protein Technologies). The YARA-SH peptide was then cleaved/deprotected in TFA/Phenol/H_2_O/tri-isopropylsilane (88/5/5/2), precipitated into ether, and dried in vacuo. GPF11β was dissolved in PBS containing 1 mM EDTA and 0.02% sodium azide at a final concentration of 5 mg/mL and pH 7.5. A 5-fold molar excess of succinimidyl 3-(2-pyridyldithio) propionate (SPDP) was dissolved in minimal DMF, immediately transferred to the GFP11β solution, and stirred for 4 h at room temperature. The product was purified on a Waters 1525 binary pump HPLC with Waters 2489 UV detector and Agilent Zorbax C18 column. The mobile phase consisted of H_2_O + 0.1% TFA as solvent A, and acetonitrile + 0.1% TFA as solvent B. Peptide was eluted over a 10 min linear gradient of 95:5 to 50:50 A to B, followed by a 5 min linear gradient of 50:50 to 5:95 A to B to elute excess SPDP. The purified pyridyl disulfide functionalized GFP11β peptide was dissolved in the previously described buffer at 5 mg/mL, a threefold molar excess of YARA-SH was added, and the reaction mixture was stirred for 24 h at room temperature. Reaction completion was verified by monitoring 2-pyridinethione release via UV absorbance at 343 nm on a Varian 50 Bio UV/Vis spectrometer. GFP11β-SS-YARA was purified using HPLC as described above, and the molecular weight was confirmed using electron spray ionization-mass spectroscopy (ESI-MS) on a Waters Synapt in positive ion mode (GPF11β-SS-YARA predicted: 3219.7 amu, found: 1610.4 amu (m/2), 1073.6 amu (m/3), 805.5 amu (m/4).

### In vitro sgRNA preparation

PCR amplified sgRNA template sequences were obtained using a plasmid donor along with forward primers containing T7 promoter, the desired guide target sequence, and an invariant sequence and a universal reverse primer (Supplementary Table 2). The sgRNA template amplicons were then purified by gel extraction (Qiagen) following manufacturer’s guidelines. In vitro transcription of sgRNA was then carried out following the MEGAshortscript T7 (Invitrogen) transcription protocol. TRIzol (Invitrogen) extraction of sgRNA was then carried out following manufacturer’s guidelines, except that 1 ml of TRIzol was added per 80 μl of T7 transcription product. The top sgRNA containing aqueous phase was then transferred to a fresh tube and sgRNA was precipitated using isopropanol, pelleted, and washed with 75% ethanol. The RNA pellet was then air dried in a culture hood followed by resuspension and concentrated in RNase free water. Aliquots were then frozen and stored at −80 °C.

### Cas9 protein synthesis and purification

Rosetta 2 cells (Millipore EMD) containing chloramphenicol resistant plasmid encoding for mammalian tRNAs were transformed with pET28b(+)-SpCas9-His (kanamycin resistant). Transformed cells were plated on LB agar containing 50 μg/mL kanamycin and 30 μg/mL chloramphenicol. Colonies were selected and grown in a starter culture overnight. The following morning, 10 mL from the starter culture was spiked into 1 L LB containing antibiotics and allowed to grow at 37 °C until OD600 reaches 0.6–0.8. The temperature was then reduced to 18 °C and spCas9 synthesis was carried out overnight following induction with 0.2 mM IPTG. The cells were then isolated and resuspended in 6 mL/g in lysis buffer containing EDTA-free protease inhibitor cocktail (Roche), 1 mM PMSF, and 1 mg/mL lysozyme (sigma), with the remainder of the protocol carried out at 4 °C. Cells were sonicated 10 s on and 10 s off for 15 min. The cell debris was then pelleted by centrifugation 30 min at 16,000 × *g* at 4 °C. Ni-NTA agarose beads (Qiagen) were then added to the collected supernatant for isolation of his-tagged protein, following manufacturer’s guidelines. Contaminating DNA was removed from the his-tag purified eluate using Sepharose (Sigma), collecting the protein flow through. The protein was dialyzed at 4 °C overnight using 10 kDa MWCO dialysis tubing, and then concentrated with Vivaspin 20 50 kDa MWCO spin filters (GE Healthcare). Samples were then sterile filtered, aliquoted, flash frozen, and stored at −80 °C. SpCas9 Protein was analyzed by Coomassie staining and western blot (Supplementary Fig. 23).

### Fluorescent labeling of peptides

Alexa-488-NHS was dissolved in 800 µL DMSO and mixed at a 1:3 molar ratio with each peptide (with the exception of the YARA-SS-GFP11β peptide) in 200 µL of 100 mM sodium bicarbonate buffer (pH = 8.3) and allowed to react for 4 h while protected from light. Unreacted fluorophore, N-hydroxysuccinimide salts, and organic solvent were removed using a PD-10 miditrap G-10 desalting column, and the purified, fluorescently labeled peptides were lyophilized and stored until reconstitution for use in experiments.

### Cell culture

Primary human coronary artery vascular smooth muscle cells (HCAVSMCs) and human microvascular endothelial cells (HMVECs) were obtained from Lonza. Murine leukemic monocyte macrophages (RAW 264.7), human mammary gland epithelial breast cancer cells (MCF7), human embryonic kidney cells (HEK 293T), rat aortic smooth muscle cells (A7r5), and NIH/3T3 Mouse Embryonic Fibroblasts were purchased from American Type Culture Collection (ATCC). HCAVSMCs were cultured in complete growth medium [vascular cell basal medium (ATCC) supplemented with 5% FBS, human basic fibroblast growth factor (bFGF, 5 ng/mL), human insulin (5 µg/mL), ascorbic acid (50 µg/mL), L-glutamine (10 mM), and human epidermal growth factor (EGF, 5 ng/mL)]. HMVECs were grown in EGM-2 medium supplemented with an EGM-2 bullet kit (Lonza). RAW 264.7, MCF7, HEK293T, A7r5, and NIH/3T3 cells were grown in DMEM supplemented with 10% FBS. All media were supplemented with 1% penicillin-streptomycin (P/S) and 50 µg/mL plasmocin (Invivogen). All cell cultures were maintained in a sterile incubator maintained at 37 °C with a humidified, 5% CO_2_ atmosphere.

Cell cultures were maintained on a 75 cm^2^ polystyrene tissue culture flasks (T75; BD Falcon) and culture media was replaced every other day. Cells were seeded at densities specific to each experiment, and, prior to harvest and passage, cells were grown to 80–90% confluence. Only cells from early passages (i.e., 3–8) were used in experiments.

### LgBiT plasmid construction

The plasmid PB EF1A LgBiT CMV eGFP/Bsd was designed in silico and constructed by VectorBuilder.com. Briefly, the plasmid contains the following elements after the 5′ inverted terminal repeat (5′ ITR): the human eukaryotic translation elongation factor 1 alpha1 promoter (EF1A) promoter, the LgBiT fragment sequence, a stop codon, and the rabbit beta-globin polyadenylation (rBG pA) signal, followed by a cytomegalovirus (CMV) promoter driving expression of enhanced green fluorescent protein and blasticidin deaminase (EGFP/Bsd) fusion protein that served as a dual fluorescence/antibiotic resistance selection marker, a stop codon, and the bovine growth hormone polyadenylation signal (BGH pA) terminated by the 3′ ITR. The LgBiT sequence was made available free of charge to the authors by Promega under a limited use license.

### Generation of stably expressing GFP1-10 cells

For viral production, HEK293T cells were plated in a T75 flask and grown to ~50% confluence. VSV-G-expressing envelope plasmid pCMV-VSV-G (1 µg), psPAX2 packaging plasmid (10 µg), and the lentiviral vector for the split-GFP protein GFP1-10 pCJMGFP1-10 (10 µg) were transfected into cells utilizing FuGENE® 6 transfection reagent (Promega) in DMEM with 5% FBS (no antibiotics). Twenty-four hours later, the treatment was removed and replaced with fresh medium. Fifty-six hours later, the supernatant was collected, centrifuged at 2000 × *g* for 10 min, and syringe filtered through a 0.45 µm PTFE filter. Viral supernatant was stored at −80 °C until further use. A7r5 cells stably expressing GFP1-10 were generated by transduction with lentivirus diluted 1:10 in DMEM with 10% FBS and 8 (µg/mL) polybrene infection reagent (EMD Millipore) for 24 h. Transduced cells were expanded, harvested, and stored in cryovials with freezing medium (10% DMSO in FBS) in a liquid nitrogen cell cryotank until further use.

### Generation of stably expressing luciferase cells

For viral production, HEK293T cells were plated in a T75 flask and grown to ~50% confluence. VSV-G-expressing envelope plasmid pCMV-VSV-G (1 µg), psPAX2 packaging plasmid (10 µg), and pGreenFire1-mCMV (EF1α-puro) luciferase plasmid (10 µg) were transfected into cells utilizing FuGENE® 6 transfection reagent (Promega) in DMEM with 5% FBS (no antibiotics). Twenty-four hours later, the treatment was removed and replaced with fresh medium. Fifty-six hours later, the supernatant was collected, centrifuged at 2000 × *g* for 10 min, and syringe filtered through a 0.45 µm PTFE filter. Viral supernatant was stored at −80 °C until further use. A7r5 cells stably expressing luciferase were generated by transduction with lentivirus diluted 1:2 in DMEM with 10% FBS and 8 (µg/mL) polybrene infection reagent (EMD Millipore) for 24 h. During the first 30 min of viral transduction cells were spinoculated at 2000 rpm and then subsequently transferred to a cell culture incubator. After 24 h, viral treatments were removed, and the cells incubated in fresh medium for an additional 24 h. Cells were then selected over a 10-day period with escalating doses of puromycin (2.5–15 µg/mL). Selected cells were then harvested and stored in cryovials with freezing medium (10% DMSO in FBS) in a liquid nitrogen cell cryotank until further use.

### Generation of stably expressing LgBiT cells

HEK-293-T LgBiT cells were generated using the PiggyBac transposase system to enable stable integration of transgene constructs. Cells were plated at 10^5^ cells per well in a 6-well dish and allowed to adhere overnight. Cells were then co-transfected with 2 μg each of PB EF1A LgBiT CMV eGFP/Bsd and PiggyBac Transposase expression vectors using Lipofectamine 2000 according to manufacturer protocol. After 24 h, cells were selected using 5 μg/mL blasticidin for two weeks. Cells were periodically monitored for eGFP expression by fluorescence microscopy.

### Generation of stably expressing YFP-Galectin-8 cells

Gal8 retrovirus was generated using HEK 293-T cell transfected with Gal8-YFP, pUMVC, and pCMV-VSV-G. Gal8-YFP transduced cells (Gal8-MDA-MB-231) were obtained by selection with blasticidin for one week followed by single clonal expansions obtained through the limiting dilution method in blasticidin containing media; clonal populations were used to ensure consistent expression of YFP constructs^25^.

### Generation of stably expressing LC3B-mTurquoise2 cells

Pseudotyped retrovirus was generated using the transfer plasmid pBABEpuro mTurquoise2 LC3B (Addgene #78518). This plasmid was co-transfected along with packaging plasmids pMD2.G (Plasmid #12259) and pCMV-dR8.2 dvpr (Plasmid #8455) into HEK 293-T cells with lipofectamine 2000 according to manufacturer protocol. Viral supernatant was harvested, filtered through a 0.45 μm syringe filter, and concentrated ~20 × using Amicon 15 mL 30 kDa MWCO spin filters. Virus was aliquoted and stored at −80 °C until use. LC3B-mTurquoise HEK-293-T cells were generated using using frozen retrovirus. HEK293-T cells were plated at 10,000 cells per well in a 96-well plate and subjected to a 12 point threefold dilution series of concentrated retrovirus in antibiotic free DMEM supplemented with 10% FBS. The virus was applied 24 h then exchanged with fresh media. Cells were then passaged into 6-well plates containing selection media (DMEM supplemented to 5 μg/mL puromycin and 10% FBS). Cells were periodically monitored by phase contrast and fluorescence microscopy. When any well reached confluence, all cell populations were moved to a larger well plate (first 24, then 12, then 6-well plates). The population which received the highest concentration of retrovirus which displayed normal morphology (population 2) was expanded in selection media, aliquotted, and stored in the vapor phase of a liquid nitrogen cryotank until further use.

### Generation of Ai9 cells

Ai9 plasmid (Addgene 22799, encoding Rosa-CAG-LSL-tdTomato-WPRE containing a flanked STOP cassette to prevent transcription of the red fluorescent protein variant tdTomato) was amplified in Stbl3 *E. coli* in an overnight liquid culture and purified with a GenElute HP Endotoxin-Free Plasmid Maxiprep kit. Lipofectamine CRISPRMAX and Lipofectamine 3000 were used to simultaneously transfect the Cas9 ribonucleoproteins (RNPs) and Ai9 plasmid, respectively, into NIH/3T3 murine fibroblasts according to manufacturer specifications. The Cas9 RNPs were targeted to cut the plasmid into two linearized pieces at the Rosa26 left arm and Rosa26 right arm regions with the RosaRA and RosaLA single guide RNAs generated by in vitro transcription. These same sequences also target the Rosa26 locus of the NIH/3T3s to cause a double stranded break in the genome. The homology of the plasmid with these loci facilitates genomic integration of the plasmid sequence. Prior to editing and genomic integration, the Ai9 plasmid encodes Diptheria Toxin A for selection of cells that do not integrate the linearized Ai9 construct.

Forty-eight hours after transfection, cells were selected over a 30-day period with escalating doses of G418 (0.5–1.75 mg/mL). Selected cells were then harvested and stored in cryovials with freezing medium (10% DMSO in FBS) in a liquid nitrogen cell cryotank. Cells were subsequently expanded in media containing 1 mg/mL G418 until further use.

### Preparation of polymer and peptide formulations

PPAA was added to phosphate buffered saline (PBS, without calcium or magnesium), and 1 M NaOH was slowly added until the polymer was completely solubilized and a stable pH of 8 was obtained. Unlabeled and fluorescently labeled peptides were dissolved in pH 8 phosphate buffered saline without calcium or magnesium. A pH of 8 was chosen as it is between the acid dissociation constants (i.e., pK_a_) of the carboxylic acid side chains present on the PPAA polymer (pK_a_ ~ 6.7) and the primary amines present in amino acid side chains (pK_a_ > 9). This pH ensures that the PPAA polymer is predominantly deprotonated and the peptide is predominantly protonated to facilitate electrostatic interactions. For co-delivery treatments, appropriate volumes of PPAA and peptide stock solutions were mixed to achieve mass ratios ranging from 5:1 to 1:20 (peptide:PPAA) resulting in the formation of electrostatically complexed nanoparticles, or nano-polyplexes (NPs).

### Assembly and characterization of DNA- and siRNA-loaded D-DPB polymeric nanoparticles (D-DPB NPs)

For the formation of D-DPB NPs, poly[DMAEMA_67_-b-(DMAEMA_29_-co-BMA_75_-co-PAA_40_)] (D-DPB) was dissolved in a minimal volume of ethanol (20 mg/mL) in a 2 mL RNAse free polypropylene tube followed by slow addition of deionized water to trigger spontaneous micelle formation (final polymer concentration 1 mg/mL). Micellar size, polydispersity, and ζ-potential were characterized by Dynamic Light Scattering (DLS, Zetasizer nano-ZS Malvern Instruments Ltd). To form DNA or siRNA-loaded D-DPB NPs, 25 nM of fluorescent Cyanine-5 labeled DNA (Cy5-DNA), luciferase siRNA, or scrambled control siRNA and a calculated amount of NP stock solution to achieve an N/P ratio of 4 were mixed in PBS (−/−) and allowed to electrostatically complex for 30 min.

### Flow cytometry

Depending on the experiment, HCAVSMCs, HMVECs, RAW 264.7, MCF7, HEK293T, or A7r5 cells were grown to 80–90% confluence, harvested, and seeded at 60,000 cells/well in a 12-well plate and allowed to adhere overnight. Cells were treated as defined in the following sections in Opti-MEM medium supplemented with 1% penicillin-streptomycin and 1% FBS. For all flow cytometry assays, following treatment or post-treatment incubation, cells were washed 2× in PBS without calcium or magnesium, harvested with 0.05% trypsin-EDTA, centrifuged, resuspended in 300 µL of 0.05% Trypan blue in PBS without calcium or magnesium, and transferred to a 96-well plate for analysis on an EMD Millipore Guava easyCyte™ 5HT flow cytometer with InCyte software for data acquisition. Data were exported and analyzed with FlowJo software (V 10.1). All samples were run in triplicate. All flow cytometric uptake data are presented as fold increase in peptide, vivo-morpholino, or nanoparticle uptake relative to treatment with an equivalent dose of the corresponding fluorescently labeled peptide, vivo-morpholino, or nanoparticle formulation alone. Absolute fluorescence data of peptide uptake is shown in the supplement (Supplementary Figs. 4 and 7).

### Co-delivery with PPAA

PPAA polymer was mixed with fluorescently labeled peptides or fluorescently labeled CD47 vivo-morpholino at mass ratios ranging from 3:1 to 1:20 (peptide:polymer) and 5:1 to 1:10 (vivo-morpholino:polymer), respectively. A concentration of 5 µM Alexa-488 labeled peptide or fluorescein labeled CD47 vivo-morpholino use utilized in all formulations. Cells were then treated with each formulation for 30 min. For investigating the influence of peptide dose on PPAA-mediated uptake, The same range of mass ratios [i.e., 3:1 to 1:20 (peptide:polymer)] was utilized for doses of 5, 10, and 25 µM fluorescently labeled YARA-MK2i peptide.

### Sequential delivery with PPAA

Separate PPAA and fluorescently labeled peptide and vivo-morpholino stocks corresponding to the same respective doses present in the range of mass ratios for co-delivery were prepared. Cells were treated with the PPAA polymer alone for 30 min. Cell culture medium containing the polymer treatment was then aspirated, and cells were subsequently treated with fluorescent peptide or vivo-morpholino alone for 30 min.

### Inhibitor uptake studies

HCAVSMCs were pretreated with 100 nM Wortmannin [a covalent inhibitor of phosphoinositide 3-kinases (PI3Ks) that inhibits micropinocytosis], 50 µM 5-(N-ethyl-N-isopropyl) amiloride (EIPA—an inhibitor of Na^+^/H^+^ exchange that inhibits macropinocytosis), 50 µM cytochalasin D (inhibitor of actin polymerization that inhibits micropinocytosis), 100 µM Dynasore (a small molecule inhibitor of dynamin which is essential for clathrin-mediated endocytosis), 5 mM methyl-β cyclodextrin (MβCD, which sequesters and depletes cholesterol from the cell membrane thereby inhibiting lipid raft-mediated endocytosis), 100 µg/mL dextran sulfate (an anionic polymer of sulfated glucose that competitively inhibits scavenger receptor-mediated uptake), or 5 µM latrunculin A (immunological/macrophage phagocytosis inhibitor) for 30 min. Following 30 min of pretreatment, cells were treated with either 5 µM PPAA-RA alone for 30 min, Alexa-488 labeled YARA-MK2i co-delivered with 5 µM PPAA for 30 min, or 5 µM PPAA alone for 30 min followed by Alexa-488 labeled YARA-MK2i (sequential delivery) alone for 30 min. The inhibitors were left on the cells throughout the entire treatment duration. An additional uptake study was performed where the inhibitors were removed after the initial PPAA treatment for sequential delivery to specifically investigate the effects of PPAA uptake inhibition prior to peptide treatment.

### Dynasore-mediated inhibition of PPAA and peptide uptake

HCAVSMCs were pretreated with 0, 50, 100, 150, or 200 µM Dynasore for 30 min. Cells were then treated with PPAA-RA or with unlabeled PPAA alone for 30 min. Cells treated with PPAA-RA were immediately harvested for flow cytometric analysis of polymer uptake, whereas cells treated with unlabeled PPAA were subsequently treated with 5 µM Alexa-488 labeled YARA-MK2i without any inhibitor prior to harvesting for analysis.

### Comparison to commercially available delivery reagents

For comparative analysis of peptide uptake and retention, commercially available delivery reagents were formulated with 5 µM Alexa-488 labeled YARA-MK2i according to the manufacturer’s instructions or with 2.5 µM and 5 µM PPAA for both co-delivery and sequential delivery. HCAVSMCs were seeded and treated for 30 min as noted above. Treatments were then removed and cells were washed 2× in PBS without calcium or magnesium, and subsequently incubated in fresh complete growth medium for an additional 0, 24, 72, or 120 h.

### Uptake of cationic polystyrene nanoparticles

A7r5 cells were treated with 0, 10, 25, 50, 100, 250, or 500 µM PPAA for 30 min, washed, and subsequently treated with red fluorescent Fluospheres diluted in PBS (−/−) to achieve a concentration of 1000 particles/cell for 30 min.

### Uptake of Cy5-DNA-loaded polymeric, micellar nanoparticles

D-DPB NPs were formulated and loaded with Cy5-DNA as noted above. A7r5 cells were treated with 0, 10, 25, 50, 100, 250, or 500 µM PPAA for 30 min, washed, and subsequently treated with Cy5-DNA-loaded D-DPB NPs at a dose of 25 nM Cy5-DNA for 30 min.

### PPAA dose-dependent cytotoxicity

HCAVSMCs were seeded onto 96-well plates at a density of 10,000 cells/well to yield an approximate 70% confluence and allowed to adhere to the plate overnight. Cells were then treated with PPAA polymer alone (doses of 1, 2.5, 5, 10, 20, and 40 µM PPAA corresponding to the mass ratios utilized in flow cytometry assays) for 30 min, with PPAA polymer alone for 30 min followed by treatment with 10 µM YARA-MK2i alone for 30 min (sequential delivery), or with PPAA co-formulated with YARA-MK2i peptide for 30 min (co-delivery) in Opti-MEM medium supplemented with 1% penicillin-streptomycin and 1% FBS. Treatments were subsequently removed and the cells were cultured in complete growth medium for 24 h. Both cytotoxicity (as measured by total cell number) and membrane permeability (as measured by LDH release) were quantified with the CytoTox-ONE Homogenous Membrane Integrity assay (Promega) utilizing the proliferation assay protocol and the membrane integrity assay protocol, respectively. Briefly, for the proliferation assay, cells were washed 2× with PBS with calcium and magnesium and lysed by adding 100 μL of Ambion KDalert Lysis Buffer to each well. Freshly prepared CytoTox-ONE reagent (100 μL) was added to cell lysates and incubated for 10 min at room temperature in the absence of light. After 10 min, 50 μL of stop solution was added and the fluorescence of each well (*λ*_ex_ = 560 nm, *λ*_em_ = 590 nm) was determined with a TECAN Infinite M1000 Pro plate reader. Cell viability was calculated relative to untreated control cells. The membrane integrity assay was similarly performed without washing the cells after 24 h of post-treatment incubation and directly adding the CytoTox-ONE reagent to the media of non-lysed cells.

### Delivery reagent-mediated cytotoxicity

HCAVSMCs were seeded onto 96-well plates as noted above. Commercially available delivery reagents were formulated with 10 µM YARA-MK2i peptide according to the manufacturer’s instructions or with 2.5 and 5 µM PPAA for co-delivery. Cells were treated for 30 min in Opti-MEM medium supplemented with 1% penicillin-streptomycin and 1% FBS. Treatments were subsequently removed and the cells were cultured in complete growth medium for 24 h. Cells were then washed 2× with PBS with calcium and magnesium and cell viability was determined by a CytoTox-ONE Homogenous Membrane Integrity assay utilizing the proliferation assay protocol as noted above.

### Microscopic analysis of peptide endosomal escape

Commercially available delivery reagents were formulated with 5 µM Alexa-488 labeled YARA-MK2i according to the manufacturer’s instructions or with 2.5 µM and 5 µM PPAA for co-delivery. HCAVSMCs were seeded into Lab-Tek II 8-well chambered coverslips at a density of 10,000 cells/well and allowed to adhere overnight. Cells were then treated for 30 min in Opti-MEM medium supplemented with 1% penicillin-streptomycin and 1% FBS. Treatments were subsequently removed, and the cells were cultured in Opti-MEM with 50 nM LysoTracker Red DND-99 for 2 h to enable visualization of acidic endo/lysosomal vesicles. Cells were then washed with 0.1% Trypan blue to quench extracellular fluorescence followed by two additional washes with PBS. Cells were then imaged in complete growth medium using Nikon Eclipse Ti confocal fluorescence microscope with NIS Elements imaging software. Gain settings were set to be constant for all images acquired.

All images were processed using imageJ, and colocalization was analyzed using Just Another Colocalization Plugin (JACoP)^52^. Mander’s overlap coefficients were then calculated for *n* ≥ 3 separate images for each treatment group to quantify colocalization.

### Microscopic analysis of vivo-morpholino endosomal escape

For co-delivery treatments, 2.5 or 5 µM PPAA polymer was mixed with 5 µM fluorescein labeled CD47 vivo-morpholino. For sequential delivery treatments, separate 2.5 µM and 5 µM PPAA stocks and a 5 µM fluorescently labeled vivo-morpholino stock solution were prepared. HMVECs were seeded into Lab-Tek II 8-well chambered coverslips at a density of 10,000 cells/well and incubated overnight. Cells were then treated for 30 min (co-delivery) or for 30 min with PPAA alone followed by 30 min of treatment with fluorescein labeled vivo-morpholino alone (sequential delivery). Cells were then incubated for 30 min in Opti-MEM medium supplemented with 1% penicillin-streptomycin and 1% FBS. Lysotracker staining, imaging of cells, and image processing was carried out as noted above in the peptide endosomal escape experiment.

### Microscopic analysis of peptide and polymer internalization

HCAVSMCs were seeded in into Lab-Tek II 8-well chambered coverslips at a density of 10,000 cells/well and incubated overnight. Cells were then treated with 10 µM Alexa-488 labeled YARA-MK2i alone, 10 µM Alexa-488 labeled YARA-MK2i mixed with 5 µM PPAA-RA (co-delivery), or 5 µM PPAA-RA alone for 30 min. Each treatment group was imaged twice every minute starting 2 min before treatment application utilizing a ×20 objective. After 30 min, treatments were removed and cells treated with the co-delivery method were incubated in fresh medium. Cells treated with 5 µM PPAA-RA alone were subsequently treated with 10 µM Alexa-488 labeled YARA-MK2i alone (sequential delivery). Cells treated with the peptide alone were subsequently incubated in fresh medium. Imaging twice every minute was continued for an additional 90 min (i.e., imaged for 120 min total following initial treatment application). Z-stack images were obtained immediately prior to and after removal of the PPAA-RA and addition of Alexa-488 labeled YARA-MK2i for the sequential delivery treatment. High resolution images were taken of the sequential delivery group 30 min after Alexa-488 labeled YARA-MK2i addition utilizing a ×63 oil-immersion objective.

### Microscopic analysis of vivo-morpholino uptake

HMVECs were seeded into Lab-Tek II 8-well chambered coverslips at a density of 10,000 cells/well and incubated overnight. PPAA polymer was mixed with 5 µM fluorescein labeled CD47 vivo-morpholino at mass ratios ranging from 5:1 to 1:10 (vivo-morpholino:polymer). HMVECs were co-treated for 30 min at each mass ratio and subsequently washed 2× with PBS +/+. Cells were then imaged 2 h later utilizing a Nikon Eclipse Ti confocal fluorescence microscope with NIS Elements imaging software. Gain settings were the same for all images acquired.

### Microscopic analysis of PPAA colocalization with LC3B

HEK 293 T cells stably expressing LC3B-mTurquoise2 were plated in a T-75 flask and cultured for at least 48 h after removal from cryostorage. Cells were seeded at low density (3000 cells per well) into Nunc™ Lab-Tek™ 8-Chambered Coverglass (Catalog 155361, ThermoFisher Scientific) in DMEM supplemented to 10% FBS. After adhering overnight, cells were treated with 5 μM PPAA-RA for 30 min, washed with media, and allowed to incubate in media 24 h. At 24 h post wash, media was removed and replaced with FluoroBrite DMEM (catalog A1896701) supplemented with 25 mM HEPES (Gibco™ 15630056) and 10% FBS. Cells were monitored by time-lapse confocal microscopy under optimal conditions on a Nikon C1si with appropriate excitation and emission filters. The lookup table was altered to highlight vesicular structures. Images were false colored green/magenta and had lookup tables optimized to highlight vesicular structures. NIS Elements was used to normalize brightness across the time-lapse imaging and perform minimum intensity projection subtraction. Images were exported as movies (Supplementary Movie 4) and still frames (Fig. 4h).

### Microscopic analysis of mitochondrial PPAA colocalization

HCAVSMCs were seeded in into Lab-Tek II 8-well chambered coverslips at a density of 10,000 cells/well and incubated overnight. Cells were treated with 5 µM PPAA for 30 min. Cells were stained with MitoTracker Green, CellTracker Green, or Lysotracker Red according to the manufacturer’s instructions. Nuclei were counterstained with DAPI. Images were captured utilizing a ×20 objective.

### Split-GFP peptide intracellular bioavailability assay

Stably expressing GFP1-10 A7r5 rat smooth muscle cells were expanded and seeded in 12-well plates at a density of 60,000 cells/well in DMEM with HEPES supplemented with 10% FBS and 1% P/S and incubated overnight. A dose of 30 µM YARA-SS-GFP11β peptide was utilized based on prior work and preliminary testing demonstrating that 30 µM of the peptide alone is required to consistently yield a detectable increase in GFP fluorescence. Prior to treatment application, the media was replaced with Opti-MEM supplemented with 1% FBS and 1% P/S. For Co-delivery, cells were treated with 30 µM YARA-SS-GFP11β peptide alone, YARA-SS-GFP11β mixed with either 2.5 or 5 µM PPAA, YARA-SS-GFP11β formulated with Xfect according to the manufacturer’s instructions, or left untreated (negative control). For sequential treatment, cells were first treated with either 2.5 or 5 µM PPAA alone for 30 min followed by treatment with 30 µM YARA-SS-GFP11β peptide alone for 30 min. A peptide dose of 30 µM was chosen as it is the minimal dose of peptide that yields a significant increase in GFP fluorescence. Following treatment, the cells were washed 1× with PBS and incubated in fresh medium (DMEM with 10% FBS and 1% P/S) for 4 h. Following post-treatment incubation, cells were harvested, and flow cytometric analysis of intracellular GFP11β induced GFP fluorescence was performed as detailed in the flow cytometry section above. All flow cytometric bioavailability data are presented as fold increase in YARA-SS-GFP11β peptide induced GFP fluorescence relative to treatment with the YARA-SS-GFP11β peptide alone.

### NanoLuc luciferase intracellular bioavailability assay

HEK 293-T LgBiT cells were seeded into a collagen-coated 96-well plate in DMEM with 10% FBS and 1% P/S and allowed to incubate for 24 h. The media was then removed and cells were treated with 5 μM PPAA or 0 μM PPAA for 30 min in Opti-Mem with 1% FBS and 1% P/S. After 30 min of treatment, the PPAA was removed and cells were treated with either 1 or 0.5 μM YARA-HiBiT media for 30 min. The treatment was then removed and cells were incubated in 10% FBS media for 6 h. Prior to reading, the TECAN Infinite M1000 Pro plate reader was warmed to 37 °C. The cells were treated with 1 μM DrkBiT for 2 min, after which the NanoLuc substrate was added. The plate was immediately read on the plate reader in a kinetic luminescence cycle for 20 min. Peak luminescence data points were calculated by taking the average of the five readings at the peak of the kinetic curve.

### Cellular surface charge assessment

HCAVSMCs were grown in T-75 flasks in complete growth medium to 80–90% confluence. Cells were trypsinized and harvested and split into six separate tubes containing 50,000 cells in 1 mL of PBS−/−. Cells were treated with 10 µM YARA-MK2i alone, 2.5 or 5 µM PPAA alone, or left untreated for 30 min. For sequential delivery, cells were treated with 2.5 or 5 µM PPAA alone for 30 min, centrifuged, washed 2× with PBS, and resuspended in 1 mL of PBS containing 10 µM YARA-MK2i peptide. Following treatment, repeated washing, and resuspension, the zeta potential (i.e., ζ-potential, or electrokinetic potential in a colloidal dispersion) of each treatment group was quantified utilizing a Malvern Zetasizer Nano ZS with a reusable dip-cell kit in 1 mL disposable cuvettes.

### TAT-CRE Ai9 gene recombination assay

Ai9 NIH/3T3 cells were first pretreated with 0, 0.05, 0.78, 12.5, or 200 ng/µL (2 nM–1 µM) PPAA in 1% Serum OptiMEM in half-area 96-well plates (Corning 4580). After 30 min, the media was removed by flicking and 1% Serum OptiMEM containing TAT-CRE at doses of 20, 30, or 40 units/mL was added. Note that these concentrations were purposefully chosen to cover a sub-active concentration range based on pilot studies on delivery of TAT-CRE alone. The cells were incubated for 24 h, then the media was changed to Full Serum DMEM without Phenol Red and the cells were imaged and counted using the same equipment, software, and methods as noted in the CRISPR/Cas9 RNP Ai9 gene editing section above, but in half-area 96-well plates imaged with a ×20 objective; *n* = 25 images per well.

### Luciferase gene-silencing assay

Luciferase expressing A7r5 cells were seeded in a black, clear bottom 96-well plate at a density of 10,000 cells/well in DMEM containing 10% FBS and 1% P/S and allowed to adhere overnight. D-DPB NPs, PepFect-siRNA polyplexes, and CADY-siRNA polyplexes were formulated and loaded with luciferase or scrambled siRNA as noted above. A7r5 cells were treated with 0, 10, 25, 50, 100, 250, or 500 µM PPAA for 30 min, washed, and subsequently treated with siRNA-loaded D-DPB NPs, PepFect-siRNA polyplexes, or CADY-siRNA polyplexes at a dose of 25 nM siRNA for 30 min in Optimem supplemented with 1% FBS and 1% P/S. Following treatment, cells were incubated for an additional 24 h in in DMEM containing 10% FBS and 1% P/S. Following post-treatment incubation, media was removed and cells were treated with luciferin (150 µg/mL) before imaging on a Lumina III IVIS (Cailper Life Sciences). Luciferase gene silencing was calculated compared with cells treated with an equivalent dose of scrambled siRNA loaded into D-DPB NPs as a control.

### Gal8 recruitment assay

Gal8-MDA-MB-231 cells were plated in 384-well plates (Greiner 781091) at a density of 750 cells/well using a BioTek EL406 liquid aspirator/dispenser and allowed to adhere overnight. For Bafilomycin and Nocodazole inhibitor studies, cells were pretreated with 100 nM bafilomycin A or 20 µM Nocodazole for 30 min. Cells were subsequently treated with 0 or 10 µM PPAA for 30 min in the presence of inhibitors. Following treatmeant, the cells were washed and inhibitors replaced. The cells were then imaged over 3 h post-treatment as outlined below. For CRISPR/Cas9 RNP delivery studies, cells were pretreated with 0, 25, 50, 100, 250, or 500 ng/mL PPAA in opti-MEM with 1% FBS and 1% P/S for 30 min using the BioTek EL406. The media was then aspirated and cells were subsequently treated using a Bravo automated pipette liquid transfer system (Velocity 11/Agilent). Modified Ai9-L and Ai9-R sgRNAs (Synthego) and TrueCut Cas9 Protein V2 (ThermoFisher) were utilized to formulate Cas9 RNPs. Cells were treated with Cas9 RNP complexed with Lipofectamine^TM^ CRISPRMAX^TM^ according to manufacturer specifications, Xfect according to manufacturer specifications, D-DBP NPs at a mass ratio of 8:1 D-DPB:Cas9, or left untreated in opti-MEM with 1% FBS and 1% P/S. Following 1 h of treatment, cells were washed twice with DMEM containing 10% FBS and 1% P/S, then stained in FluoroBrite DMEM (ThermoFisher Scientific) supplemented with 10% FBS and 1% P/S containing Nucblue Live ReadyProbes nuclear stain (ThermoFisher Scientific) for 15 min. The cells were imaged with a ×20 objective in an ImageXpress Micro XLS Widefield High-Content Analysis System. Cells were analyzed using MetaXpress software Transfluor Application module to quantify the number of vesicles per/nuclei from *n* = 3 images per well, with ~1500 nuclei captured per image.

### CRISPR/Cas9 Ai9 gene editing assay

Ai9 NIH/3T3 cells were plated, treated, and imaged as described in the Gal8 recruitment assay section. Instead of imaging at 1 h, however, the cells were washed (removing the treatment) after 4 h, and imaged after ~48 h with a ×10 objective and *n* = 4 images per well covering almost the entire well area. Approximately 1500 cells were counted in each image. Cells were analyzed using MetaXpress software Multi-wavelength cell scoring Application module to quantify the number of nuclei and the number of cells with cytosolic tdTomato.

### Statistics

Statistical analyses were performed with a one-way ANOVA followed by Tukey’s post hoc test to compare experimental groups. Analyses were performed with Graphpad Prism Software Version 7.02 (La Jolla, California). Statistical significance was accepted within a 95% confidence limit. Results presented are arithmetic means ± SEM graphically, and *p* values are included within the figures or figure captions.

### Reporting summary

Further information on research design is available in the Nature Research Reporting Summary linked to this article.

## Supplementary information

Supplementary Information

Reporting Summary

Description of Additional Supplementary Files

Supplementary Movie 1

Supplementary Movie 2

Supplementary Movie 3

Supplementary Movie 4

## Data Availability

The datasets generated during and/or analyzed during the current study are available from the corresponding author on reasonable request.
