# Supplementary material for: An anionic, endosome-escaping polymer to potentiate intracellular delivery of cationic peptides, biomacromolecules, and nanoparticles
Source: Nat Commun. 2019 Nov 1;10:5012. doi: 10.1038/s41467-019-12906-y (PMC6825215; doi:10.1038/s41467-019-12906-y)
Supplement: Supplementary file 1 — Supplementary Information [file 41467_2019_12906_MOESM1_ESM.pdf]

Supplementary Information - An Anionic, Endosome-Escaping Polymer to Potentiate Intracellular Delivery of Cationic Peptides, Biomacromolecules, and Nanoparticles

Evans et al.

Supplementary Methods:

PPAA Uptake and the Effects of Fluorescent Labeling on Peptide and Polymer Uptake

HCAVSMCs were treated with Alexa-488 labeled YARA-MK2i (YARA-MK2i-488) alone for 30 minutes, Rhodamine acrylate labeled PPAA (PPAA-RA) alone for 30 minutes, PPAA-RA alone followed by YARA-MK2i-488 alone (sequential delivery, 30 minutes each), YARA-MK2i-488 alone followed by PPAA-RA alone (sequential delivery, 30 minutes each), co-delivery of YARA-MK2i-488 and PPAA-RA, co-delivery of unlabeled YARA-MK2i and PPAA-RA, or co-delivery of YARA-MK2i-488 and unlabeled PPAA (PPAA and PPAA-RA dose = 2.5 µM, YARA-MK2i and YARA-MK2i-488 dose = 5 µM). Flow Cytometric analysis of peptide and polymer uptake was subsequently performed as described in the main text.

Supplementary Figures:

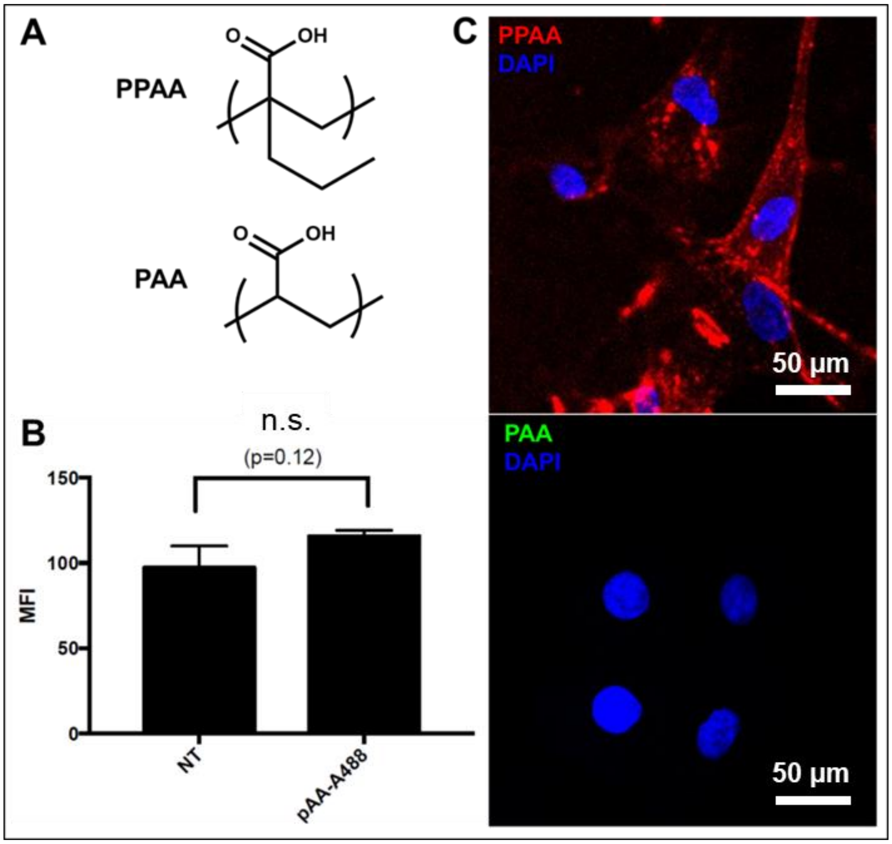

Supplementary Figure 1 – Hydrophobic propyl alkyl α-substitution of acrylic acid drives cellular membrane interactions. A) Chemical structure of poly(propylacrylic acid) (PPAA) and poly(acrylic acid) (PAA). B) Uptake of alexa-488 labeled PAA polymer in A7r5 cells is not significant; one-way ANOVA. Data is presented as means ± SEM. C) Representative images of rhodamine labeled PPAA and alexa-488 labeled PAA coating of A7r5 cell membrane after 30 minutes of treatment demonstrating robust membrane interactions with PPAA in contrast to negligible interactions with PAA.

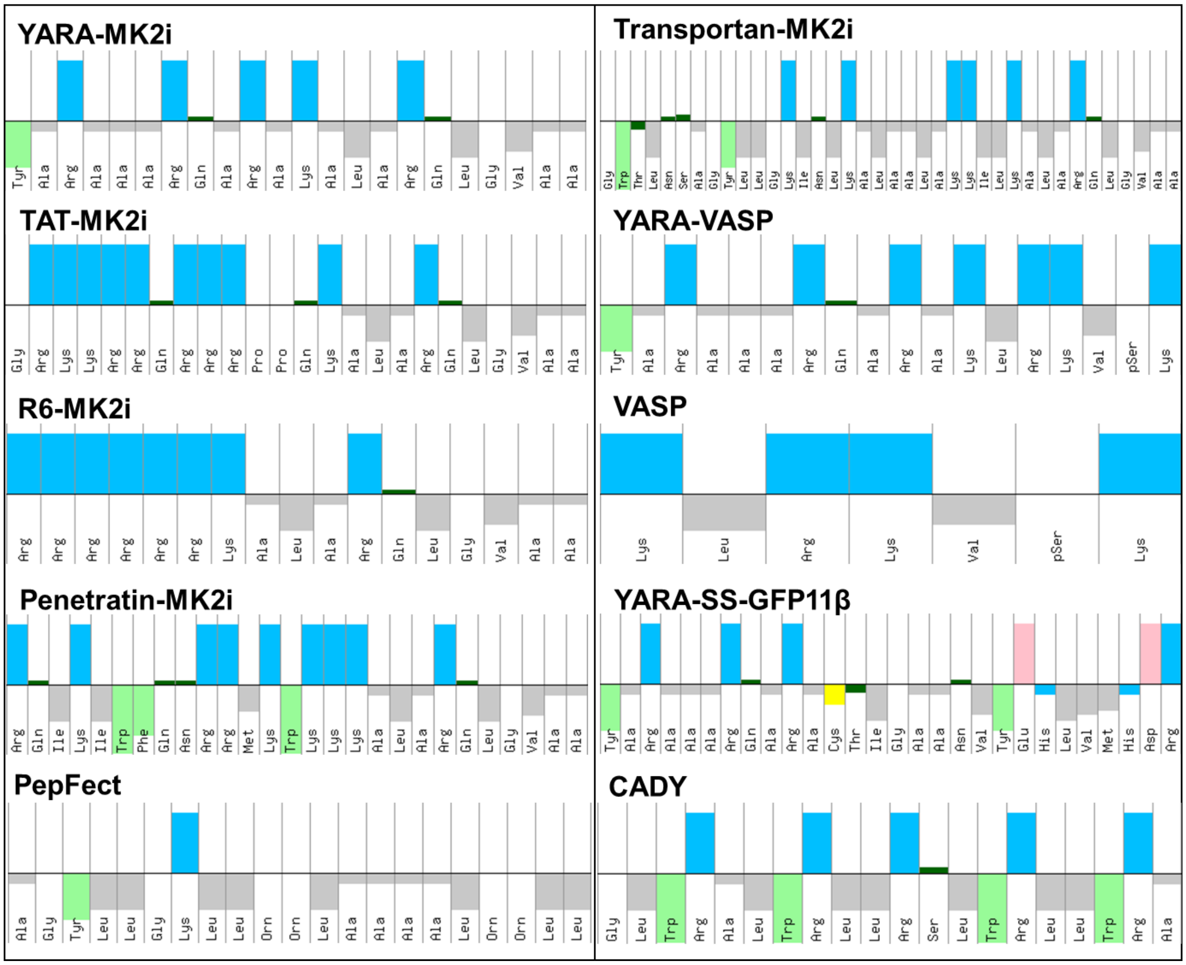

Supplementary Figure 2 - Hopp & Woods Hydropathy Plots for all model CPP-peptides and CPP-based vectors listed in table 1. For the PepFect peptide, the N-terminal stearyl modification is not included. For the CADY peptide, the N-terminal acetylation and C-terminal cysteamide are not included.

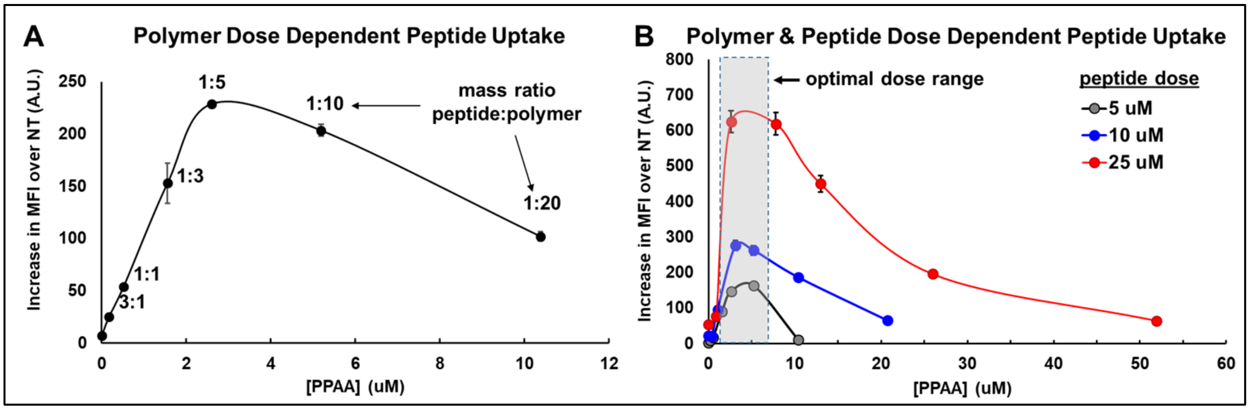

Supplementary Figure 3 – Polymer and peptide dose dependent effects on cellular peptide uptake for PPAA-peptide co-delivery. A) Polymer dose dependent uptake of 10 µM YARA-MK2i in HCAVSMCs demonstrating peptide uptake saturates and then decreases at higher polymer concentrations. B) Polymer dose dependent uptake of the YARA-MK2i peptide at doses of 5 µM, 10 µM, and 25 µM peptide in HCAVSMCs. The mass ratios used for each dose of peptide are 3:1, 1:1, 1:3, 1:5, 1:10, and 1:20 peptide:polymer. A co-delivery treatment of 30 minutes was utilized for all data shown. Data are presented as means ± SEM

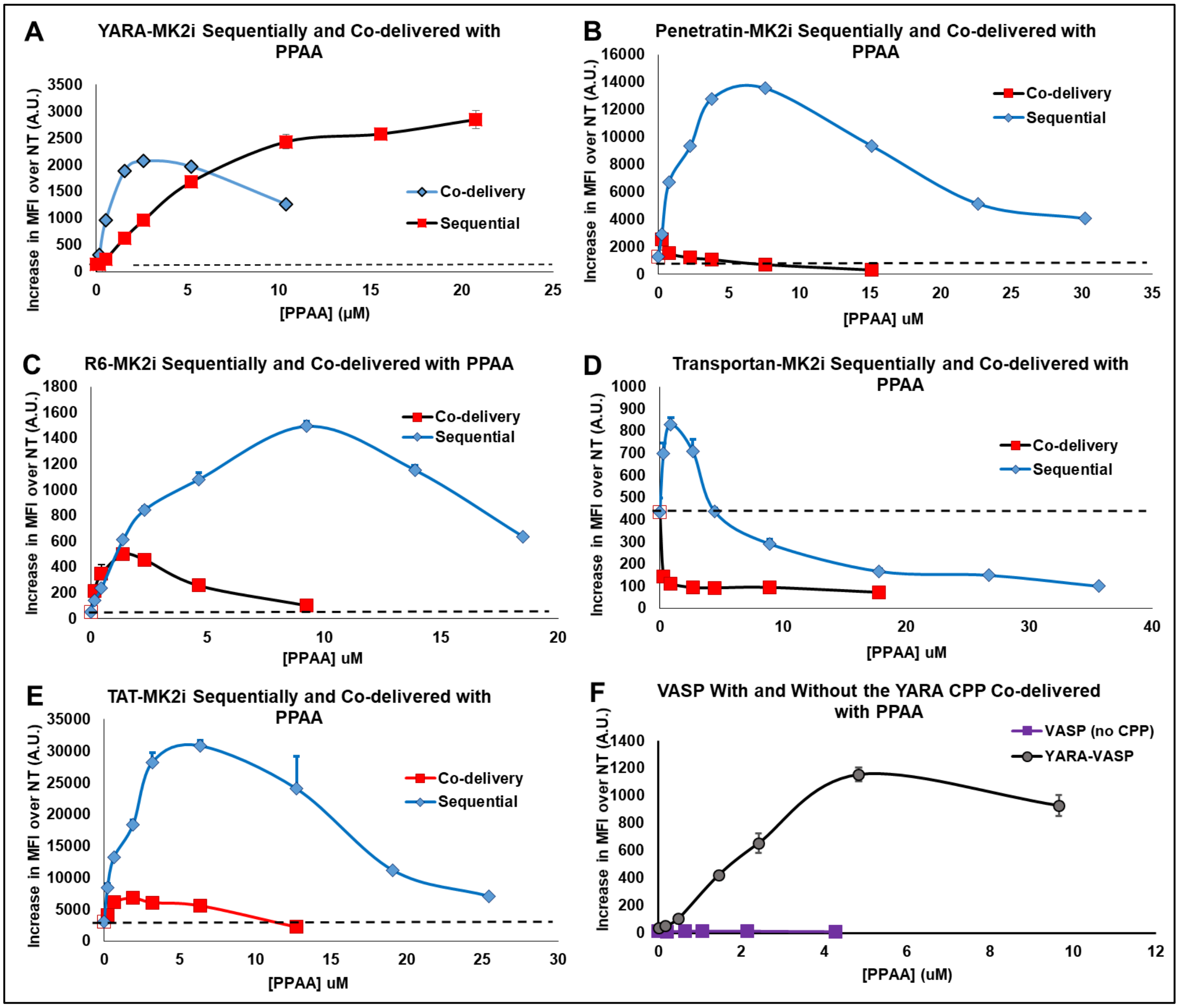

Supplementary Figure 4 – Un-normalized sequential versus co-delivery uptake of cationic CPP-modified MK2i peptides. Uptake of the CPP modified MK2i peptides A) YARA-MK2i, B) Penetratin-MK2i, C) R6-MK2i, D) Transportan-MK2i, and E) TAT-MK2i following co-delivery and sequential delivery with a range of doses of PPAA in human coronary artery vascular smooth muscle cells (HCAVSMCs). F) Uptake of the VASP peptide both with and without the YARA-CPP delivered with a range of doses of PPAA in HCAVSMCs. Fluorophore labeling efficiency for each peptide cannot be assumed to be equivalent, so direct comparisons between levels of uptake for different peptides should not be made. Data are presented as means ± SEM

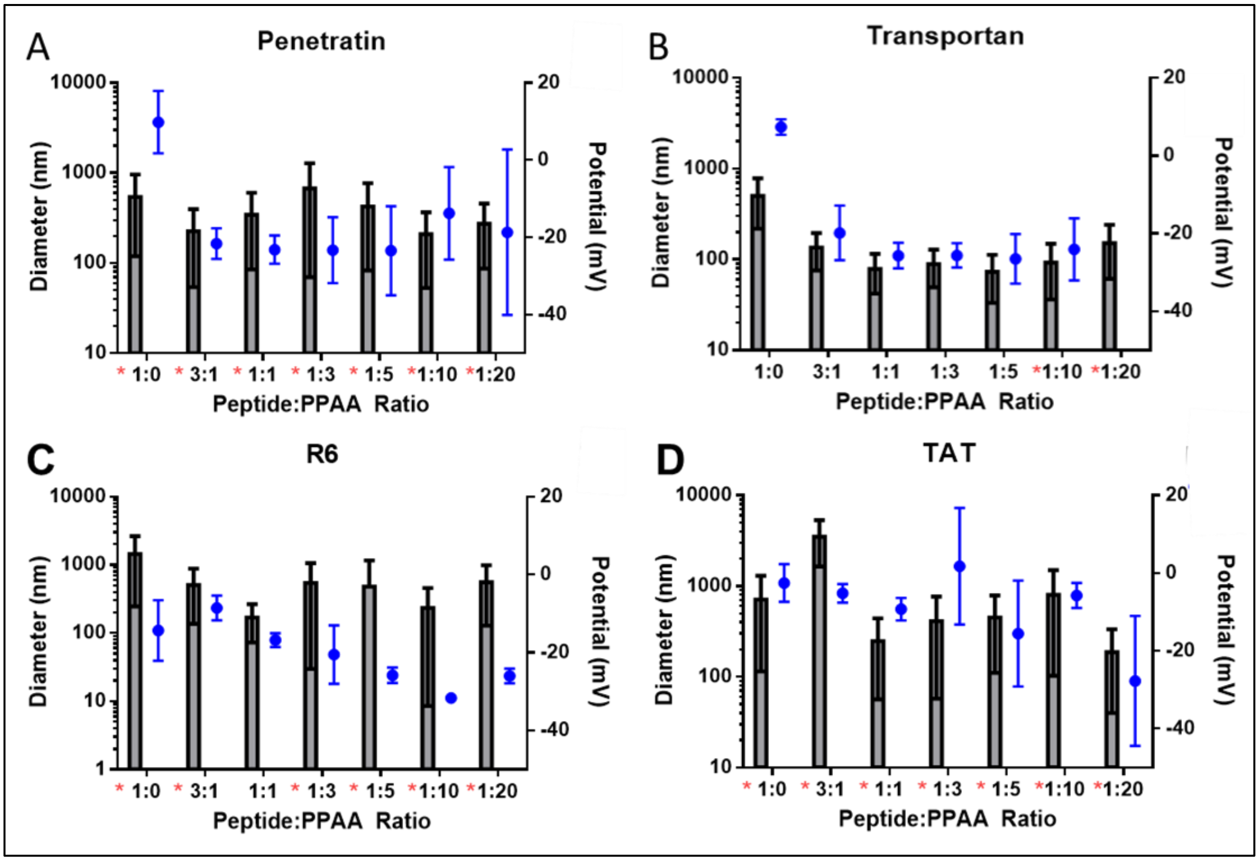

Supplementary Figure 5 – Size and surface charge (ζ-potential) of electrostatically complexed PPAA and CPP-modified MK2i peptides. DLS based analysis of PPAA:peptide mass ratio dependent complex size (gray bars) and ζ-potential (blue dots) for: A) Penetratin-MK2i B) Transportan-MK2i C) R6-MK2i, and D) TAT-MK2i. Red asterisk indicate samples with multimodal size distributions. Data are presented as means ± SEM

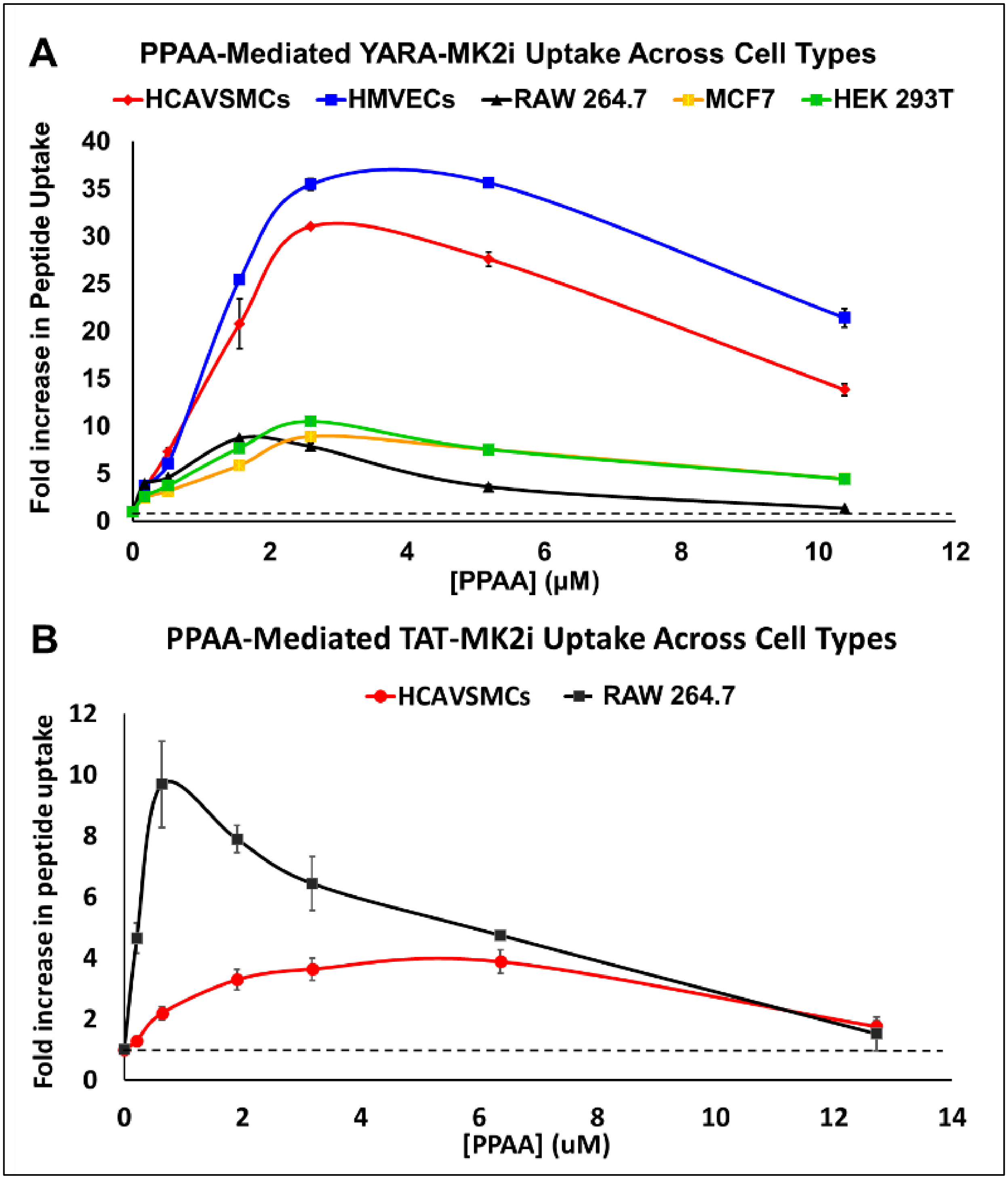

Supplementary Figure 6 – PPAA enhances cationic peptide uptake (using co-delivery) across several cell types. A) Polymer dose dependent uptake of the YARA-MK2i peptide in HCAVSMCs, HMVECs, RAW 264.7, MCF7, and HEK 293T cells. B) Polymer dose dependent uptake of the TAT-MK2i peptide in HCAVSMCs and RAW 264.7 cells. Dashed lines represent the level of uptake of the peptide alone (no polymer). The mass ratios used for all data shown are 3:1, 1:1, 1:3, 1:5, 1:10, and 1:20 peptide:polymer. Non-normalized uptake for all data presented in this figure is shown in Supplementary Figure 7. Data are presented as means ± SEM.

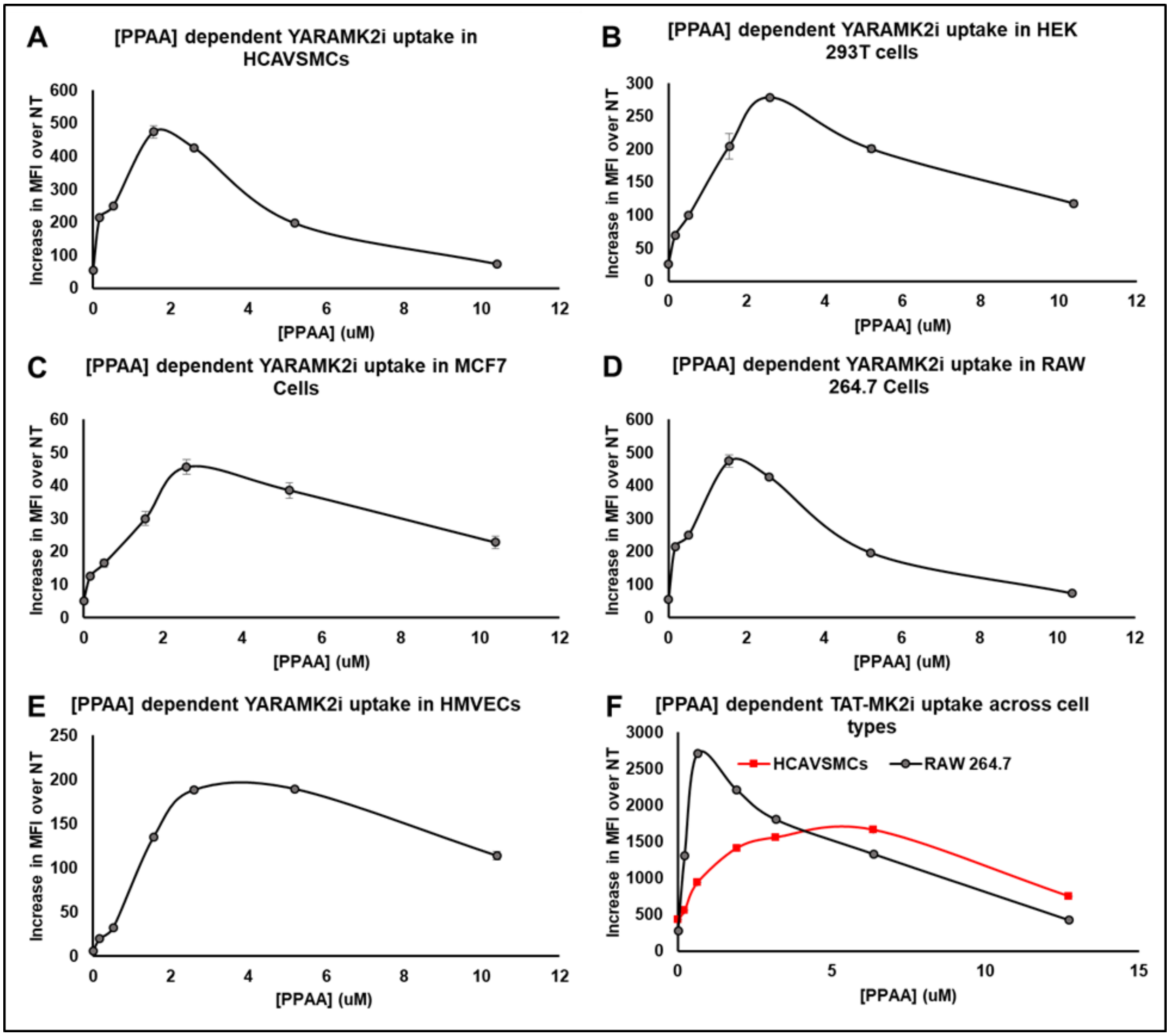

Supplementary Figure 7 – Un-normalized uptake of YARA-MK2i peptide co-delivered with PPAA in various cell lines. Uptake of the YARA-MK2i peptide co-delivered with a range of doses of PPAA in A) human coronary artery vascular smooth muscle cells (HCAVSMCs), B) human embryonic kidney cells (HEK 293T), C) human mammary gland epithelial breast cancer cells (MCF7), D) murine leukemic monocyte macrophages (RAW 264.7), and E) human microvascular endothelial cells (HMVECs) following co-delivery and sequential delivery with a range of doses of PPAA. F) Uptake of theTAT-MK2i peptide co-delivered with a range of doses of PPAA in HCAVSMC and RAW 264.7 cell lines. Data are presented as means ± SEM. Fluorophore labeling efficiency for each peptide cannot be assumed to be equivalent, so direct comparisons between levels of uptake for YARA-MK2i and TAT-MK2i should not be made.

Manufacturer	Product	Type (material)	Recommended treatment conditions
Pierce Biotechnology	Pro-Ject	Polycation (lipid)	3-4 hours (serum free)
Polyplus (VWR)	PULSin	Polycation (amphiphile)	4 hours (serum free)
EMD Biosciences	ProteoJuice	Polycation (unspecified)	2-29 hours (serum free)
Gene Therapy Systems	BioPorter	Polycation (lipid)	3-4 hours (serum free)
Targeting Systems	Profect-1	Polycation (1-lipid)	2-5 hours (serum free) + serum out to 24 hours
Targeting Systems	Profect-2	Polycation (non-lipid)	2-5 hours (serum free) + serum out to 24 hours
Clontech	Xfect	Polycation	4-12 hours (with serum)

Supplementary Table 1 – Commercially available peptide and protein delivery reagents.

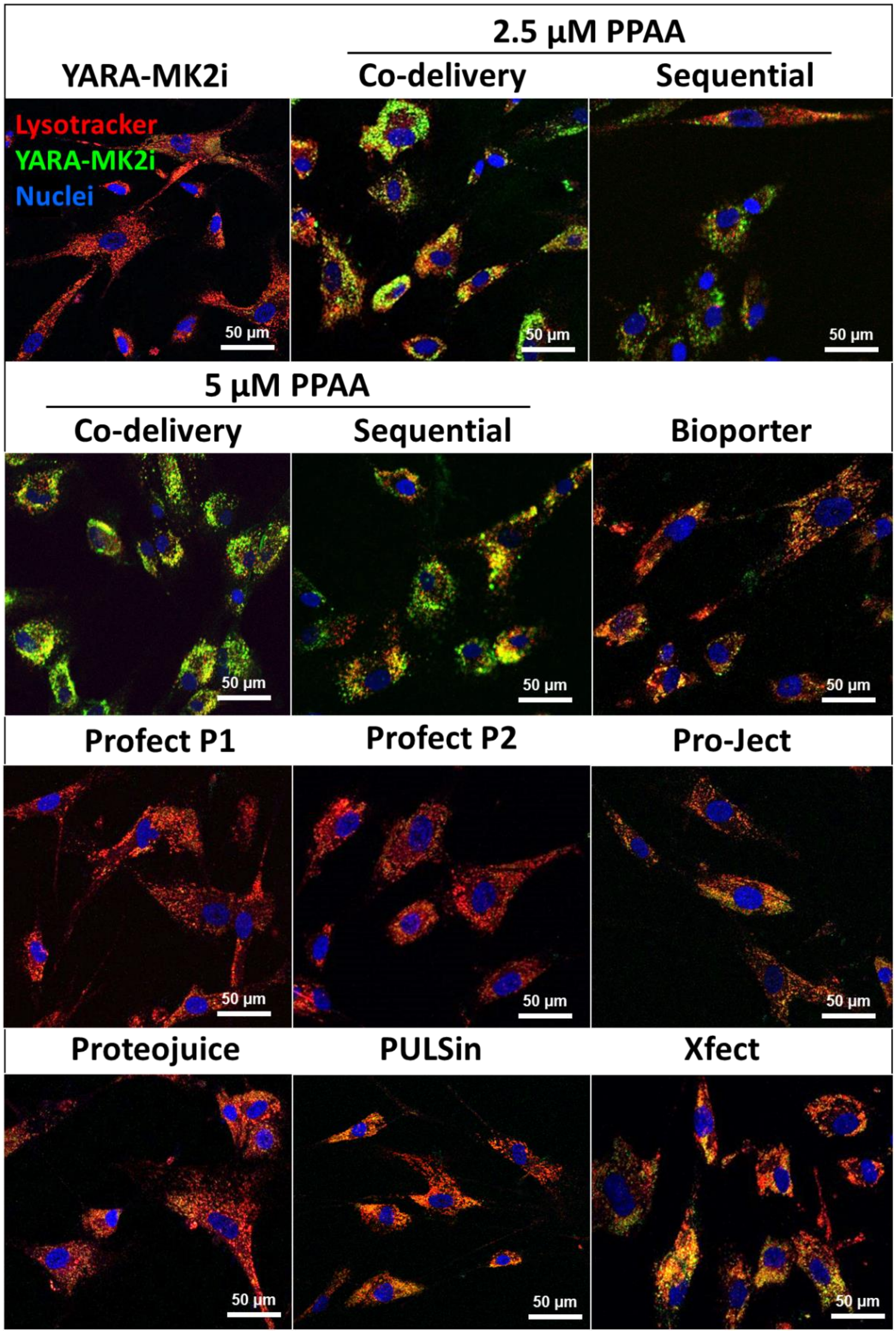

Supplementary Figure 8 – Fluorescence confocal microscopy images comparing the ability of commercially available delivery reagents to mediate peptide endosomal escape. Representative fluorescence confocal microscopy images of YARA-MK2i (green) colocalization with the endosomal dye LysoTracker Red DND-99 (Red) in human coronary artery vascular smooth muscle cells.

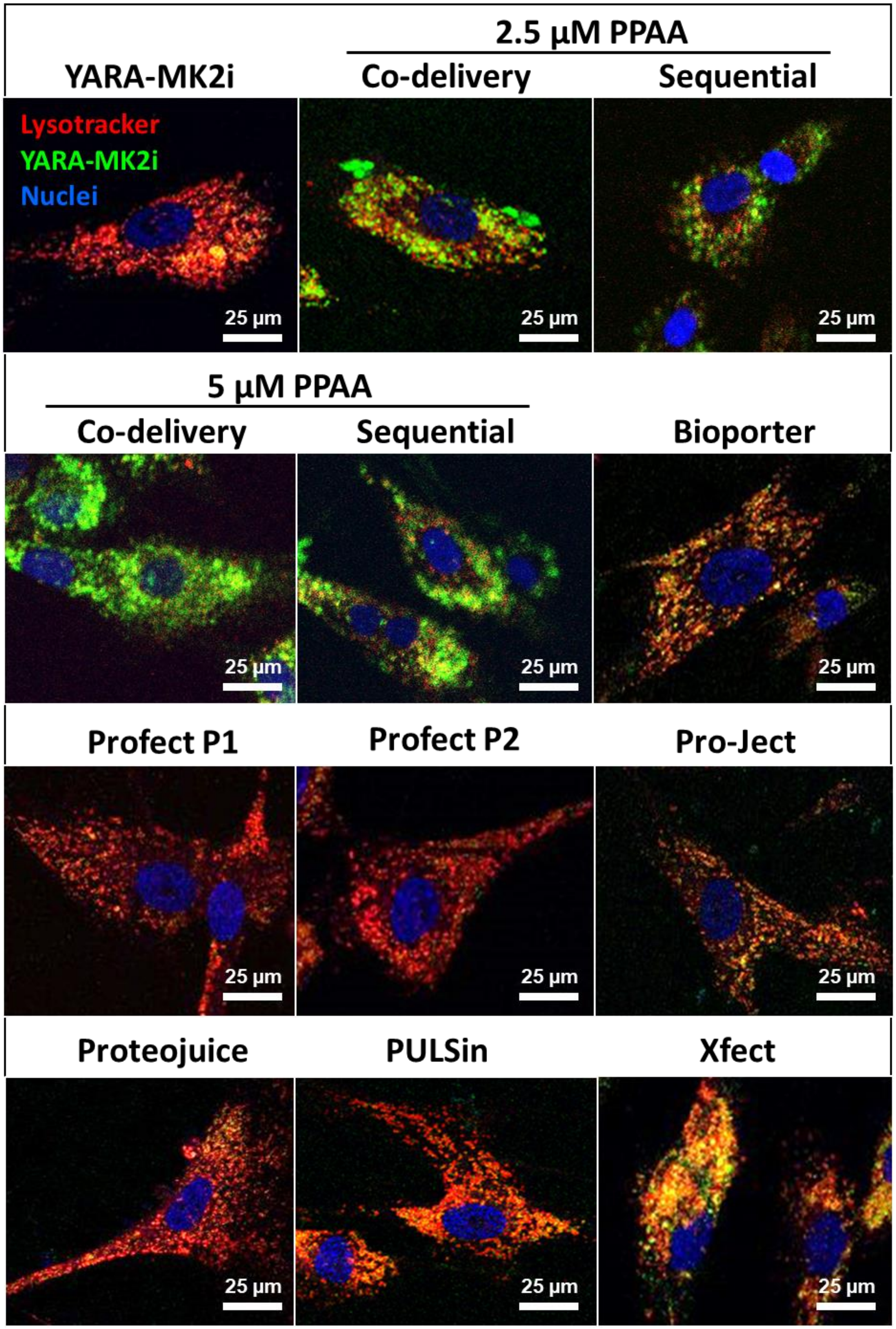

Supplementary Figure 9 – Zoomed fluorescence confocal microscopy images from Supplementary Figure 8.

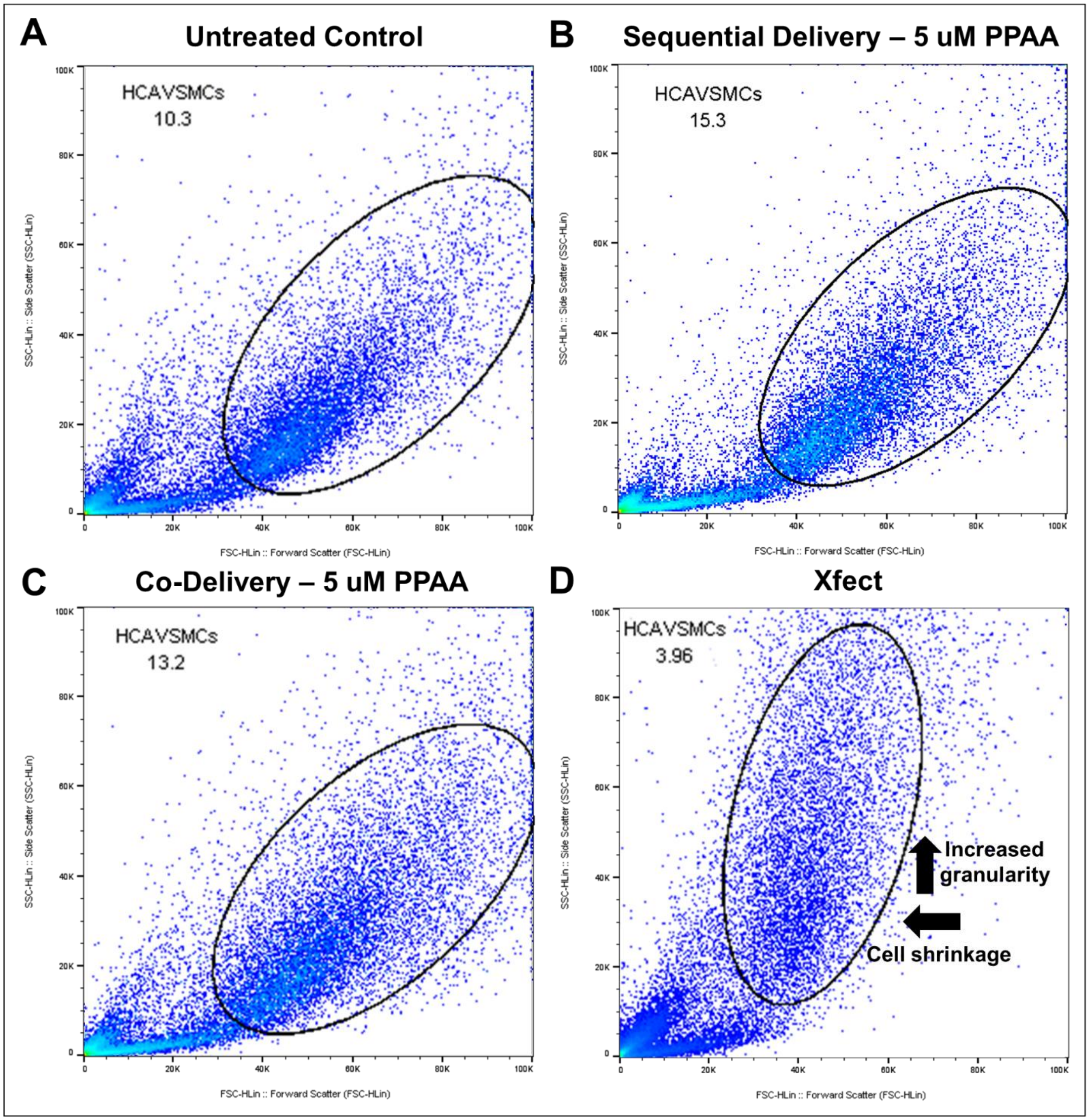

Supplementary Figure 10 – Forward scatter (FSC) vs. side scatter (SSC) plots from flow cytometric analysis of delivery reagent mediated peptide uptake. Representative FSC vs. SSC plots for A) untreated controls cells, cells treated with B) sequential delivery of 5 µM PPAA followed by 10 µM YARA-MK2i peptide, C) Co-delivery of 5 µM PPAA pre-complexed 10 µM YARA-MK2i peptide, and D) Xfect pre-complexed with 10 µM YARA-MK2i peptide demonstrating that treatment with Xfect results in decreased cell size and enhanced granularity suggestive of cell apoptosis.

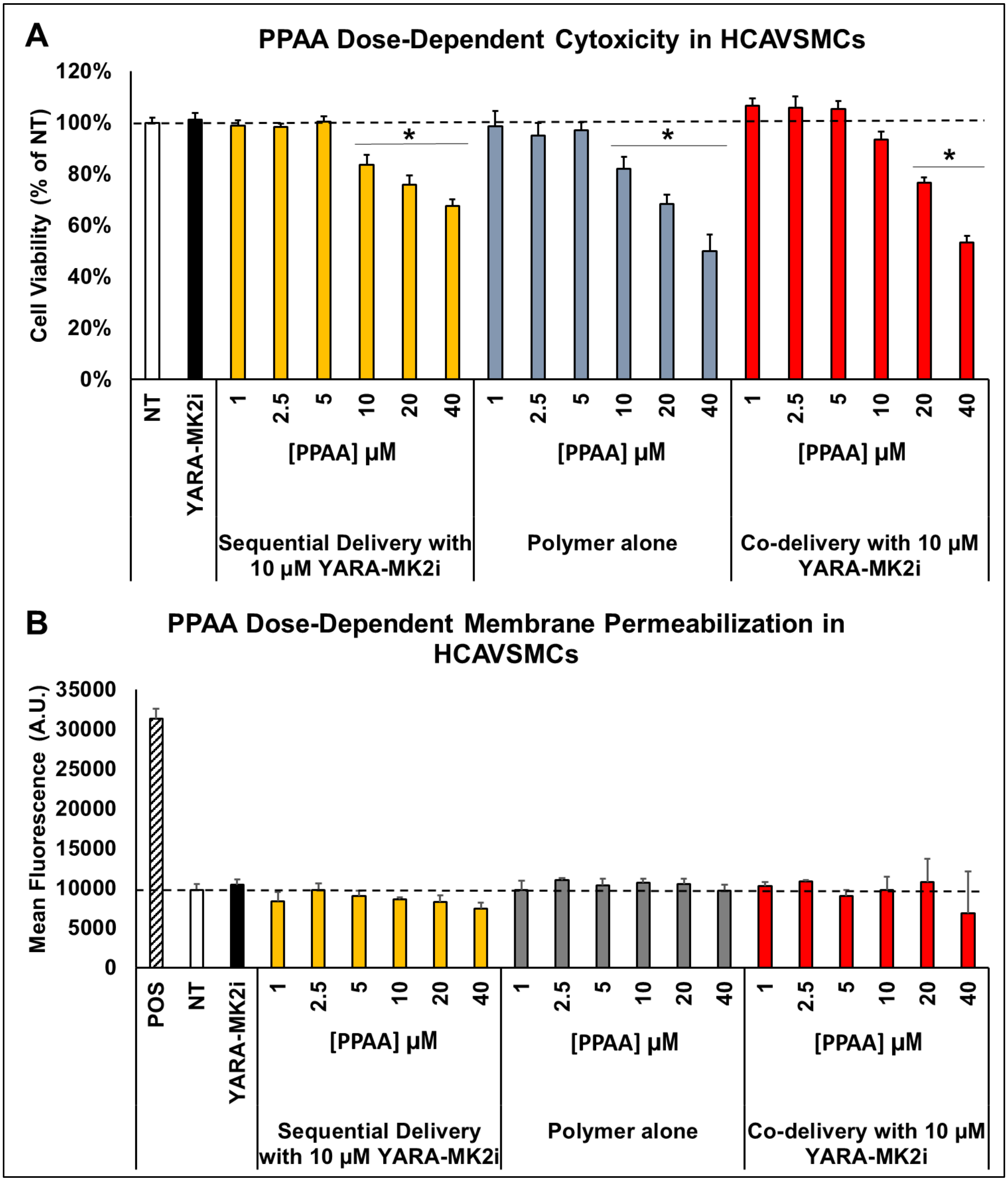

Supplementary Figure 11 – PPAA dose dependent cytotoxicity in human coronary artery vascular smooth muscle cells with and without co-delivery or sequential delivery of 10 µM of the YARA-MK2i peptide. A) Cell viability (total cell number measured based on LDH content of cell lysates) and B) membrane integrity (LDH release into media) 24 hours post-treatment removal. *p<0.01 vs. NT; one-way ANOVA followed by Tukey's post-hoc test. Data are presented as means ± SEM. POS represents positive control cells lysed with 1% Triton X-100.

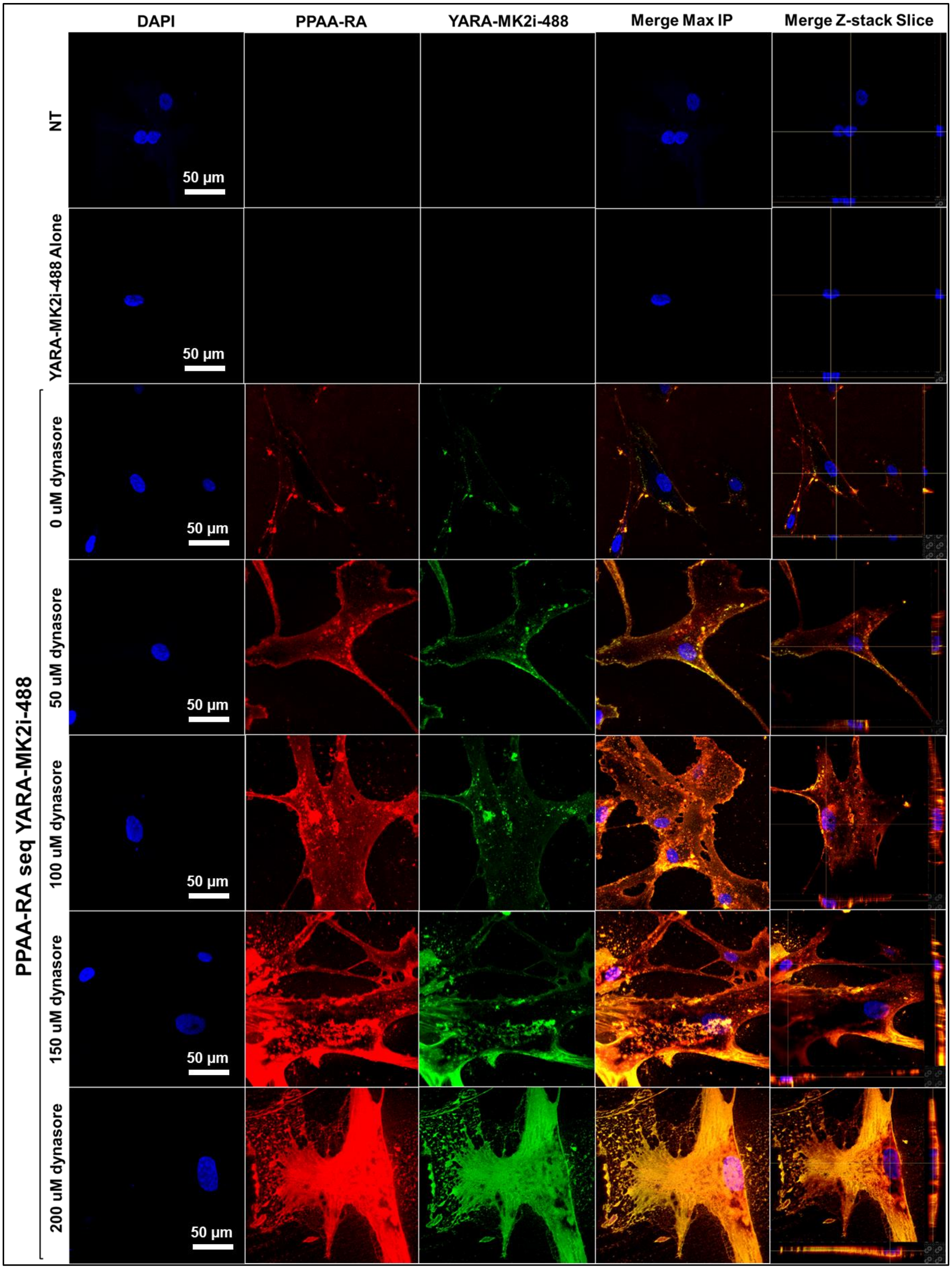
DAPI
PPAA-RA
YARA-MK2i-488
Merge Max IP
Merge Z-stack Slice
NT
YARA-MK2i-488 Alone
PPAA-RA seq YARA-MK2i-488
0 uM dynasore
50 uM dynasore
100 uM dynasore
150 uM dynasore
200 uM dynasore
50 µm

Supplementary Figure 12 – Microscopic analysis of dynasore dose-dependent effects on fluorescently labeled polymer and peptide uptake suggests that dynasore blocks polymer internalization, causing accumulation on the cell surface and consequently strong baiting of peptide onto the cell surface. HCAVSMCs were left untreated (NT), treated with Alexa 488 labeled peptide alone (YARA-MK2i-488), or sequentially (seq) treated with PPAA-RA followed by YARA-MK2i-488. Cells undergoing sequential treatment were pre-treated for 30 minutes with a range of concentrations of the uptake inhibitor dynasore, which was also included during PPAA pre-treatment and subsequently removed prior to addition of the YARA-MK2i-488 peptide. Each treatment was applied for 30 minutes, with sequential treatments having 30 minute treatment durations for both the polymer and peptide. Images were taken after 30 minutes of peptide treatment with equivalent gain settings. Max IP – maximum intensity projection. Merge Z-stack slice images have x and y projections of 3-dimensional z-stack images below and to the right of each image, respectively.

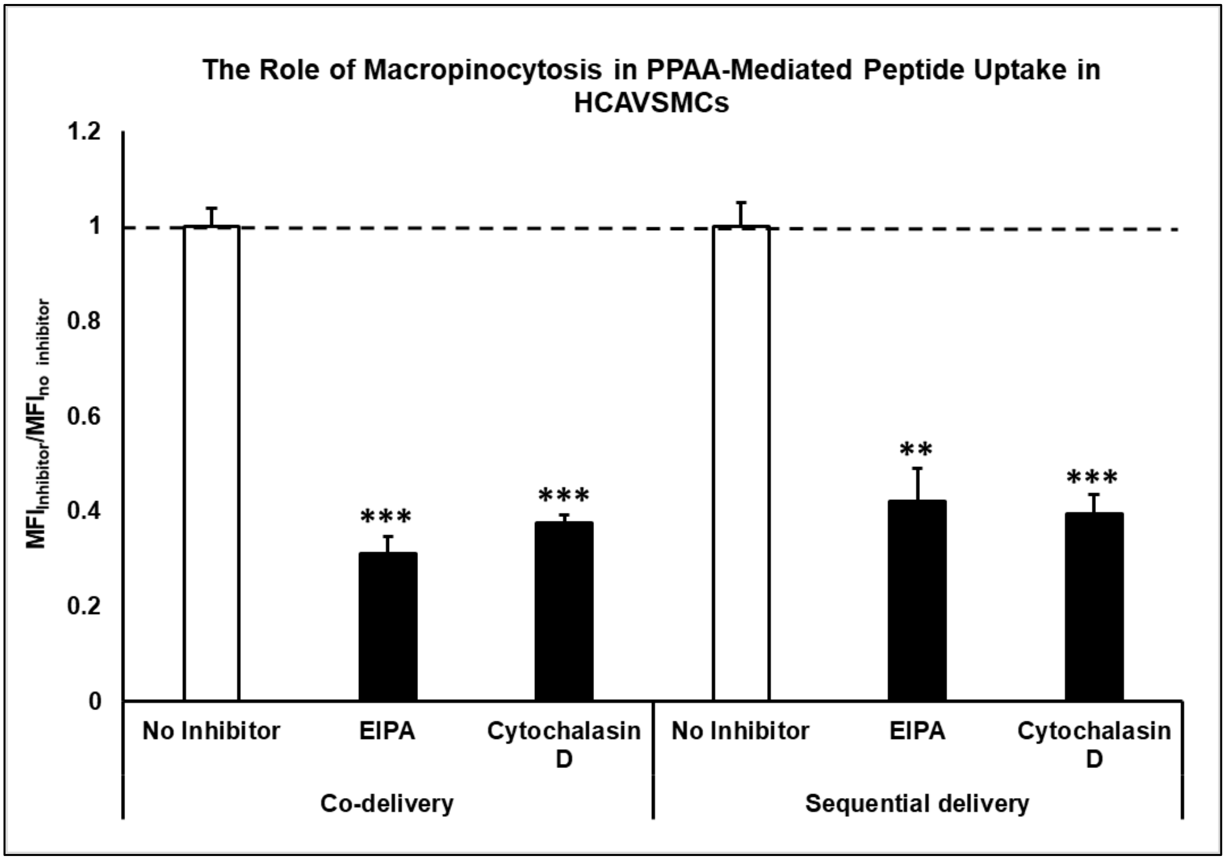

Supplementary Figure 13 – Effect of additional macropinocytosis inhibitors on peptide uptake when sequentially vs. co-delivered in human vascular smooth muscle cells. Inhibition of YARA-MK2i peptide uptake when co-delivered vs. sequentially delivered with 5 µM PPAA in HCAVSMCs in the presence of the micropinocytosis inhibitors Cytochalasin D and amiloride (EIPA). $**p<0.01$, $***p<0.001$ vs. no inhibitor; one-way ANOVA followed by Tukey's post-hoc test. Data are presented as means ± SEM.

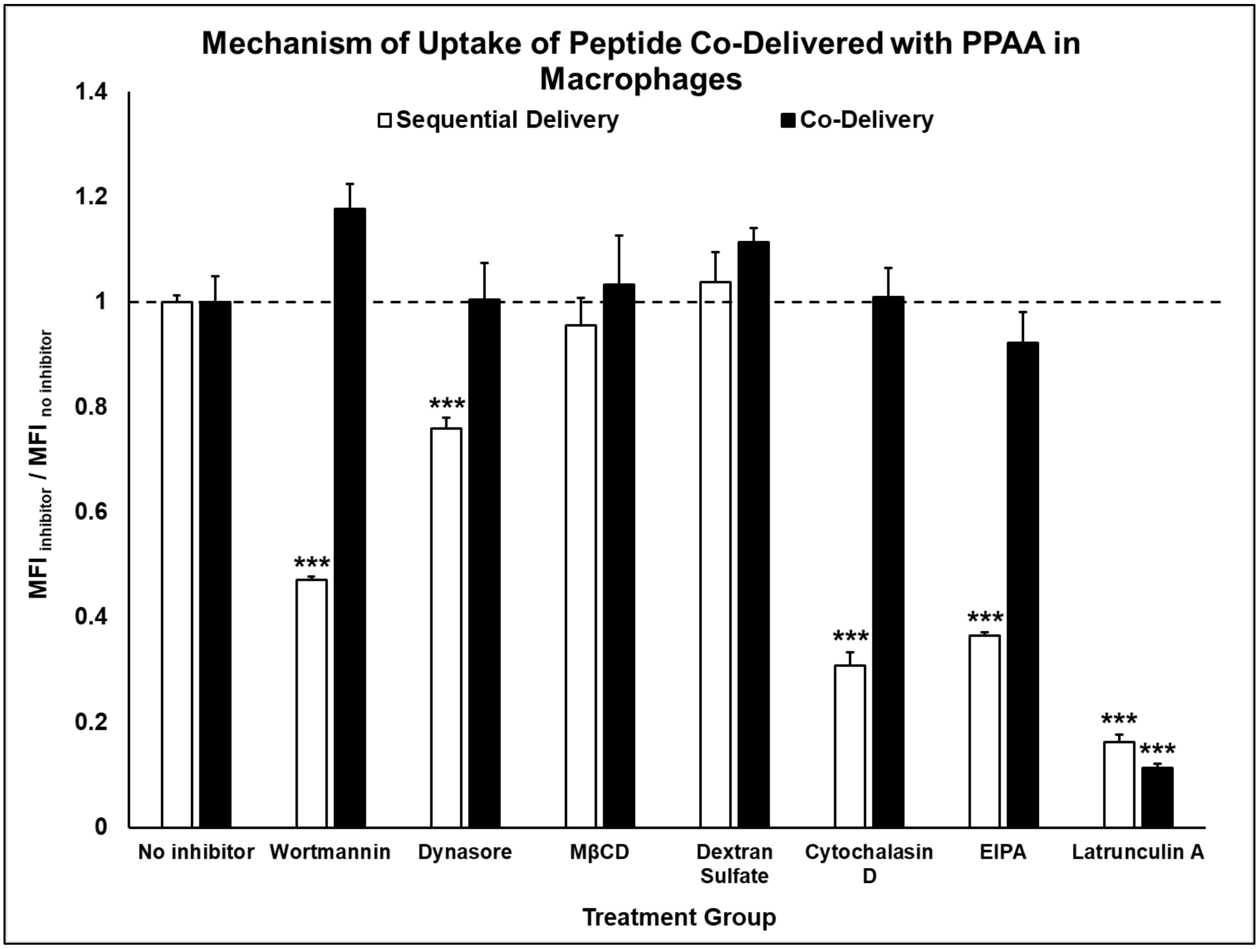

Supplementary Figure 14 – Mechanism of Uptake of Peptide Co-Delivered vs. Sequentially Delivered with PPAA in Murine Macrophages. Inhibition of YARA-MK2i peptide uptake when co-delivered vs. sequentially delivered with 5 µM PPAA in macrophages in the presence of various uptake inhibitors: micropinocytosis inhibitors – Wortmannin, Cytochalasin D, EIPA; clathrin-mediated endocytosis inhibitor – dynasore; lipid raft-mediated endocytosis inhibitor – methyl-β-cyclodextrin (MβCD); phagocytosis inhibitor – Latrunculin A. *$p<0.01$, **$p<0.05$, ***$p<0.001$ vs. no inhibitor; one-way ANOVA followed by Tukey's post-hoc test. Data are presented as means ± SEM.

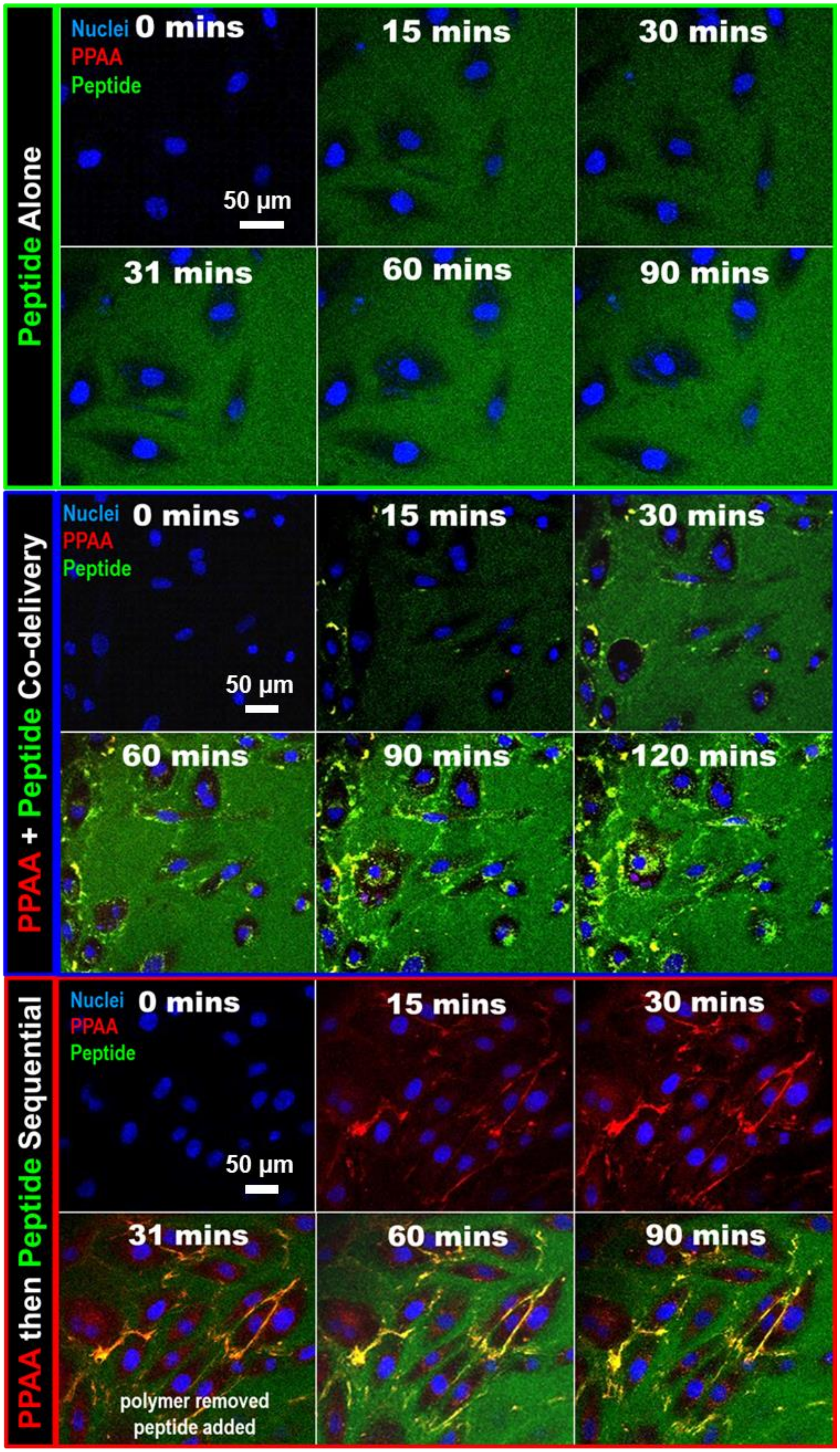
Peptide Alone
Nuclei
PPAA
Peptide
0 mins
15 mins
30 mins
50 µm
31 mins
60 mins
90 mins
PPAA + Peptide Co-delivery
Nuclei
PPAA
Peptide
0 mins
15 mins
30 mins
50 µm
60 mins
90 mins
120 mins
PPAA then Peptide Sequential
Nuclei
PPAA
Peptide
0 mins
15 mins
30 mins
50 µm
31 mins
60 mins
90 mins
polymer removed
peptide added

Supplementary Figure 15 - Visualization of PPAA mediated-peptide delivery through confocal fluorescence microscopy. Representative confocal fluorescence microscopy images of the PPAA polymer (red) and YARA-MK2i peptide (green) in rat A7R5 smooth muscle cells. The top row shows uptake after 30 minutes of co-delivery of the pre-complexed PPAA and YARA-MK2i peptide, whereas the bottom row shows uptake of sequentially delivered PPAA and then YARA-MK2i peptide. The red (PPAA) channel on the bottom row is after 30 minutes of treatment with the polymer alone and immediately prior to washing and addition of the peptide. The green channel (YARA-MK2i peptide) on the bottom row is immediately after addition of the peptide to the PPAA pre-treated cells, showing rapid concentration of the peptide onto polymer coated cell surfaces (see yellow colocalization in the merge channel).

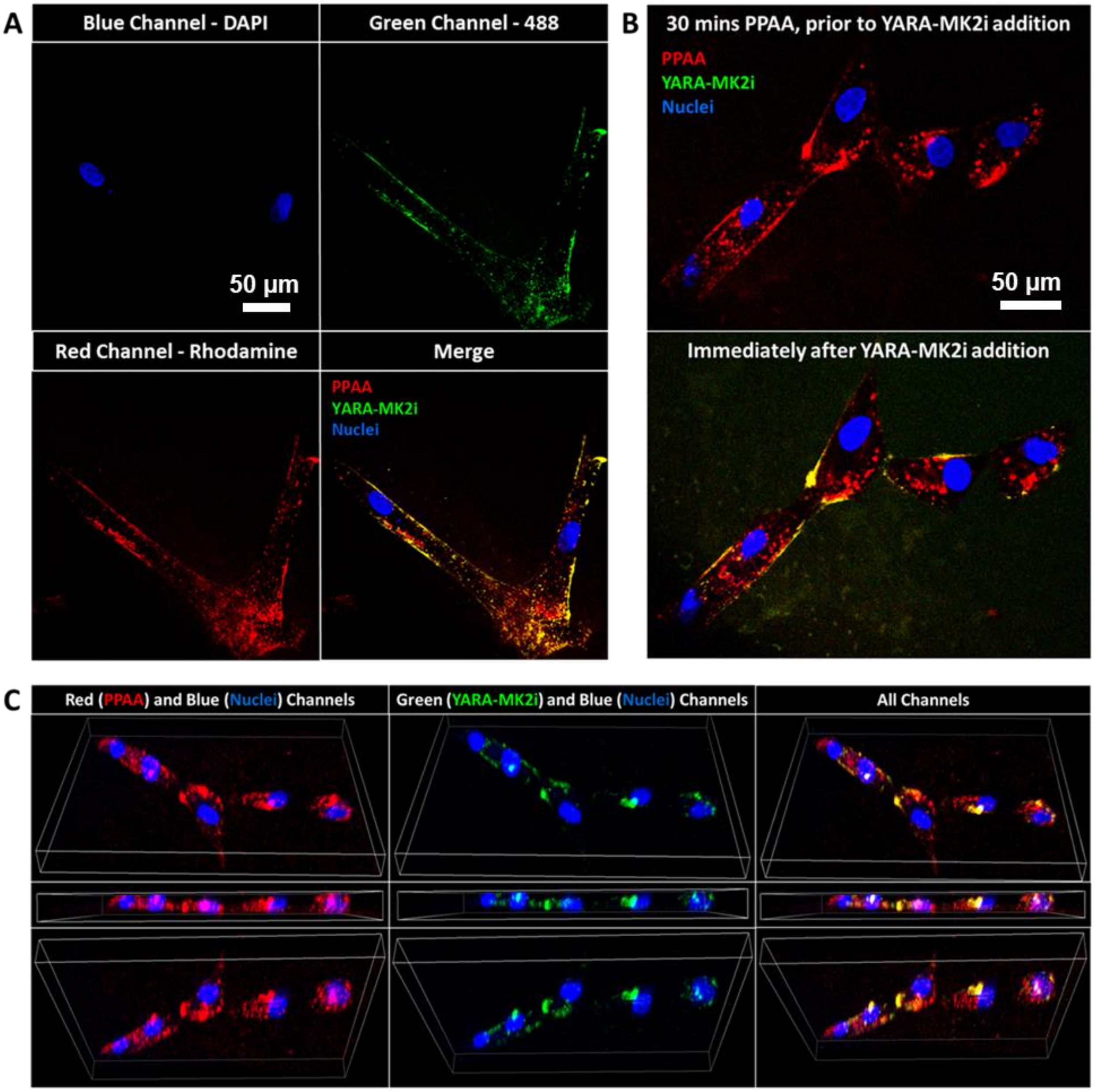

Supplementary Figure 16 - Visualization of PPAA mediated baiting of the YARA-MK2i peptide to the cell surface. A) Representative confocal fluorescence microscopy images of the PPAA polymer (red) and YARA-MK2i peptide (green) in human coronary artery vascular smooth muscle cells. Cells were imaged after sequential treatment with 5 µM rhodamine labeled PPAA for 30 minutes followed by treatment with 10 µM Alexa-488 labeled YARA-MK2i peptide for 30 minutes. B) Representative confocal fluorescence microscopy image slices of HCAVSMCs after 30 minutes of treatment with PPAA-RA immediately before and after removal of the PPAA-RA and addition of 10 µM Alexa-488 labeled YARA-MK2i peptide. C) 3-dimensional volume views of z-stack images taken of cells immediately after removal of the PPAA-RA treatment and addition of 10 µM Alexa-488 labeled YARA-MK2i peptide.

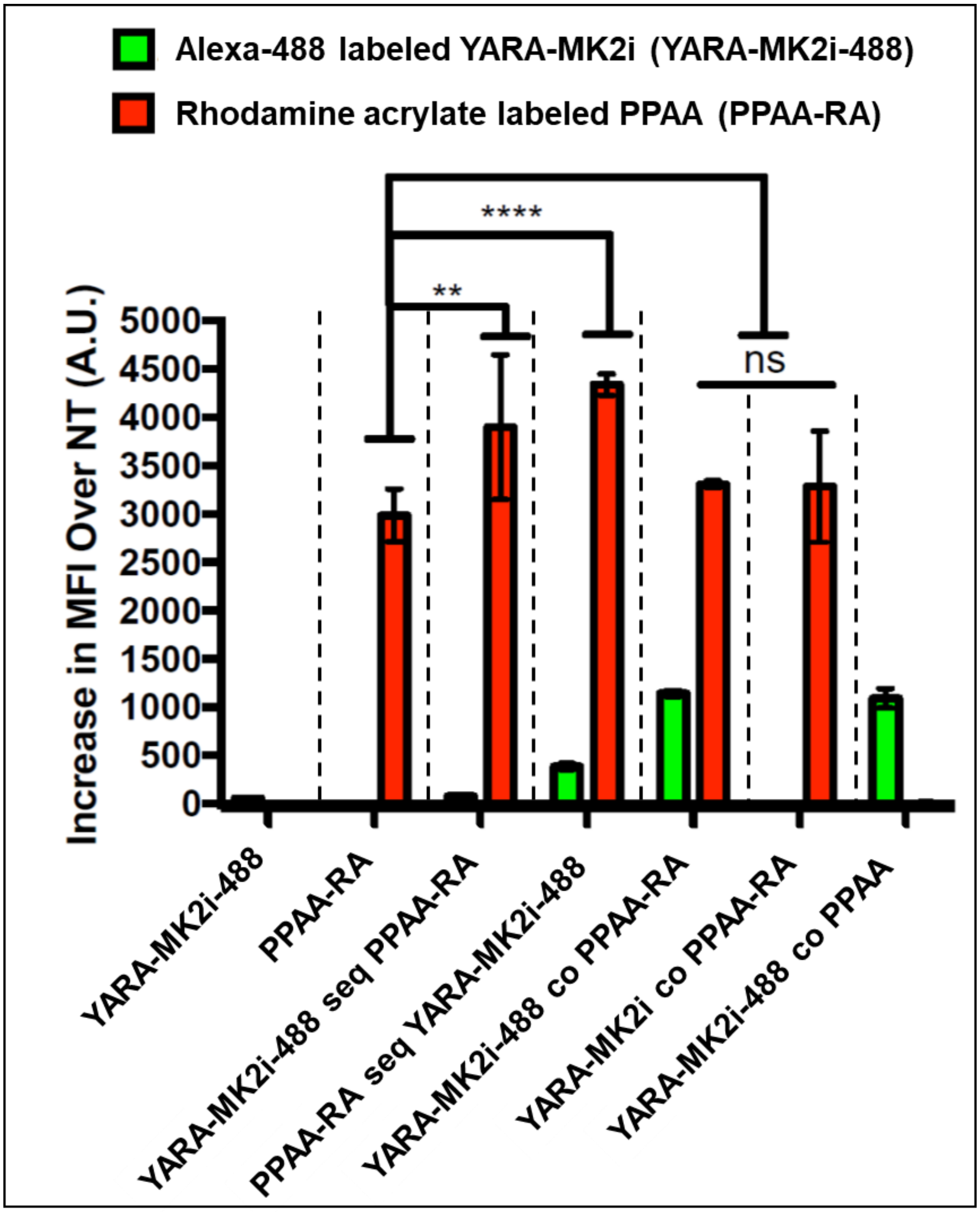

Supplementary Figure 17 – Flow cytometric analysis of fluorescently labeled polymer and peptide uptake. HCAVSMCs were treated with Alexa 488 labeled peptide alone (YARA-MK2i-488), rhodamine acrylate labeled PPAA alone (PPAA-RA), sequential (seq) treatment with YARA-MK2i-488 followed by PPAA-RA, sequential treatment with PPAA-RA followed by YARA-MK2i-488, YARA-MK2i-488 co-delivered (co) with PPAA-RA, unlabeled YARA-MK2i co-delivered with PPAA-RA, or YARA-MK2i-488 co-delivered with unlabeled PPAA. Treated cells were subsequently harvested and analyzed for labeled YARA-MK2i and labeled PPAA internalization. Each treatment was applied for 30 minutes, with sequential treatments having 30 minute treatment durations for both the polymer and peptide. *$p<0.01$, **$p<0.05$, ***$p<0.001$, ****$p<0.0001$; one-way ANOVA followed by Tukey's post-hoc test. Data are presented as means ± SEM.

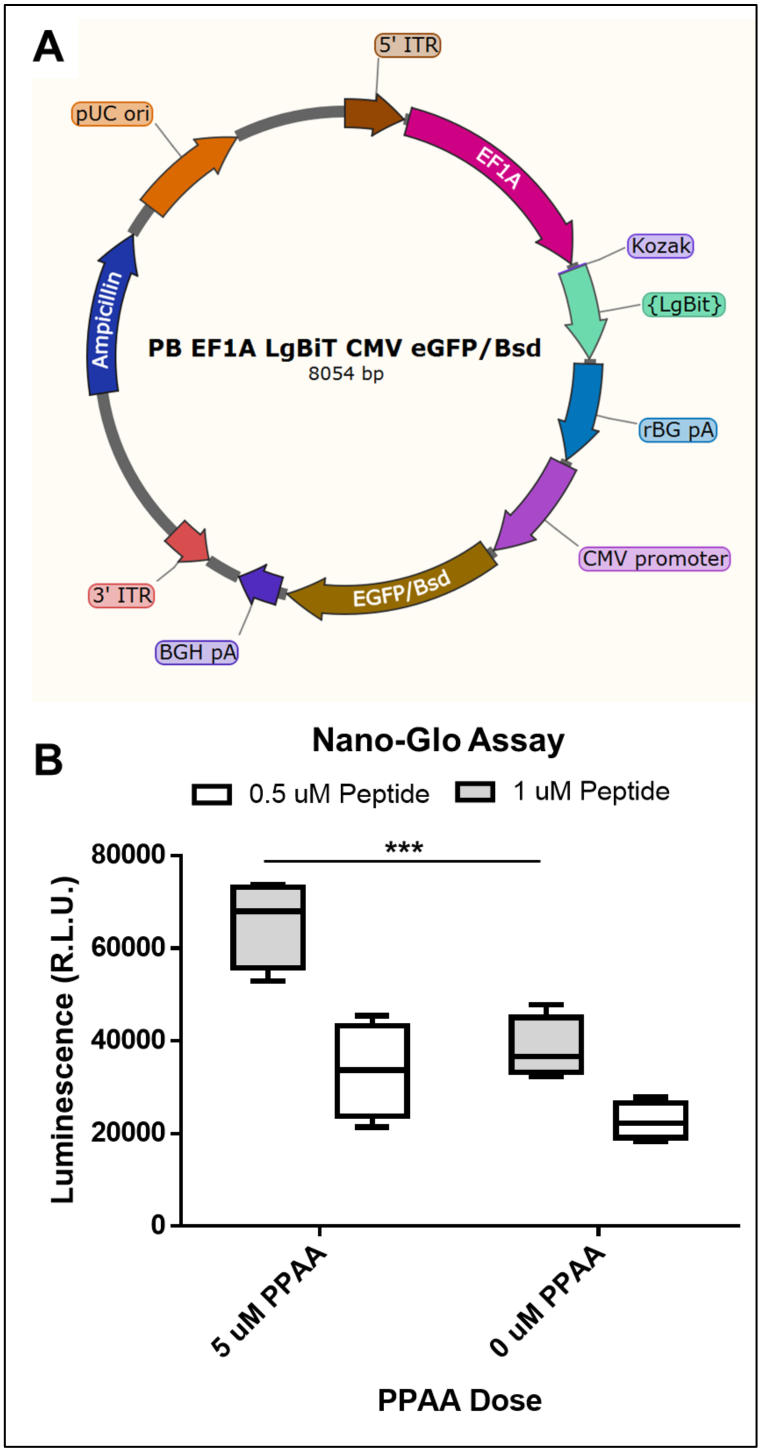

Supplementary Figure 18 – PPAA enhances CPP-modified HiBiT reporter activity in a LgBiT HEK 293-T cells. A) Map of the PB EF1A LgBiT CMV eGFP/Bsd plasmid utilized to generate the HEK 293-T LgBiT cell line. B) Luciferase signal from lgBiT HEK 293-T cells pre-treated with 0 or 5 μM PPAA followed by 0.5 or 1 μM YARA-HiBiT peptide. Two way ANOVA revealed that both the dose of PPAA and dose of peptide significantly affect luminescence ($p<0.001$ and $p<0.0001$, respectively). Post-hoc Sidak's multiple comparison testing: ***$p<0.0001$.

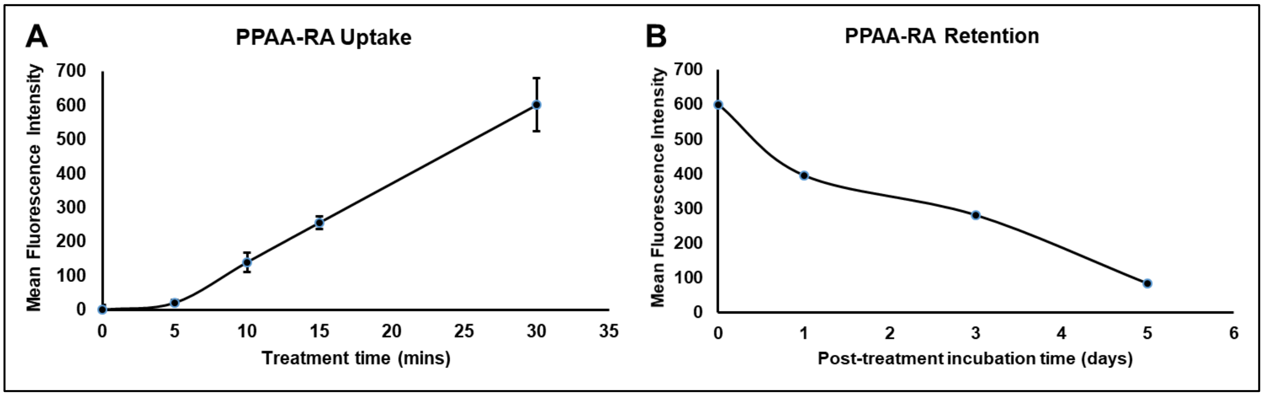

Supplementary Figure 19 – Kinetics of PPAA uptake and clearance. A) The kinetics of PPAA-RA uptake over a 30 minute treatment and B) retention of PPAA-RA over 5 days post-treatment in HCAVSMCs. [PPAA-RA] = 5 µM. Data are presented as means ± SEM.

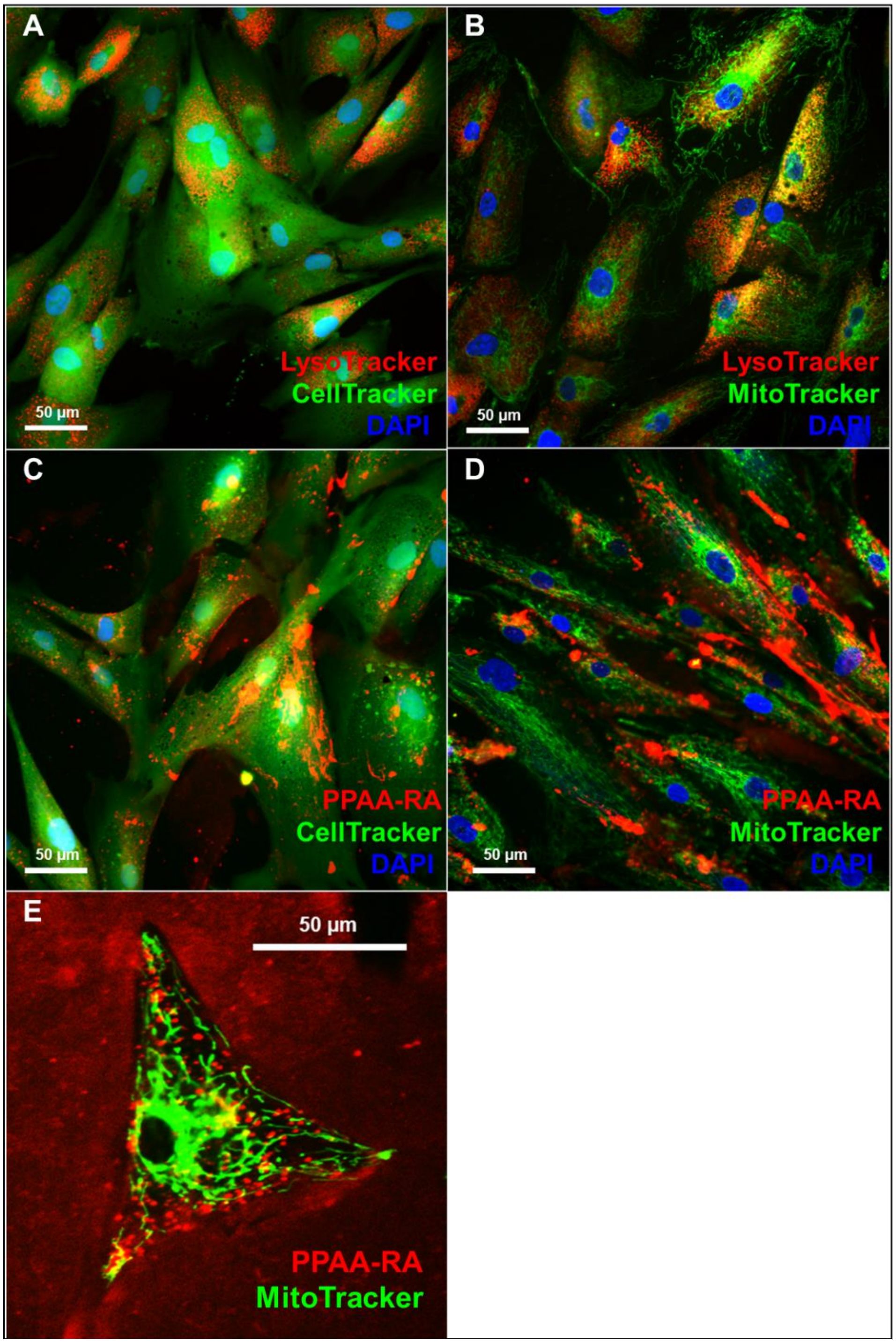

Supplementary Figure 20 – PPAA does not associate with mitochondria following internalization. Representative confocal microscopy images of A7r5 cells stained with LysoTracker endolysosomal dye and A) CellTracker cytosolic dye or B) MitoTracker mitochondrial dye (controls). Representative images of cells treated with PPAA-RA and C) CellTracker or D) MitoTracker. For A-D, nuclei were counterstained with DAPI. E) Zoomed image of a single cell treated with PPAA-RA and stained with MitoTracker.

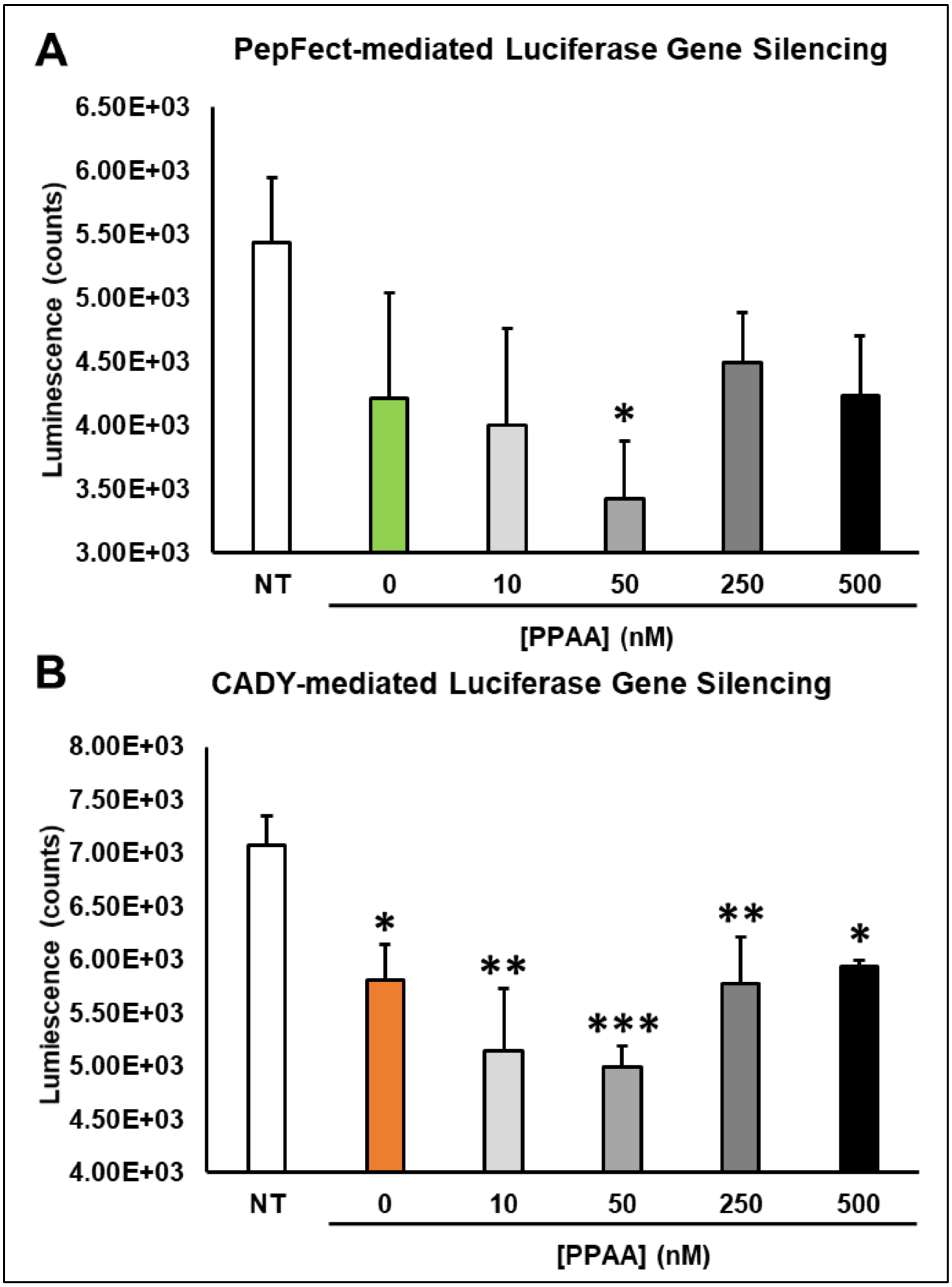

Supplementary Figure 21 –Gene silencing with sequential delivery of PPAA and peptide-siRNA complexes formed with PepFect and CADY. PPAA dose-dependent potentiation of luciferase gene silencing for siRNA delivery with A) PepFect and B) CADY. $*p<0.05$, $**p<0.01$, $***p<0.001$ vs. NT; one-way ANOVA followed by Tukey's post-hoc test. Data are presented as means ± SEM. Each treatment was applied for 30 minutes, and siRNA dose was 25 µM.

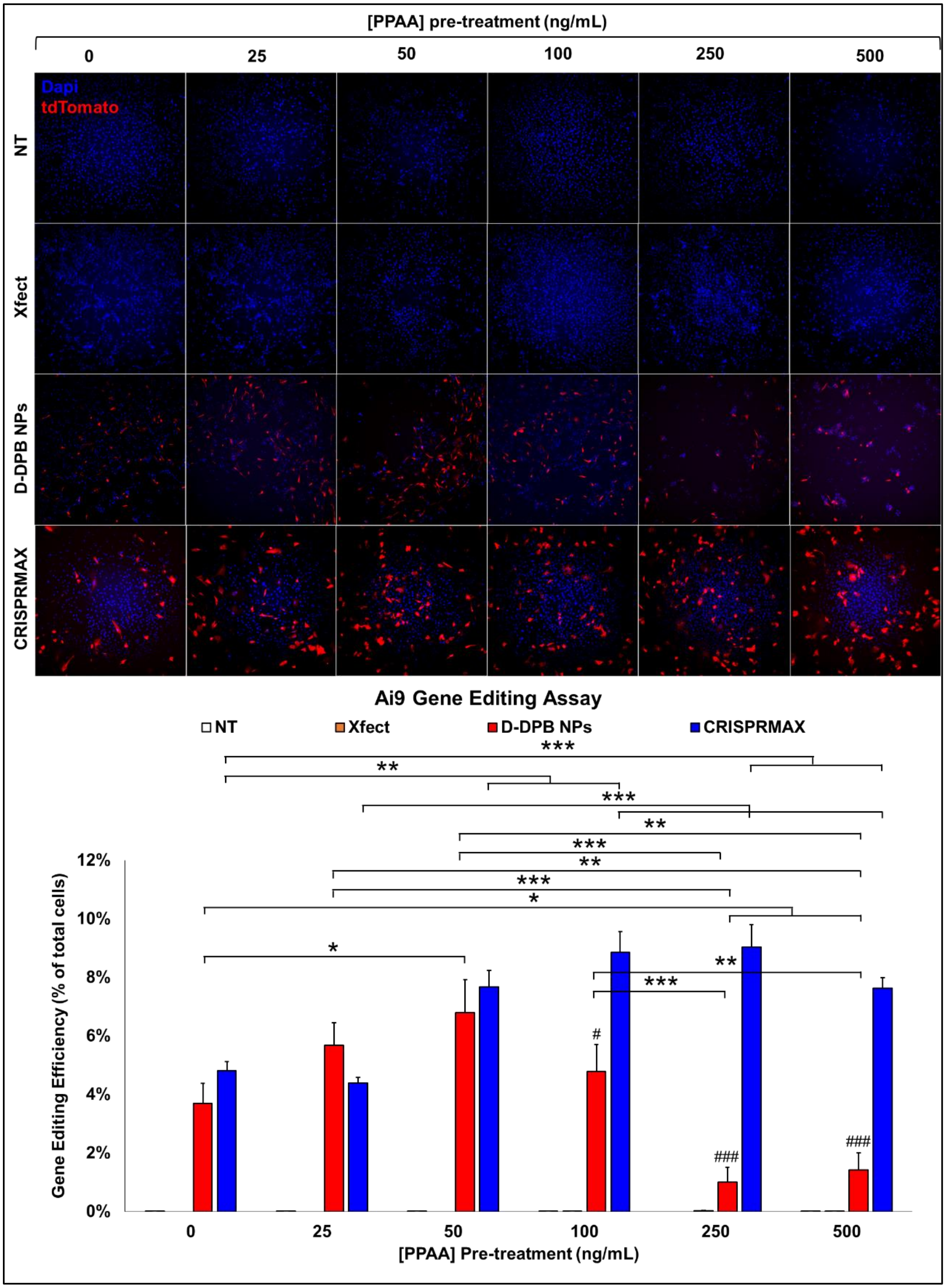

Supplementary Figure 22 – Microscopic analysis of gene editing efficiency. Representative microscopy images and quantification of gene editing efficiency with various delivery systems with and without PPAA pre-treatment (0, 25, 50, 100, 250, 500 ng/mL PPAA) in engineered Ai9 NIH/3T3 fibroblasts; *p<0.05. **p<0.01, ***p<0.001, #p<0.05, ###p<0.05 vs CRISPRMAX; one-way ANOVA followed by Tukey's post-hoc test. Data are presented as means ± SEM. All micrographs shown are 1404 x 1404 µm. 25, 50, 100 ,250 500 ng/mL = 1.1, 2.3, 4.6, 11.4, 22.7 µM PPAA, respectively.

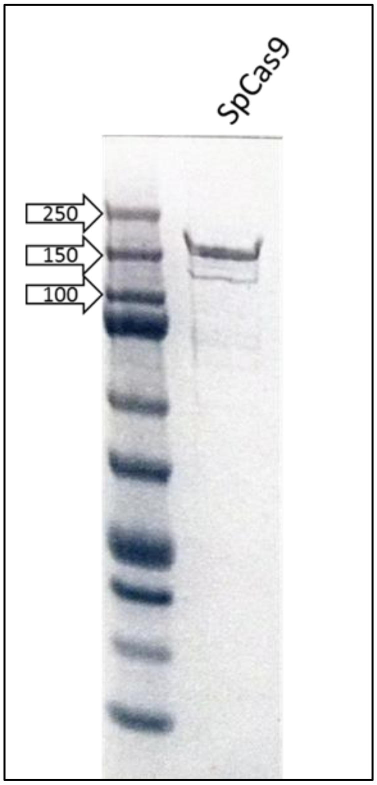

Supplementary Figure 23 – Gel image of purified SpCas9 protein (MW ~ 160 kDa).

**sgRNA primers**	
**Ai9-L**	TAA TAC GAC TCA CTA TAG Ggg AAA GAA TTG ATT TGA TAC CG GTT TTA GAG CTA GAA ATA G
**Ai9-R**	TAA TAC GAC TCA CTA TAG Ggg GTA TGC TAT ACG AAG TTA TT GTT TTA GAG CTA GAA ATA G
**Ai9 RosaRA**	TAA TAC GAC TCA CTA TAG Ggg GAC TGG AGT TGC AGA TCA CG GTT TTA GAG CTA GAA ATA G
**Ai9 RosaLA**	TAA TAC GAC TCA CTA TAG Ggg GAA GAT GGG CGG GAG TCT TC GTT TTA GAG CTA GAA ATA G
**gRNA universal primer**	AAA AAA GCA CCG ACT CGG TGC CAC

Supplementary Table 2 – sgRNA primer sequences. The in vitro transcribed sgRNAs were prepared using the listed forward and reverse primer. Sequence in blue is the T7 promoter, in red is the target sequence, and in black is the invariant sequence. The target sequences (20 nt in red) were sent to Synthego for production of sgRNAs with 2-O-methyl 3'phosphorothioate modifications in the first and last 3 nucleotides.
