# Supplementary material for: An anionic, endosome-escaping polymer to potentiate intracellular delivery of cationic peptides, biomacromolecules, and nanoparticles
Source: Nat Commun. 2019 Nov 1;10:5012. doi: 10.1038/s41467-019-12906-y (PMC6825215; doi:10.1038/s41467-019-12906-y)
Supplement: Supplementary file 2 — Reporting Summary [file 41467_2019_12906_MOESM2_ESM.pdf]

nature research

Corresponding author(s): Craig L. Duvall

Last updated by author(s): Sep 24, 2019

Reporting Summary

Nature Research wishes to improve the reproducibility of the work that we publish. This form provides structure for consistency and transparency in reporting. For further information on Nature Research policies, see Authors & Referees and the Editorial Policy Checklist.

Statistics

For all statistical analyses, confirm that the following items are present in the figure legend, table legend, main text, or Methods section.

n/a	Confirmed	
☐	☒	The exact sample size (n) for each experimental group/condition, given as a discrete number and unit of measurement
☐	☒	A statement on whether measurements were taken from distinct samples or whether the same sample was measured repeatedly
☐	☒	The statistical test(s) used AND whether they are one- or two-sided<br>*Only common tests should be described solely by name; describe more complex techniques in the Methods section.*
☒	☐	A description of all covariates tested
☐	☒	A description of any assumptions or corrections, such as tests of normality and adjustment for multiple comparisons
☐	☒	A full description of the statistical parameters including central tendency (e.g. means) or other basic estimates (e.g. regression coefficient) AND variation (e.g. standard deviation) or associated estimates of uncertainty (e.g. confidence intervals)
☐	☒	For null hypothesis testing, the test statistic (e.g. F, t, r) with confidence intervals, effect sizes, degrees of freedom and P value noted<br>*Give P values as exact values whenever suitable.*
☒	☐	For Bayesian analysis, information on the choice of priors and Markov chain Monte Carlo settings
☒	☐	For hierarchical and complex designs, identification of the appropriate level for tests and full reporting of outcomes
☒	☐	Estimates of effect sizes (e.g. Cohen's d, Pearson's r), indicating how they were calculated

Our web collection on statistics for biologists contains articles on many of the points above.

Software and code

Policy information about availability of computer code

Data collection	ImageExpress, VWorks Automation Control Software, NIS Elements V4.0, Astra V, Guava InCyte V3.3.
Data analysis	ImageJ V1.50i, Just another coloacalization program (JACoP), ImageExpress, Graphpad Prism, NIS Elements, FlowJo V 10.1, Microsoft Excel 2013.

For manuscripts utilizing custom algorithms or software that are central to the research but not yet described in published literature, software must be made available to editors/reviewers. We strongly encourage code deposition in a community repository (e.g. GitHub). See the Nature Research guidelines for submitting code & software for further information.

Data

Policy information about availability of data

All manuscripts must include a data availability statement. This statement should provide the following information, where applicable:

- Accession codes, unique identifiers, or web links for publicly available datasets
- A list of figures that have associated raw data
- A description of any restrictions on data availability

The datasets generated during and/or analysed during the current study are available from the corresponding author on reasonable request.

Field-specific reporting

Please select the one below that is the best fit for your research. If you are not sure, read the appropriate sections before making your selection.

☒ Life sciences ☐ Behavioural & social sciences ☐ Ecological, evolutionary & environmental sciences

Life sciences study design

All studies must disclose on these points even when the disclosure is negative.

Sample size	Sample sizes were chosen based on preliminary data demonstrating statistically significant differences for each specific assay.
Data exclusions	Data from gene editing and galectin 8 assays were excluded for samples with insufficient cell number (<500 cells) and for samples that did not receive proper treatment volumes due to errors in dispensing / aspirating with the bravo robot.
Replication	All experiments were performed with at least three technical replicates on more than one occasion to ensure reproducibility across experiments.
Randomization	Randomization and covariates were not relevant to our study design as we investigated single factors within each study.
Blinding	Blinding was not relevant as all processing methods were done through available software with consistent parameters utilized across all treatment groups.

Reporting for specific materials, systems and methods

We require information from authors about some types of materials, experimental systems and methods used in many studies. Here, indicate whether each material, system or method listed is relevant to your study. If you are not sure if a list item applies to your research, read the appropriate section before selecting a response.

Materials & experimental systems

n/a	Involved in the study	
☒	☐	Antibodies
☐	☒	Eukaryotic cell lines
☒	☐	Palaeontology
☒	☐	Animals and other organisms
☒	☐	Human research participants
☒	☐	Clinical data

Methods

n/a	Involved in the study	
☒	☐	ChIP-seq
☐	☒	Flow cytometry
☒	☐	MRI-based neuroimaging

Eukaryotic cell lines

Policy information about cell lines

Cell line source(s)	American Type Culture Collection (ATCC) and Lonza
Authentication	Cells were purchased from and authenticated by their respective supplier. None of the cells were derived in-house.
Mycoplasma contamination	All cells tested negative for mycoplasma and were expanded in media containing a mycoplasma prophylactic (plasmocin)
Commonly misidentified lines (See ICLAC register)	No commonly misidentified cell lines were utilized in these studies.

Flow Cytometry

Plots

Confirm that:

☒ The axis labels state the marker and fluorochrome used (e.g. CD4-FITC).

☒ The axis scales are clearly visible. Include numbers along axes only for bottom left plot of group (a 'group' is an analysis of identical markers).

☒ All plots are contour plots with outliers or pseudocolor plots.

☒ A numerical value for number of cells or percentage (with statistics) is provided.

Methodology

Sample preparation	Depending on the experiment, HCAVSMCs, HMVECs, RAW 264.7, MCF7, HEK293T, or A7r5 cells were grown to 80-90% confluence, harvested, and seeded at 60,000 cells/well in a 12 well plate and allowed to adhere overnight. Cells were treated as defined in the relevant materials and methods sections in Opti-MEM medium supplemented with 1% penicillin-streptomycin and 1% FBS. For all flow cytometry assays, following treatment or post-treatment incubation, cells were washed 2x in PBS without

calcium or magnesium, harvested with 0.05% trypsin-EDTA, centrifuged, resuspended in 300 µL of 0.05% Trypan blue in PBS without calcium or magnesium, and transferred to a 96 well plate for analysis on an EMD Millipore Guava easyCyte™ 5HT flow cytometer with InCyte software for data acquisition.

Instrument	EMD Millipore Guava easyCyte™ 5HT flow cytometer
Software	FlowJo V10.1
Cell population abundance	Cells were run to achieve > 10,000 events in the gated cell population
Gating strategy	Gating was performed based on identifying a distinct population in FSC vs SSC plots.

☒ Tick this box to confirm that a figure exemplifying the gating strategy is provided in the Supplementary Information.
