# Supplementary material for: An anionic, endosome-escaping polymer to potentiate intracellular delivery of cationic peptides, biomacromolecules, and nanoparticles
Source: Nat Commun. 2019 Nov 1;10:5012. doi: 10.1038/s41467-019-12906-y (PMC6825215; doi:10.1038/s41467-019-12906-y)
Supplement: Supplementary file 3 — Description of Additional Supplementary Files [file 41467_2019_12906_MOESM3_ESM.pdf]

Description of Additional Supplementary Files

File Name: Supplementary Movie 1
Description: Time-lapse confocal microscopy of co-delivery of PPAA and YARA-MK2i peptide. HCAVSMCs were treated with pre-complexed rhodamine acrylate labeled PPAA (PPAA-RA, red) and Alexa-488 labeled YARA-MK2i peptide (A488-YARA-MK2i, green). Treated cells were imaged once every 10 seconds for hour starting immediately prior to treatment. PPAA-RA dose = 2.5 µM; A488-YARA-MK2i dose = 10 µM.

File Name: Supplementary Movie 2
Description: Time-lapse confocal microscopy of sequential delivery of PPAA followed by the YARA-MK2i peptide. HCAVSMCs were sequentially treated with PPAA-RA for 30 minutes followed by A488-YARA-MK2i for 30 minutes. Treated cells were imaged once every 10 seconds for hour starting immediately prior to treatment. PPAA-RA dose = 2.5 µM; A488-YARA-MK2i dose = 10 µM.

File Name: Supplementary Movie 3
Description: Time-lapse confocal microscopy of YARA-MK2i peptide uptake. HCAVSMCs were treated with A488-YARA-MK2i alone. Treated cells were imaged once every 10 seconds for hour starting immediately prior to treatment. PPAA-RA dose = 2.5 µM; A488-YARA-MK2i dose = 10 µM.

File Name: Supplementary Movie 4
Description: Time-lapse confocal microscopy of PPAA colocalization with the autophagosomal marker LC3B. HEK 293-T cells stably expressing LC3B-mTurquoise2 (green) treated with rhodamine labeled PPAA (PPAA-RA, purple). PPAA-RA dose = 5 µM.
